# Bounded Confidence and Cohesion-Moderated Pressure: A General Model for the Large-Scale Dynamics of Ordered Opinion

**DOI:** 10.3390/e25081219

**Published:** 2023-08-16

**Authors:** Fangyikuang Ding, Yang Li, Kejian Ding

**Affiliations:** 1 Department of Sociology, East China University of Science and Technology, Shanghai 200030, China; 20003609@mail.ecust.edu.cn; 2 School of Physical Science and Engineering, Beijing Jiaotong University, Beijing 100044, China

**Keywords:** opinion, synchronization, consensus, chaos, cohesion, bounded confidence

## Abstract

Due to the development of social media, the mechanisms underlying consensus and chaos in opinion dynamics have become open questions and have been extensively researched in disciplines such as sociology, statistical physics, and nonlinear mathematics. In this regard, our paper establishes a general model of opinion evolution based on micro-mechanisms such as bounded confidence, out-group pressure, and in-group cohesion. Several core conclusions are derived through theorems and simulation results in the model: (1) assimilation and high reachability in social networks lead to global consensus; (2) assimilation and low reachability result in local consensus; (3) exclusion and high reachability cause chaos; and (4) a strong “cocoon room effect” can sustain the existence of local consensus. These conclusions collectively form the “ideal synchronization theory”, which also includes findings related to convergence rates, consensus bifurcation, and other exploratory conclusions. Additionally, to address questions about consensus and chaos, we develop a series of mathematical and statistical methods, including the “energy decrease method”, the “cross-d search method”, and the statistical test method for the dynamical models, contributing to a broader understanding of stochastic dynamics.

## 1. Introduction

### 1.1. Opinion Evolution: Micro-Mechanisms and Macroscopic Issues

In recent years, owing to the rapid advancement of social media and online communities, we have witnessed a remarkable phenomenon: the potency of opinion evolution frequently sparks intricate public opinion events and mobilizes collective action. This captivating dynamic, intertwined with aspects such as group decision-making and related concerns, has emerged as a prominent area of research in the field of opinion dynamics. In this area, ordered opinions, which include common types such as political perspectives and emotional intensity, have garnered significant attention. Existing literature addressing this matter has primarily focused on examining the micro elements of macro consequences, such as consensus [1], thereby establishing a fundamental problem awareness in statistical physics. Furthermore, this research can effectively engage in constructive discourse with social influence theory and agent-based modeling methods in the domain of mathematical sociology [2,3,4,5]. Drawing on these foundations, certain studies in nonlinear dynamics and social learning theory have directly formulated update rules for individual opinions based on empirical assumptions, such as “interpersonal imitation” and “Bayesian updating” [6,7]. To elucidate the reliability of such micro assumptions, our objective is to comprehensively review behavioral science and communication studies concerning several mechanisms, which encompass the “bounded confidence hypothesis”, “out-group pressure mechanism”, and “in-group cohesion mechanisms” (their meanings will be explained below). This endeavor not only helps us to present the problem awareness of our paper but also establishes a solid groundwork for subsequent modeling in the ensuing sections.

Firstly, within a particular social context, individuals often adhere to a “bounded confidence” mechanism when deciding whether to engage in an interaction. In the realm of opinion dynamics models, this can be simplified as a hypothesis: individual i possesses a confidence threshold d and only engages with j at time t + 1 if the difference between j’s opinion value at time t and i’s opinion value at time t falls below d(|x_i_(t) − x_j_(t)| < d) [8]. To establish the reliability of this mechanism, we can direct our attention to some empirical research on selective exposure and echo chambers in communication studies, which shows people tend to contact and understand information similar to their cognitions and opinions because bounded confidence serves as its idealized manifestation. This body of literature scrutinizes discourses on platforms like Twitter, Facebook, and Reddit through online experiments and data mining, illustrating that many individuals exhibit a strong inclination to interact with content that aligns with their own, such as by commenting and sharing [9,10]. Simultaneously, some scholars have identified that people give more weight to nearby opinions while forming their opinions based on behavior experiments [11]. These findings can substantiate the applicability of the “bounded confidence hypothesis” in large-scale social networks.

Secondly, having explored the local groups formed by bounded confidence/echo chambers and their consequences, we can categorize people into in-groups and out-groups based on a fixed agent. For a given agent i, the former encompasses “current interactors” and “frequent interactors” of i, a phenomenon explicable by the localized bounded confidence mechanism. The latter represents other individuals within social networks who are numerous and more likely to exert overall social pressure/out-group pressure. Taking Facebook as an illustration, individual i engages in conversations with j1… jn at time t(in-groups) while simultaneously perusing a substantial volume of content posted by unfamiliar individuals (out-groups). Both aspects can influence individual i’s opinion state at time t + 1, and the effect from out-groups can be called as out-group pressure. In this regard, certain literature in behavioral science and opinion dynamics regards the source of pressure as a composite of multiple individuals’ states, such as the average of opinions held by all out-group users, thereby revealing its “intervention” effect on individual cognition and decision-making. Consequently, the existence of “out-group pressure mechanisms” (or “social pressure”) is verified [12,13].

Thirdly, to exemplify the extent of influence exerted by the aforementioned mechanisms on individual opinions, an exploration of the interplay between the in-group/bounded confidence mechanism and the out-group/social pressure mechanism is warranted. This interaction is pertinent to the “group cohesion” observed within small groups (abbreviated as cohesion), and the meaning of this concept is the degree to which group members coordinate and maintain consistency with each other. In this regard, it is imperative to integrate the realms of psychology and communication to explain relevant mechanisms. Within the field of psychology, research on cohesion can be classified into two distinct categories. Firstly, it centers on the definition and quantification of cohesion, primarily encompassing individuals’ identification with the group and similarity among group members. However, this endeavor encounters substantial ambiguity and lacks a universally accepted consensus [14]. Secondly, it delves into investigating the moderating impact of in-group cohesion on out-group pressure, wherein it diminishes individuals’ perception of pressure, leading them to resist external opinions and succumb to “group blindness [15]”. Therefore, the role of social pressure mechanisms in opinion evolution can be mitigated. Regarding communication studies, emphasis is placed on the attribute of the “filter bubble” within small groups in social networks [16]. This concept suggests that when an individual exclusively receives homogeneous information, their exposure to divergent opinions becomes limited [17]. Extensive empirical data has validated this phenomenon, with a significant focus placed on the “information/opinion homogeneity” of group members. This perspective serves as a vital factor in assessing cohesion during the evolution of opinions. In this context, the similarities among opinions within the in-group can serve as a metric for measuring the strength of cohesion, and we will obtain the “out-group pressure” moderated by “in-group cohesion mechanisms” through the method.

In addition, building upon the preceding discussion on the foundational mechanisms governing interactions between individuals, we shall augment the existing understanding with two inconclusive micro/intermediate mechanisms (depending on individuals’ attributes). This endeavor seeks to construct a comprehensive portrayal of opinion evolution, facilitating the transition towards overarching macroscopic issues. Within this framework, the micro-mechanism manifests as follows: when individual i engages with individual j, they engage in reciprocal communication and imitation, thereby converging their opinions. Alternatively, they may elect to maintain their respective opinions unaltered or actively oppose one another, precipitating a polarization of attitudes [18]. On the other hand, the intermediate mechanism revolves around the premise that within a given context (such as platforms for mutual attention or dissemination), the likelihood of “individual i interacting with individual j” varies contingent upon social network factors. This notion elucidates the essence of “large-scale”, as denoted in the title of our scholarly contribution, signifying the presence of a multitude of unfamiliar relationships within the network. Consequently, this engenders a distinctive manifestation of large-scale opinion dynamics that is different from localized dynamics.

Arising from the elucidated micro and intermediate mechanisms, we frequently encounter three distinct macroscopic phenomena: global consensus, local consensus, and chaos. Global consensus denotes the convergence of all individual opinions within the system, culminating in a nearly homogeneous state (i.e., the final count of opinion categories (Opinion categories refer to the several kinds of opinions that the system converges to when time approaches infinity.) represented by N (consensus) equals 1, and it’s also called as “consensus categories” below). Local consensus signifies the convergence of opinions within the system towards multiple states (i.e., 1 < N (consensus) < n, where n represents the number of individuals). Lastly, chaos manifests when individual opinions fail to converge, thereby evincing intricate fluctuations. These phenomena shall be rigorously defined in the preliminary sections of our manuscript.

In reference to the micro-mechanisms and macroscopic phenomena, we aim to respectively elucidate the problem awareness: 1. Probing the sufficient conditions for the emergence of a global consensus within the opinion system (the question 1); 2. Delving into the conditions for attaining the local consensus within the opinion system (the question 2-1), and also investigating the mechanisms (Conditions and mechanisms are two different concepts in sociology. Condition refers to the factors that cause consequences, and mechanism refers to the way in which conditions act on the results (usually represented as agent action combinations and system processes).) that impede its transformation into a global consensus (the question 2-2); 3. Exploring the conditions for the opinion system to enter the chaos (the question 3-1) and mechanisms that hinder its transformation into a local consensus (the question 3-2). It is important to note that the aforementioned conditions in each of the 3 cases pertain to “individuals’ attributes” or “the potency of particular micro-mechanisms”, while the conclusions evidently represent macroscopic outcomes, such as consensus.

To address these questions effectively, we must pose explicit steps/problems that warrant attention. Regarding this, we can solve three problems to answer questions 1/2/3: 1. Presenting a formal model that accurately depicts the intricacies of an opinion interaction on a micro level (Problem A); 2. Scrutinizing the conditions and outcomes within this formal model through mathematical deductions and numerical simulations to unravel the laws and mechanisms underlying consensus and chaos generation (Problem B); 3. Utilizing data from social media to infer and validate the model parameters, thereby substantiating their alignment with real-world processes (Problem C). We will review the existing solutions to problems A/B/C in Section 1.2.1, Section 1.2.2 and Section 1.2.3, respectively.

### 1.2. General Model: Bounded Confidence and Its Properties

#### 1.2.1. Modeling

In the realm of problem A, scholars in social dynamics have undertaken numerous endeavors to model the fluctuations in opinions based on the aforementioned micro-mechanisms. These models can be broadly classified into two categories, depending on the specific context being explored. When investigating offline social dynamics and discrete value/category perspectives, prominent models like “voter” assume a vital role in transforming individual interactions into a collective decision-making process, enabling effective explanations and predictions of political behaviors, such as citizen elections [19]. Conversely, models like bounded confidence/social influence demonstrate remarkable reliability when examining the continuum/ordered opinions exchanged online [19]. These models primarily emphasize large-scale assimilation mechanisms and can be effectively analyzed within the context of social networks, which is precisely the focal point of our scholarly investigation and will be elaborated upon in subsequent sections.

Within the framework of the bounded confidence hypothesis, two widely accepted formal models are the H-K model and the D-W model. While these models differ in terms of the mechanisms governing the evolution of opinions, they both exhibit robust convergence and synchronization. Specifically, the H-K model is well-suited for scenarios involving open and conversational opinion interactions, such as Reddit questions or Twitter posts. Its updating strategy involves individuals computing the arithmetic mean of others’ opinions within their neighborhood. The basic formulation of the H-K model is the following: Equation (1) [6]. On the other hand, the Deffuant model operates within the context of pairwise communication between individuals, such as Facebook conversations, and incorporates significant stochastic attributes. It utilizes the weighted average of opinions between pairs of individuals to update the original opinion. The basic representation of the Deffuant model is as shown in the following Equation (2) (where xi(t) denotes the opinion of individual i at time t):

(1) $$x_{i}(t+1)=\frac{\sum_{j:\left\|x_{i}\left(t\right)-x_{j}(t)\right\|\leq 1}^{}x_{j}(t)}{\sum_{j:\left\|x_{i}\left(t\right)-x_{j}(t)\right\|\leq 1}^{}1}$$

(2) $$\begin{array}{l} x_{i}(t+1)=x_{i}(t)+u\left[x_{j}(t)-x_{i}(t)\right]\\ x_{j}(t+1)=x_{j}(t)+u\left[x_{i}(t)-x_{j}(t)\right] \end{array}$$

In the classic HK and DW frameworks, the assumptions of individual homogeneity, environmental certainty, and other characteristics are far from reality. Recently, scholars have weakened these assumptions and presented complexity characteristics in the following ways (written as strategy 1/2/3/4): (1) they have studied heterogeneous bounded confidence models, which require different confidence thresholds (d_i_) and individual learning rates (u_i_) [20]; and (2) they have emphasized the asymmetric opinion updating mechanism. In the classic Deffuant model, paired individuals often update their opinions simultaneously. However, this assumption can be weakened to “individual i updates their own opinion based on individual j, but individual j does not update based on individual i” [21]. This adjustment is suitable for large-scale social networks where individual i browses individual j’s tweets; (3) They have introduced stochastic noise to add uncertainty to the HK model and incorporate global effects into opinion transitions. Individuals are not only influenced by opinions within the confidence threshold but also closely related to attitudes in the overall environment [22,23]; (4) They have added stubborn agents [24,25] with unchanged opinions into the model and have explained the formation of opinion polarization through simulation and empirical results [26].

Some of these revision strategies are closely related to the psychological mechanisms [27,28] mentioned earlier and can be included in the general model of this article. For strategy (1), the subsequent framework will incorporate heterogeneity to adapt to complex individual behaviors. For strategy (3), considering the limited research on group pressure in opinion evolution and the neglect of its transmission effect, we can choose an appropriate expression for random noise to represent the effect of out-group pressure on individual interactions and regulate this effect through in-group cohesion. As for strategy (4), existing models often lack social exclusion effects (u_i_ is less than 0). Therefore, our paper will consider this special condition as a supplement to “stubborn agents”, who are non-assimilatory and may have consequences that differ from synchronization. In general, the integration of different mechanisms (by revision strategies of the D-W/H-K models) can construct a new model close to reality, serving as the solution to problem A.

#### 1.2.2. Synchronization Research

For problem B, the pertinent literature has extensively investigated the synchronization of bounded confidence models (the meaning of synchronization is the same as the global consensus aforementioned in the field of opinion dynamics); however, the mechanisms behind chaos remain insufficiently understood. Regarding numerical simulation, some scholars have not only explored the sufficient conditions for the formation of global consensus (such as d > 1/2) but have also simulated the number of consensus categories and convergence rates under various parameter conditions [29,30]. These endeavors yield reliable hypotheses and conjectures for subsequent theorem proofs while unearthing critical values and “statistical laws” governing model phase transitions.

When it comes to proving the synchronization theorems, the H-K and D-W frameworks offer two representative approaches. Under the original conditions (i.e., as per the model proposed), researchers have demonstrated the following theorem: “individual opinions either synchronize or diverge by more than the confidence threshold d” [31]. This conclusion possesses inherent intuitiveness: the governing rules of both model types effectively capture the process of opinion assimilation, and when there is a global or local consensus, it signifies a convergence of opinions. Achieving such convergence necessitates that interactions falling within the confidence threshold exert minimal influence on opinion values. However, multiple avenues exist to substantiate this claim, and through rigorous mathematical analysis, one can derive a plethora of insightful conclusions (theorems).

In terms of existing methodologies, they can be broadly classified into three distinct categories: (1) Predominantly centered on the inherent characteristics of the model, these approaches leverage mathematical techniques such as scaling and probability approximation for processing. For instance, within the context of H-K, synchronization outcomes can be derived through inductive reasoning based on the order-preserving property of xi(t) under the update rule F or by considering the non-decreasing and non-increasing attributes of the upper and lower bounds [31]. (2) Regarding bounded confidence models as dynamical systems, researchers draw upon conclusions and methodologies from various fields, including topological methods and ergodic theory. An example involves treating D-W as a random flow, exploring its stability and attractiveness, and subsequently formulating synchronization conditions and establishing proofs [32]. (3) Models such as D-W/H-K might view uncertainty as a perturbation to the control system, whereby the analysis of the stochastic system can undergo a transformation into the design of a stochastic control algorithm. This framework, pioneered by Chen, Su, et al. [33], has been introduced and partially developed.

In terms of the existing theorems, within the realm of homogeneous models (where u1 = … = un = u, d1 = … = dn = d), the convergence of the H-K model has been extensively addressed, attaining either synchronization or convergence towards multiple opinion values (with a difference between the convergence values greater than d) [31]. The D-W model can be categorized into symmetric and asymmetric cases (i.e., whether i/j updates opinions in pairs). The former guarantees a synchronization probability of 1 when d > 1/2, while the latter has convergence rate-based findings presented by Chen et al. [34,35]. Within the context of heterogeneity models (where individual d_i_/u_i_ differ from one another), several scholars have demonstrated that their convergent features are similar to those of homogeneous models, and global synchronization can also be achieved when maxd_i_ = 1 [34,36].

The above methods are highly skilled and can be extended to the synchronous proof of numerous opinion dynamics models. In these methods, the idea of understanding the model as a dynamical system and proving synchronization through studying its geometric properties is worth noting, which is expected to be used for solving problem B and further developed in our paper.

#### 1.2.3. Data Validation: Random Optimization and Bayesian Approaches

For problem C, it is imperative to broaden our perspective on the validation process of various dynamic models, which can be effectively applied to the model presented in this paper. This can be divided into parameter estimation of the model (obtaining unknown parameter values) and theoretical testing of models with specific parameter values (verifying the model’s capacity to explain the real world). The literature on the former (estimation) is relatively rich, while the methods of the latter (test) are still immature, with only some empirical attempts. In this regard, I will focus on reviewing the methods related to the former and providing a few introductions to the practice of the latter.

In regard to parameter estimation of dynamic models (such as opinion dynamics), existing methods can be classified into three distinct categories: (1) direct assignment of values by leveraging the structural information of parameters; (2) utilization of optimization techniques to search for parameters that minimize the disparity between simulation results and empirical data; and (3) utilization of sample/prior information to statistically infer parameters.

Regarding (1), certain literature has obtained numerical values about the number of agents/interaction attributes in the model through experimental design or empirical investigation [37,38]. However, due to the sensitivity of opinion updates to environmental conditions, it is challenging for researchers to design universally applicable experimental scenarios and directly employ measured values (e.g., social learning rate u or confidence threshold d) as parameter estimates in the model. As for (2), its fundamental concept revolves around measuring the “distance” between simulated data and real data, thereby identifying the optimal parameter values that sufficiently minimize this discrepancy through appropriate optimization methods. This approach frequently relies on intelligent algorithms and stochastic optimization techniques, such as particle swarm optimization or genetic algorithms [39,40,41], which offer reliable search strategies for “finding parameters that align with reality”. The convergence of these algorithms has been effectively proven under certain conditions. Furthermore, some scholars have integrated this “distance” into the loss function within the deep learning framework, employing gradient-based methods to determine the optimal parameter values for fitting [42].

Regarding (3), it not only acquires model parameters that align with the actual data (by minimizing the distance, etc.), as mentioned in (1)/(2), but also incorporates rigorous mathematical statistical techniques and probability theory interpretation into the parameter inference process, considering the variable distribution within the model. This includes parameter estimation and hypothesis testing. Given the intricacy of dynamic simulation rules (models), statistical strategies employed in this approach often necessitate the utilization of large sample theory, nonparametric estimation, and other methodologies, complemented by Bayesian analysis. In this context, some works either integrate such models with discrete selection frameworks or provide maximum likelihood estimates of parameters based on the assumption of samples and updating rules (such as those related to dynamics like Markov chains) [43]. Another publication introduces an intriguing notion, suggesting an approach in which the initial step entails determining the posterior distribution of parameters using the approximate Bayesian method. Subsequently, the distribution is smoothed through kernel density estimation, ultimately leading to an integrable density function. Finally, parameter estimators and test statistics can be computed using methods like Markov Chain Monte Carlo (MCMC), thus offering a comprehensive framework for analysis and inference [44]. In the forthcoming article, we shall adopt this idea as the foundation to propose a validation method for a class of opinion dynamic models and apply it to real-world data.

Concerning theoretical testing, some scholars apply machine learning methods to segment the dataset from social media into the training set and the testing set, then determine the accuracy of the model on the testing set [42]; if the accuracy is high, it can indicate the reliability of the theoretical model in prediction. In addition, scholars have collected cases of multiple social processes (such as multiple opinion evolution datasets), obtained simulation data through numerical calculations of the model, and verified the consistency between simulation data and case data in probability distribution [5]. In these methods, the researcher only tests whether the model results match the actual results (such as whether the opinion values obtained by simulation are the same as opinion values in the real world), but the consistency test between the “model rules” and the “real rules” is ignored (such as whether the “update method of opinions in the model” matches the “evolutionary rule of opinions in reality”). This is a significant direction worth exploring in our paper for solving problem C.

### 1.3. Literature Summary and Subsequent Research

Based on the above literature review, we have found that there are some problems with the existing solutions to problems A/B/C: (1) For problem A, mechanisms such as in-group cohesion and social exclusion have not yet been introduced into opinion evolution models, resulting in insufficient consideration of their impact on consensus/chaos. (2) For question B, the method of proving synchronization based on dynamical system theory needs further development, which will help to confirm the law between micro-mechanisms and macro-phenomena. (3) For problem C, we still lack effective means to test the consistency between the theoretical model rules and real-world rules. Therefore, we will introduce the mechanism described in (1) in the model of Section 2 (and explore its effects and laws in Section 3 and Section 4), develop the dynamic system method in the proof of theorems in Section 2, and propose solutions to the test problem in (3) in Section 5, thus solving problems A/B/C, respectively, and ultimately helping us answer the core questions 1/2/3 (global consensus/local consensus/chaos) in our research.

## 2. Model and Theorems

### 2.1. Overview

Based on the micro-level mechanisms described in Section 1.1, we attempt to propose a general model for opinion evolution that reflects micro-mechanisms (such as bounded confidence/group pressure illustrated in Section 1.1) and includes models (such as H-K/D-W and social influence models introduced in Section 1.1 and Section 1.2.1) to address Problem A. At the same time, the model also needs to describe the macroscopic outcomes mentioned earlier, such as global consensus/local consensus/chaos. From this, we can derive the correlation between micro-mechanisms and macro-phenomena in the model and obtain multiple theorems to preliminarily solve problem B.

### 2.2. Model

Guided by the above ideas, we establish a dynamical model consisting of the following parts, reflecting the interaction of ordered opinions among individuals (the strict version of our model is presented in Appendix A):

(1) **Environment:** The environment of our model includes a social network G=(V, E), a confidence threshold set $D=\{d_{i}\in \left[0,1\right]:\;i=1\ldots n\}$, a social learning/exclusion rate set $\;U=\{u_{ij}\in R:\;i,j=1\ldots n\}$, and a pressure coefficient set $K=\{k_{i}\in \left[0,1\right]:\;i=1\ldots n\}$. Here, $V=\left\{v_{1}\ldots v_{n}\right\}/E=\left\{e_{ij}\right\}$ in G represents the set of all individuals/the set of all relationships between individuals (individuals can be called agents in the following text). Specifically, v_i_ represents individual i (abbreviated as i), and e_ij_ represents the social relationship between individual i and individual j. d_i_ in D represents the confidence threshold of v_i_ depending on the agent i, whose meaning is the same as the confidence threshold in the D-W model. u_ij_ in U represents the “social learning rate of v_i_ towards v_j_’s opinion”/“the social influence rate of v_j_ on v_i_’s opinion”, that is, “the degree to which v_i_ adopts v_j_’s opinion after interacting with each other”. k_i_ in K represents the influence of group pressure/mainstream opinion on individual i, hence the term “pressure coefficient”.
(2) **Variables:** The variables of our model encompass opinion x_i_(t), social neighborhood N(i), and confidence neighborhood N(i, t), which can be explained respectively. First, Each individual v_i_ has an opinion value at time t, denoted as $x_{i}(t)\in [0,1]$, representing preferences for certain goods or political positions on certain events, etc. Second, each individual v_i_ has a “social neighborhood”, denoted as $N_{i}=\left\{v_{j}:\;e_{ij}\in E\right\}$, which represents all individuals in the social network G who have relationships with v_i_, such as being friends or following each other. Third, each individual v_i_ has a “confidence neighborhood” denoted as $\;\;N_{it}=\left\{v_{j}:\;\left|x_{j}\left(t\right)-x_{i}\left(t\right)\right|<max\left(d_{i},d_{j}\right)\right\}$, which represents all individuals v_j_ whose opinion difference with v_i_ at time t is not greater than the larger of i’s and j’s thresholds. Under the bounded confidence hypothesis, this represents “all agents with whom v_i_ could potentially engage in communication at least unilaterally” (regardless of whether “vi initiates communication with v_j_” or “v_j_ initiates communication with v_i_”; this is because it just needs a single person to start a conversation in most scenes).
(3) **In-group Rule:** At time t, for any individual v_i_, considering all individuals v_j_ in N_i_∩N_it_ (i.e., individuals with whom v_i_ has a social relationship and whose opinion differences can be trusted and accepted by v_i_), a subset $\left\{v_{i1}\ldots v_{ik}\right\}$ is randomly selected from this set to interact and exchange opinions with i, and the selection is denoted as a random event w_t_, with its probability defined in some contents like M1/M2/M3 in the section of Main Theorems and Appendix A. (This is because strictly defining probability is very cumbersome and relies on the measure theory, so it is included in the Appendix A). This subset consists of all individuals who interact with i, written as N(i, t). In this context, we denote the average influence of all v_j_’s opinions on v_i_ as $\left(1/|N\left(i,t\right)|\right)\sum_{j\in N(i,t)}^{}\left(u_{ij}\cdot x_{j}\left(t\right)\right)$, where u_ij_ ∈ U represents the social learning rate defined earlier, and |·| represents the cardinality of a set. Additionally, individual i retains a portion of his original opinion, denoted as $\;[1-\left(1/|N\left(i,t\right)|\right)\cdot \sum_{j\in N(i,t)}^{}u_{ij}]\cdot x_{i}\left(t\right)$, where the coefficient of x_i_(t) is for weight normalization purposes.
(4) **Out-group Rule:** At the same time, at time t, individual i browses information posted by other individuals in the social network G and is influenced by the average opinion $Ex\left(t\right)=\left(1/n\right)\cdot \sum_{v_{i}\in V}^{}x_{i}\left(t\right)$ of the network. The degree of this influence is adjusted by the “difference between individual i’s opinion and the mean opinion in Ni(t)” (referred to as “local discrepancy”), denoted as $\left|x_{i}\left(t\right)-\left(1/|N\left(i,t\right)|\right)\cdot \sum_{j\in N\left(i,t\right)}^{}x_{j}\left(t\right)\right|$. Then, We can denote the environmental noise experienced by individual i at time t as the following equation: $noise_{i}(t+1)=|x_{i}\left(t\right)-\left(1/|N\left(i,t\right)|\right)\cdot \sum_{j\in N\left(i,t\right)}x_{j}\left(t\right)|\cdot (Ex\left(t\right)-\left(1/|N\left(i,t\right)|\right)\cdot \sum_{j\in N\left(i,t\right)}x_{j}\left(t\right))$, representing the adjusted group pressure. The overall effect of this pressure on individual i’s opinion is related to the pressure coefficient and denoted as $k_{i}\cdot noise_{i}(t+1)$.
(5) **The General Equation:** Based on the rules described in (3) and (4), after opinion exchange/random event w_t_ occurs, the opinions of all individuals i evolve from x_i_(t) to x_i_ (t + 1), following the update equation:

$$\;x_{i}\left(t+1\right)=[1-\left(1/|N\left(i,t\right)|\right)\cdot \sum_{j\in N\left(i,t\right)}u_{ij}]\cdot x_{i}\left(t\right)+\left(1/|N\left(i,t\right)|\right)\sum_{j\in N\left(i,t\right)}\left(u_{ij}\cdot x_{j}\left(t\right)\right)+k_{i}\cdot noise_{i}(t+1)$$

In this equation, the environmental noise from (4) is incorporated, and the average influence of various v_j_’s on v_i_ from (3) is considered to capture a richer range of opinion sources.

**Figure 1:**
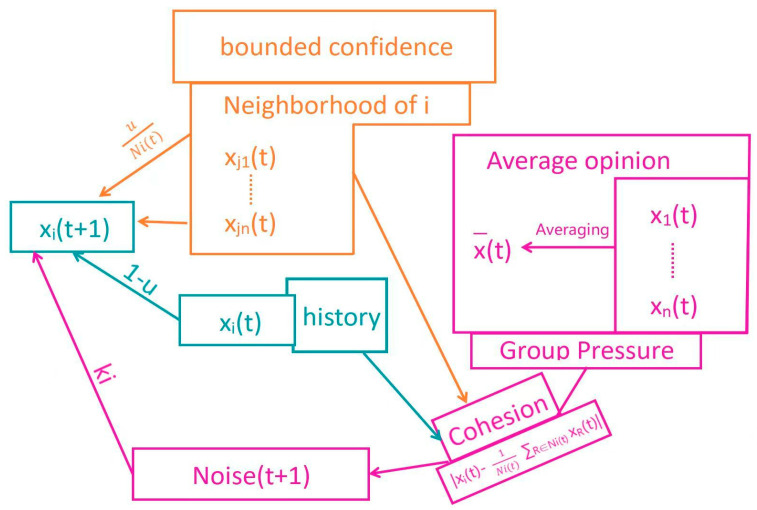
The framework of our model.

Clearly, (1) and (2) describe the environmental setup of the model, which is necessary for characterizing a class of social network dynamics. (3), (4), and (5) represent the evolution rules of the model, whose effectiveness relies on the behavior patterns and ways of interactions among individuals. From this perspective, we can explain how the evolution rules are derived from the micro-level mechanisms described in Section 1.1. The correspondence between this model and the mechanisms can be illustrated in the following Figure 1.

Regarding (3), it integrates the “bounded confidence hypothesis” and the “social influence mechanism”. The “bounded confidence” is reflected in the confidence threshold d, which determines the agents in N(i, t) who have opinion differences within the threshold, and only they can interact with the agent i. “Social influence” refers to the ability to change others’ opinions through persuasion/control, etc. Unlike the parameter u/u_i_ in the homogeneous/heterogeneous H-K/D-W models (see Section 1.2.1), this influencing ability depends on the fixed social network structure (if there is no edge between v_i_ and v_j_, there is no social influence) and has more heterogeneity (the influence of j on i varies for different j), which can be described using Ni/U and u_ij_ in the model and is similar to the strategies 1/4 in Section 1.2.1. This part integrates the “bounded confidence hypothesis” and characterizes social networks/assimilation, and exclusion as different social influence effects, represented by the yellow and blue parts in the diagram.

Regarding (4), noise_i_ (t) is composed of the average opinion Ex(t) of the network and the local discrepancy $\left|x_{i}\left(t\right)-\left(1/|N\left(i,t\right)|\right)\cdot \sum x_{j}\left(t\right)\right|$ of individual i’s opinion. The former measures group pressure, which essentially indicates the pressure and influence of “mainstream opinions” perceived by individuals when browsing extensive information. The latter measures internal cohesion and focuses on the consistency of individual i’s opinion with the “internal group” to which they belong (in this case, the “small group N (i, t)” with which i interacts at time t), which is consistent with the measurement mentioned in Section 1.1. Thus, this part reflects the combination of the “out-group pressure mechanism” and the “in-group cohesion mechanism” based on strategy 3 in Section 1.2.1, represented by the purple part in the diagram.

Based on the above content, we have established a stochastic dynamic framework that integrates bounded confidence and social influence, taking into account different interaction mechanisms involving the in-group and the out-group. Based on this, we can further explain the generality of this model; the D-W/H-K/Degroot models (showing social influence) can all be viewed as special cases of this model. For D-W, it corresponds to the case of our model under the conditions of “G is a complete graph, u_ij_ = u, there exists a unique i and j such that $\left|N\left(i,t\right)\right|=\left|N\left(j,t\right)\right|=1$, and $P\left(i\;interacts\;with\;j\right)=1/\left|V\right|$”. For H-K, it corresponds to the case of “G is a complete graph, $1-u_{i}{}_{j}=1/N_{it}\cdot \sum_{j\in N(i,t)}\left(u_{ij}\right)$, and if $\left|x_{i}\left(t\right)-x_{j}\left(t\right)\right|<d,\;P\left(i\;interacts\;with\;j\right)=1$”. Similarly, the Degroot model is also an example of our model under specific conditions (When G is a complete graph, d_i_ = 1 for all of v_i_ and P(i interact with j) = 1 for any i/j, our model is same with a kind of degroot model (x_i_(t + 1) = ∑w_ij_•x_j_(t)).),which ensures that the theorems established later can be generalized to these types of special cases.

### 2.3. Main Theorems

In response to the model proposed earlier, we establish three fundamental theorems to answer questions 1/2/3, namely the global consensus/local consensus/chaos theorem, serving as preliminary solutions to Problem B. To this end, we first introduce three sub-models of our main model, which aid in stating the theorems. These sub-models have the same form as the main model but need to satisfy different conditions (representing different social environments) and are denoted as M1/M2/M3. We will describe them separately in the following sections. After that, we can intuitively define the macroscopic outcomes (consensus/chaos) in our model and illustrate our theorems.

In M1, there exists an individual i in the social network G who can engage in an opinion exchange with anyone. This requires his confidence threshold to be greater than maxd_i_(0)-mind_i_(0), and there should be edges between v_i_ and any $v_{j}\;(d\left(v_{i}\right)\;=\;n-1$, referred to as “star topology” in the related literature [45]). At each moment, an individual i randomly interacts with only one individual in his neighborhood $N_{i}\cap N_{it}\left(\left|N\left(i,t\right)\right|=1\right)$. Additionally, “the conversation between any i and j” and “the social learning rates” are symmetric (namely, i interacts with j if and only if j interacts with i; u_ij_ = u_ji_ > 0). These conditions indicate that M1 describes an assimilation system with an “active agent” (v_i_), where opinions among two individuals approach each other after the conversation. It is an extension of the heterogeneous D-W and H-K models.

In M2, the social network G is a complete graph Kn, meaning “there is an edge between any i and any j”. The social learning rates and confidence thresholds of all individuals are constants (u_i_ = u, d_i_ = d), and they are not influenced by out-group pressure (k_i_ = 0). Additionally, at each moment, individual i only interacts with one individual in its neighborhood, N_i_∩N_it_, and “the conversation between any i and j” and “the social learning rates” are also symmetric, the same with M1. Clearly, M2 represents an ideal scenario that depicts the evolution of opinions within a localized social group (a relatively closed group with closer and tighter connections among members). Moreover, M2 is a variation of the homogeneous D-W model that allows for multiple pairs of interactions among individuals at any time t, while D-W only allows one pair of interactions at each time step.

In M3, the social network G is only required to be a connected graph, meaning “there is a path between any i and j connecting them”. The confidence threshold d_i_(t) of each individual i is a monotonically increasing function with a limit of 1. Additionally, individual i is similar to M2 and is not influenced by out-group pressure (k_i_ = 0). Clearly, M3 can be understood as an “open society” where a connected graph allows unrestricted propagation of opinions and information among individuals. The increasing confidence threshold represents an expanded range of communication for individuals, and the absence of group pressure indicates freedom of expression of opinions. Furthermore, the social influence model can be viewed as a special case of M3, where d_i_ = 1 for any individual i.

In addition, we also need to clarify the definitions of several types of macroconsequences in the model in order to elucidate our theorems below: (1) Global consensus can be considered as “for any individual v_i_ and v_j_, when t approaches infinity, the probability of $\left|\;x_{i\;}\left(t\right)\;-\;x_{j\;}\left(t\right)\;\right|$ approaching 0 is 1” (strictly speaking, it is called “quasi consensus”), which is determined by the randomness of the above model. (2) Local consensus is equivalent to “the probability of N(consensus) > 1 is 1”, that is, “the probability of x_1_ (t)… x_n_ (t) tending to multiple different values is 1”. (3) Chaos indicates that the probability of x_1_ (t)… x_n_ (t) not converging/and constantly fluctuating is 1.

Under the conditions of these three models and the core definitions presented before, we derive sufficient conditions for achieving global consensus/local consensus/chaos. Based on the above discussion, these conditions also apply to the H-K, D-W, and social influence models (with slight deductions). The formal statements of the theorems are complex and require reference to Appendix A.

**Theorem 1** **(global consensus).** *Under* $\;M1,\left(\left[0,1\right]n,\;\Omega \;,F\right)$ *has stable quasi-consensus*.

**Theorem 2** **(local consensus).** *Under M2, if *u⩽1/2, n=[1/d]+1, $\forall x_{0}\in {\left[0,1\right]}^{n}$, $P\left(w:Nconsensus\left(w,x_{0}\right)⩽n\right)=1$.

**Theorem 3** **(chaos).** *Under M3, if *$\tilde{F}$*is an expanding map, thus*P{w:x(t) has L−Y chaos}=1, *and it will not reach quasi-consensus.*

For the stated theorems, we can provide intuitive sociological explanations: Theorem 1 indicates that in an assimilation system with active agents (M1), opinions will eventually evolve into a global consensus. Theorem 2 suggests that in a relatively closed group with close connections among members and high similarity (M2), the upper bound of the number of opinion categories under local consensus decreases as d increases. Theorem 3 states that in an open society with free information propagation, broad communication, and low group pressure (M3), if there is a certain degree of social exclusion (u_i_ < 0), opinions will evolve into chaos. These explanations can answer questions 1, 2-1, and 3-1 under ideal conditions and demonstrate the significant impact of parameters d_i_, u_i_, and k_i_ on macroscopic results, thus inspiring the subsequent simulation studies.

## 3. Results: Experiment Design and Simulation Analysis

### 3.1. Overview

Based on the model and theorems in the previous section, we attempt to explore the macro-impact of micro-mechanisms (reflected by parameters such as u_ij_) on opinions through simulation in this section to further solve problem B. This is because, compared to previous theorems, simulation can obtain weaker conditions and a wider applicability, which is closer to the real world. Therefore, the section revolves around numerical simulation and is divided into experiment design and results analysis, which will be elaborated in the following parts.

### 3.2. Simulation Experiment Design

According to the ideas in Overview, considering that the aforementioned theorem is only applicable to complete graphs and connected graphs, we perform numerical simulations on a small-world network [46] within our model. This choice ensures that the social relationships represented by the network closely resemble the actual conditions of opinion evolution. Furthermore, we aim to elucidate the influence of various factors, such as network structure, confidence threshold, and social learning rate, on the formation of consensus, to further solve problem B.

To achieve this, we will conduct multiple experiments based on our model. In these experiments, we set up the probability P (i interacts with j) = 0.5 when v_j_ belongs to N_it_∩N_i_ (except for the simulation of M1/M2/M3 to verify the theorems) and focus on the following parameters: (1) the average degree (briefly written as d*) of the network; (2) the social learning rate, denoted as “u_ij_”; (3) the confidence threshold, denoted as “d_i_”; and (4) the group pressure coefficient, denoted as “k_i_”. Moreover, to elucidate the micro-mechanisms impact on macro-level effects, we will first simulate a homogeneous model (with an arbitrary agent i/j, u_ij_ = u, d_i_ = d, and k_i_ = k). Additionally, we will simulate the model under heterogeneity conditions (u_ij_ = u_i_/d_i_ varying upon the agent i). These practices serve to accomplish two fundamental tasks: A. Conducting numerical verification of the aforementioned theorem; B. Investigating the evolutionary mechanisms of consensus/chaos under other broad conditions (both helpful for solving problem B). 

Our simulation environment can be illustrated briefly: First, the number of nodes in the network is 300. Second, the probability of random reconnection is established at 0.3 in the small-world network, and each experiment entails no less than 50 iterations. Furthermore, the model incorporates several parameters as variables, such as u_ij_/d_i_/k_i_, which are manipulated across diverse experiments to ascertain their numerical impact on the outcomes. To facilitate comprehensive analysis, we have devised distinct experimental strategies that can be categorized into two different types mentioned above: the homogeneous model (for any agent i and j, u_ij_ = u, d_i_ = d) and the heterogeneous model (different agents have different values of u_i_ and d_i_). Through simulation, the former unveils multiple ideal mechanisms, whereas the latter shows a substantial departure from ideal mechanisms, thereby enabling a thorough exploration. These experimental strategies are expounded upon below.

To handle task A, we endeavor to validate the consistency between our simulation results and the conclusions derived from Theorems 1–3 by opting for parameter values that satisfy the prescribed conditions. As for task B, we should separately handle the simulation of the homogeneous model and the heterogeneous model, as detailed and illustrated below.

For the homogeneous model, we are in pursuit of comprehending the enduring outcomes of distinct u/d variables and discerning the remarkable influences of k. Therefore, we shall take “the influence of the confidence threshold d on opinion outcomes” as an example to present a comprehensive framework for conducting the control experiment in the subsequent sections of our paper.

(1) Arising from the mechanisms of imitation and exclusion, assign the variable “u” into two categories (denoted as u > 0/u < 0).
(2) Under these two categories of u, analyze the impact of parameter “d” on the evolutionary outcomes of opinions separately. When u > 0, the experimental procedure entails the following steps: A. Control the values of u, d*, and k and observe the simulation results at various values of “d”; B. Conduct multiple experiments using fixed parameter values of d, u, d*, and k; C. Visualize the simulation results, such as the trend of individual opinion values, frequency of global synchronization, and distribution of consensus numbers; Similarly, when u < 0, perform simulation experiments analogous to those conducted when u > 0, such as controlling the same values of d*.
(3) Finally, summarize and compare the obtained results to elucidate the comprehensive influence of “d” on the evolution of opinions under different values of “u”.

In a similar vein, we can apply this experimental protocol (or select particular stages within the protocol based on our needs) to investigate the influence of parameters, such as “d*/k”, on the outcomes of evolution. This endeavor aims to answer questions 1/2/3 about the phenomena of synchronization and chaos.

For the heterogeneous model, our focus lies in examining the interaction between imitators and repulsors (representing individuals i whose u_ij_ = u_i_ > 0/u_ij_ = u_i_ < 0 for all agents j, respectively). This interaction raises a question, stemming from the preceding text: when all agents are imitators (namely, they satisfy u_i_ > 0), if given values of d/d* can engender global or local synchronization, how many repulsors (u_i_ < 0 transformed from u_i_ > 0) can disrupt synchronization and induce chaos? The conclusion drawn from Theorem 3 is confined to the scenario where the network is connected and d is large. However, simulation experiments can further elucidate this question, and we can break it down into two steps:

(1) Set up the imitators/repulsors proportion, denoted as α/1 − α.
(2) Employing the simulation strategy of the homogeneous model, control a set of d/d*/k values that lead to synchronization (when u_i_ > 0) and simulate varying α values to comprehend their impact on consensus formation. Subsequently, we can depict relevant charts based on the results.

Consequently, we can integrate the findings from homogeneity/heterogeneity experiments to investigate the mechanism’s response to “global consensus”, “local consensus”, and the “chaotic state” as mentioned in the introduction (questions 1, 2, and 3 in Section 1.1, respectively). Moreover, the subsequent text will present simulation results categorized according to this classification (questions 1/2/3), accompanied by corresponding explanations and conclusions.

### 3.3. Simulation Results Analysis

#### 3.3.1. Global Consensus

**Figure 2:**
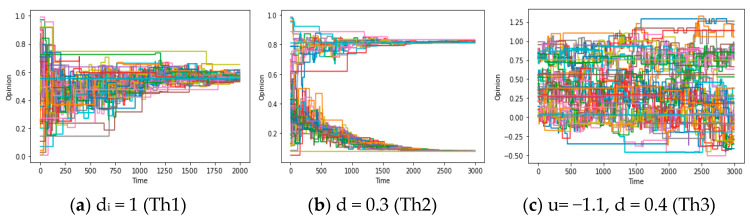
(**a**–**c**) Opinion-Time Chart, social learning rate u_i_ = u = 0.5; pressure coefficient k_i_ = k = 0.1; (**a**–**c**) are simulated on the complete graph/complete graph/connected graph, respectively. Note: The opinion time chart refers to the horizontal axis reflecting time/the vertical axis reflecting opinion values.

In this part, we expect to provide some analysis conducive to answering question 1 (in the Discussion), which requires us to search for the numerical conditions of global consensus arising from the theorems proved before. Therefore, it first involves the verification of these theorems: utilizing the aforementioned approach, we initially simulate the evolution of opinions under the conditions outlined in the preceding Theorems 1–3. As depicted in Figure 2a–c, the simulation shows the formation processes of global consensus, local consensus, and chaos. These simulation results substantiate the reliability of theorems to a certain extent. 

Additionally, to considering the theorems and the experiment design, our focus shifts towards comprehensively elucidating the influence of some parameters on opinion dynamics, especially the impact of the network’s average degree (d*)/the confidence threshold (d)/social learning rate (u) and group pressure coefficient (k) on opinion formation. In this context, we proceed to present an intricate analysis of the simulation results of these four parameters while simultaneously exploring their correlation with the synchronization patterns exhibited by our model. In the Discussion section, we will use these analyses and conclusions to answer question 1 systematically.

**Figure 3:**
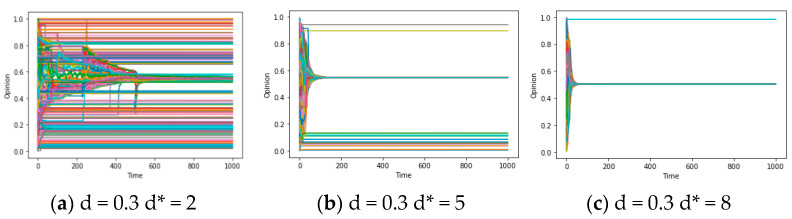

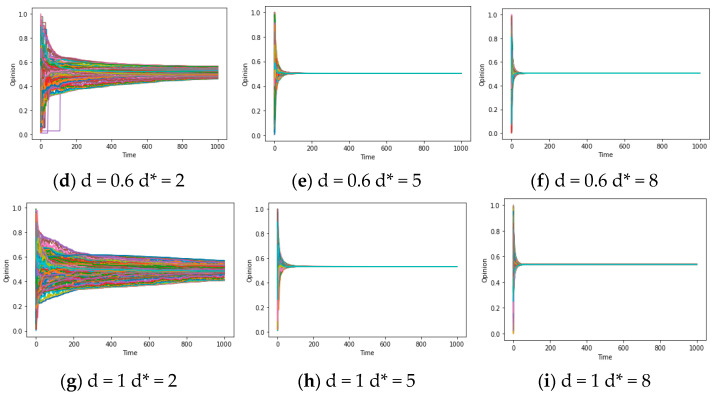
Opinion-Time Chart (**a**–**i**) (social learning rate u_i_ = u = 0.5/pressure coefficient k_i_ = k = 0.1).

In terms of network structure, specified as the average degree d*, the concept at hand pertains to “social reachability”, denoting “the possibility of contact between agents i and j about sharing opinions and information”. In a small-world network characterized by a high average degree, nodes possess a greater number of adjacent nodes, thus exhibiting high “social reachability”. Consequently, employing the aforementioned experimental approach, we control u = 0.4/k = 0.1/d = 0.3 (and d = 0.6) to observe the simulation results across different average degrees (d* = 2/5/8). Figure 3a–i illustrate the dual effects of the average degree d* on the model: (1) with a high average degree d*, few categories of local consensus emerge after multiple simulations, potentially leading to global consensus (as depicted in Figure 3a–c); (2) when d is large, an increase in the average degree d* can accelerate model convergence and facilitate the attainment of global consensus (as demonstrated in Figure 3d–i). In essence, both of these phenomena can be ascribed to a fundamental conclusion: the opinion system can achieve global consensus within a limited timeframe, contingent upon relatively elevated social reachability (a large d*).

Regarding the confidence threshold d, we relax the conditions specified in theorem 2 for the complete graph, obtaining different results from setting d = 0.3/0.6/1 while controlling u = 0.4/k = 0.1 and d* varying between 2/5/8. Upon examining the comparison between Figure 3a and Figure 3d (Figure 3b and Figure 3e), as well as Figure 3d and Figure 3f (Figure 3e and Figure 3g), it becomes apparent that an increase in d from 0.3 to 0.6 triggers a dramatic transformation in the final state of opinion evolution. The transition includes a shift from multiple types of local consensus to global consensus. However, when d escalates from 0.6 to 1, the discrepancies between the final states become less pronounced. This particular characteristic warrants further investigation in subsequent discussions. In general, the increase in threshold d brings out the emergence of global consensus or results in a smaller number of local consensus categories. This attribute represented by threshold d may be referred to as “confidence reachability”, signifying “the likelihood that agents i and j of the system are willing to engage in subsequent interactions (In this article, contact and interaction have different meanings. The former refers to an individual’s exposure to information from others, while the latter refers to an individual’s willingness to communicate and update his opinion with others after exposure to information) following their initial contact”.

In addition, it is imperative to consider the influence of the social learning rate (u) and the group pressure coefficient (k). Before the discussion, we can designate the effects of u and k as the assimilation effect (or exclusion effect when u < 0) and homogenization effect, respectively. The former represents the mutual approach of opinions after interactions between people (such as agent i and agent j), while the latter stands for the tendency of opinions to the mainstream opinion (shown as the average opinion) arising from group pressure. Specifically, assimilation refers to the effects of strategies such as mutual imitation/persuasion in communication (taking agent i as an example, its information source is agents interacting with i), while homogenization refers to individuals’ identification with the opinions of the majority due to group pressure (agent i’s information source is all individuals in the social network). Below are detailed elucidations for these two effects based on simulation results for parameters u and k.

**Figure 4:**
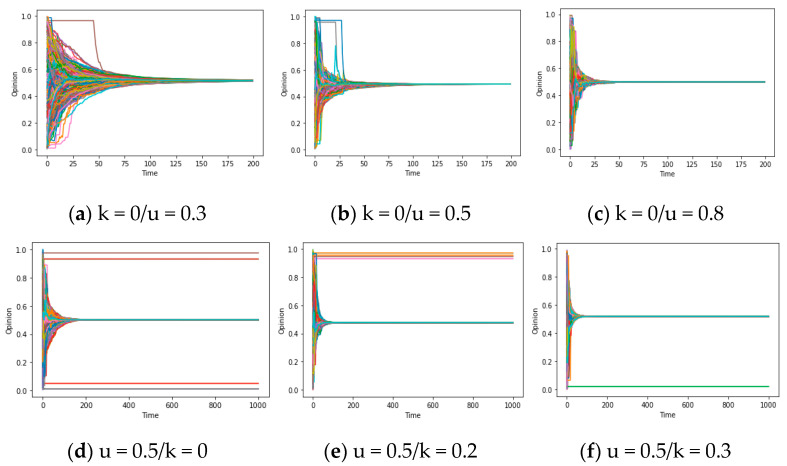
(**a**–**f**) Opinion-Time Chart (d = 0.4/d* = 5).

For the parameter u, in accordance with the results provided in Figure 4a–c, the process of consensus formation accelerates when the variables d/d* satisfy the synchronization condition and u ascends from 0.2 to 0.5/0.8. In other words, when u surpasses 0, the increase in u plays a significant role in improving the assimilation effect and speeding up the emergence of global consensus. This is because from Figure 4a–c, the time for different individuals’ opinions to converge on consensus is significantly reduced (while u is increasing). Conversely, if u < 0, this assimilation effect will be transformed into an exclusive effect, thereby rendering it inapplicable within the above framework. This phenomenon will be discussed later in the “Chaos” section. For the parameter k, according to the results depicted in Figure 4d–f, the value of the group pressure coefficient k exhibits a positive correlation with the possibility of synchronization. This correlation can be attributed to the fact that the presence of noise (i, t) inclines towards transforming divergent individual opinions into the average opinion (although the small values of d/d * limit communication between some individuals, such as the upper and lower opinions in Figure 4a, the higher k allows each individual to consider the average opinion of everyone in the network), resulting in the consensus, which is called the homogenization effect above. The amplification of noise serves to convert local consensus into global consensus, which will be further expounded upon in subsequent sections. On the whole, the attributes of u and k encapsulate an additional imperative component that engenders global consensus: the assimilation and homogenization within the opinion system.

**Figure 5:**
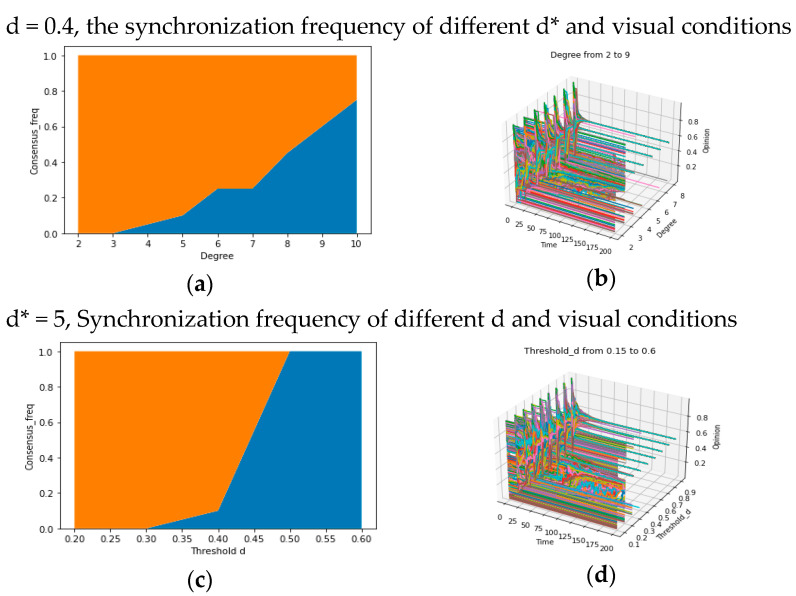
(**a**–**d**) Frequency of global consensus (or “synchronization”) reached by the model under different d*/d.

In the above paragraphs, we preliminarily explored the influence of parameters d/d*/u/k on synchronization. In order to depict the formation of global consensus in different micro-conditions more quantitatively, we conducted additional experiments to verify the macroscopic impacts of the two core parameters aforementioned, which have been known to have distinct roles, respectively: the average degree d* and the confidence threshold d, representing different “reachability”. Our experiments are comprised of three parts: (1) regarding social reachability d*, we conduct 20 simulation experiments on systems with varying average degrees (d* = 2…10) while keeping d = 0.4, and the frequencies of attaining global consensus in experiments (freq = number of global consensus/20) are recorded, yielding Figure 5a; (2) for confidence reachability, we set d* = 5 and recorded the frequencies of achieving global consensus for d values ranging from 0.3 to 0.6. The corresponding results are illustrated in Figure 5d; and (3) for both social reachability and confidence reachability, we draw the 3-dimensional graphs to show the evolution of opinions under different values of parameters d/d*, presented in Figure 5b,d. 

From the results obtained from the experiment elaborated above, we find the synchronization frequency (the frequency to achieve global consensus) is positively correlated with d and d* (Figure 5a,c), and the number of opinion categories is decreasing when the value of d (or d*) is increasing (Figure 5b,d). These results are consistent with the analysis in the above paragraphs and are more reliable because of the comparison between more possible results under many values of d and d*.

In summary, when u > 0, if the values of the confidence threshold d and the average d* are large, the opinion tends to reach global consensus. In this mechanism, as the value of u increases, the rate of reaching global consensus will become faster (assimilation effect). If the value of the confidence threshold d and the average degree d* are not large enough, a larger group pressure coefficient k can convert local consensus into global consensus (homogenization effect). These conclusions will be further explained in the Discussion section, and a general answer to question 1 will be provided based on the concept of reachability defined above.

#### 3.3.2. Local Consensus

In this part, we expect to provide some analysis conducive to answering question 2 (in the Discussion), which requires us to search for the numerical conditions of local consensus. Based on the mechanism obtained by the above results, we recognize the effect of u/d/d*/k on consensus, which can be briefly summarized as assimilation (u), reachability (d and d*), and homogenization (k). Among the three effects, u mainly affects the final result of opinion formation through symbols, and k is not necessary for global consensus (such as k = 0 in Figure 3a); only the absolute values of d and d* play a crucial role in consensus states (global or local). Therefore, we can focus on exploring the effects of d and d * in this section to answer question 2 (local consensus) and probe the influence of k after this exploration.

For the purpose of understanding the relationship between d/d* and local consensus, we can still examine the pertinent experimental results shown in Figure 5a–d in the previous part. It indicates that when given d = 0.4 and d* = 5, both d* and d exhibit minimal impact on the frequency of synchronization as they decrease from higher values (e.g., 0.6/7) to 0.5/6. However, when they decrease further from 0.5/6, the synchronization frequency becomes more sensitive to these changes. Therefore, we hypothesize that beyond a specific threshold for d or d*, multiple “bifurcation points” emerge (a threshold), which may result in a “truncation phenomenon”: When the parameter value surpasses this threshold, the system may only achieve n kinds of local consensus (where n = 1 represents the global consensus). Conversely, when the value falls below this threshold, it has n + 1 kinds of local consensus, just like a truncation of the consensus formation process, which is regarded as a bifurcation in the field of dynamical systems. Specifically, when an individual’s d * or d is less than the ‘bifurcation point’, they are unable to effectively assimilate individuals with significant differences in their own opinions/social distances (xi (t) and xj (t) with significant differences cannot approach each other), resulting in the inability to further narrow down opinions with significant differences. This supposition forms the crux of answering questions 2-1 and 2-2. Hence, we will employ diverse parameter combinations of d/d* in subsequent analyses to experimentally investigate the number of local consensuses.

**Figure 6:**
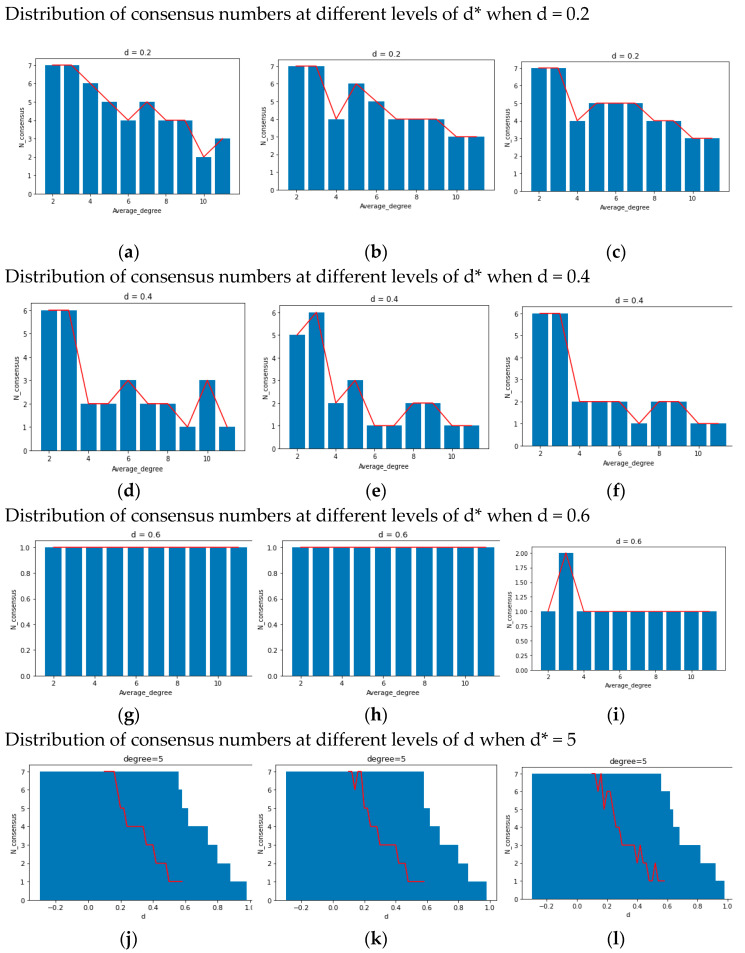
(**a**–**l**) Distribution of Consensus Numbers for Given d/d*, d*/d.

To investigate the “bifurcation points” and truncation properties of d and d* separately, we meticulously control each of the two parameters in isolation. The specific methodology is elucidated in the figure annotations below. Based on Figure 6a–i, under a fixed d value, the system exhibits remarkable stability across varying d*. However, the count of consensus occurrences still exhibits a dependency on the network average degree. In contrast, as illustrated in Figure 6j–l, with a fixed d*, the system demonstrates a pronounced sensitivity to perturbations in d. Even a modest change of 0.2 can yield significant disparities in the number of consensus categories, such as the conspicuous transition from a global consensus to 2~3 kinds of local consensuses around d = 0.5. Generally speaking, if the value of d(or d*) is fixed, the other parameter d*(or d) will have some bifurcation points transforming n kinds of consensus into n + 1 kinds of consensus.

**Figure 7:**
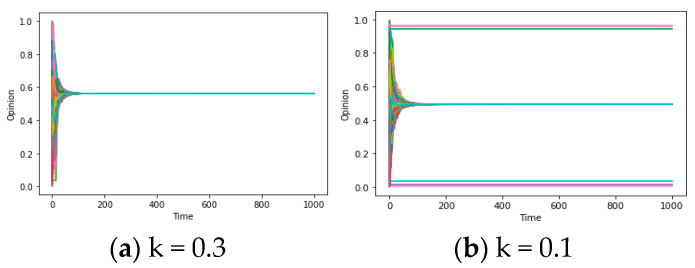
(**a**,**b**) Opinion-Time Chart (u = 0.5, d* = 5, D = 0.4).

After elucidating the primary causal factor, namely “truncation” from the values of d and d*, contributing to the emergence of “local consensus, “we can delve into the meditative effect from group pressure coefficient k to the opinion formation outcomes. We control the values of u/d/d* and vary the k, to obtain the simulation results visualized in Figure 7a,b. These figures offer an intuitive depiction of such characteristics: while a global consensus is observed when the parameter combination of (u, d, d*) is set as k = 0.3, reducing the group pressure coefficient (k = 0.1) can lead to an increase in the number of consensus instances, amounting to three, thereby emerging as a crucial property within the model. This is the homogenization mentioned earlier, and it means that k can help the system reach global consensus when it may lead to local consensus with no homogenization effect (k = 0), and vice versa.

Overall, when u > 0 and values of d/d* representing confidence/social reachability are decreasing in the opinion system, it may truncate the original consensus formation, giving rise to more kinds of local consensus. Additionally, a small value of k can retain the local consensus, hindering it from changing into the global consensus.

#### 3.3.3. Chaos Phenomenon

After obtaining some results about questions 1 and 2, we will explore the chaos described in questions 3-1 and 3-2 in this section and deeply excavate them in the Discussion. For question 3, theorem 3 states that under the restriction of a connected graph, if F is an expanding map (showing that people’s opinions are mutually exclusive and distant in the communication), opinions will exhibit chaos, and consensus becomes unachievable. Simulation results further support this conclusion. Figure 6a demonstrates that when the assimilation mechanism shifts towards exclusion (from u > 0 to u < 0) while maintaining strong social and confidence reachability, the system exhibits chaos within a specific range. 

**Figure 8:**
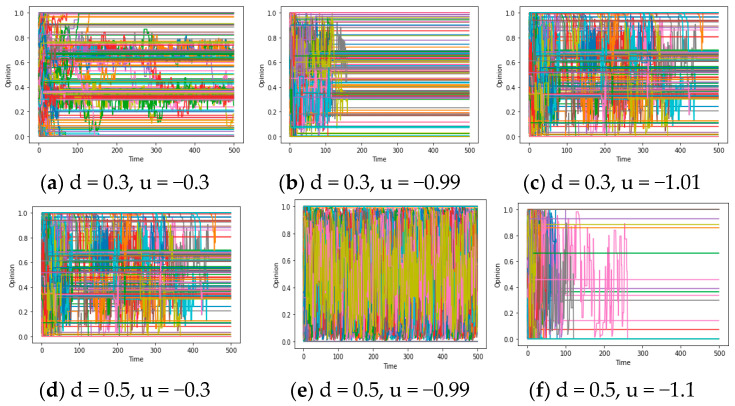
(**a**–**f**) Opinion-Time chart (d* = 5/k = 0.1).

To investigate this phenomenon further, we can set the experimental parameters u to −0.3, −0.99, and 1.1, respectively, while controlling the other parameters. It is worth noting that we need to set sufficient reachability (d/d*) to enable effective communication between individuals. By comparing Figure 8a–f, we observe that for −1 < u < 0, the chaotic region expands as the absolute value of u increases (in the context of relatively large d/d*). In other words, when reachability is high, a larger absolute value of u can cause more people’s opinions to enter complex and repetitive fluctuations. This may illustrate the positive correlation between u (the social exclusion rate) and the level of chaos. 

**Figure 9:**
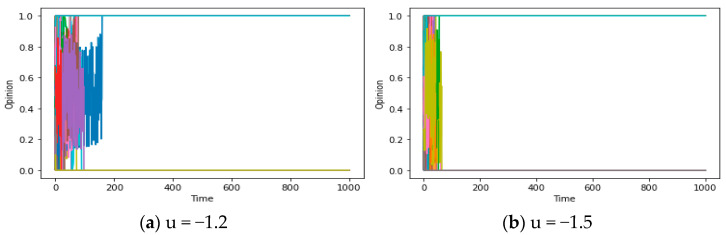
(**a**,**b**) Opinion-Time Graph (d = 0.6 d* = 8 k = 0.1).

However, in the case where u is less than −1 and deviates significantly (e.g., −1.2), an intriguing phenomenon may arise within the system, known as “polarization”, as depicted in Figure 9a,b. Due to the bounded nature of opinions, a peculiar “pseudo convergence” manifests, setting it apart from the local consensus of N (consensus) = 2 discussed before. This distinction is apparent: when the number of local consensus categories is two, the constrained reachability (d/d*) results in the global consensus formation process being truncated, ultimately reaching a stable and convergent state. Meanwhile, the magnitude of the final opinion range is evidently smaller than that of the initial values, indicating a propensity towards assimilation and homogenization of individual opinions (namely, maxx_i_(t)-minx_i_(t) < maxx_i_(0)-minx_i_(0)). But in the figures below, the “social exclusion” mechanism (u < −1) engenders a growing disparity among individuals with divergent opinions, leading to an expanding range of opinions over time, culminating in convergence values situated at the upper and lower bounds of the opinion spectrum. This may serve as a concise explanation for the polarization of opinions in reality, which needs to be discussed in the following section.

**Figure 10:**
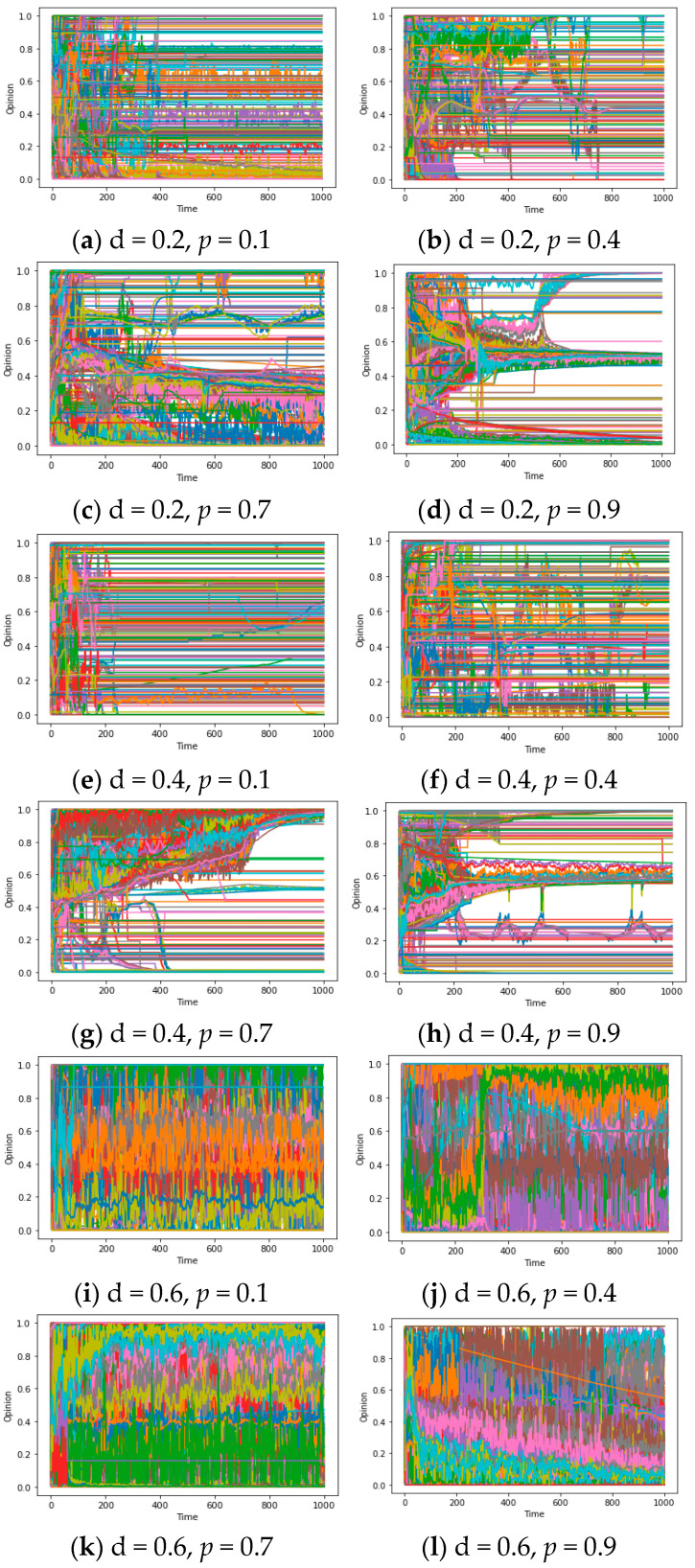
(**a**–**l**) Opinion-Time chart (probability of u_i_ being a positive number is *p*, d* = 5, k = 0.1).

Finally, for the sake of ensuring consistency between our model and the real world, it becomes imperative to utilize the heterogeneous dynamic models mentioned in the experiment design to simulate opinion evolution. In order to explicate evolutionary patterns of the opinion system with multiple u_i_/d_i_, we set different values of *p* (representing the ratio of repulsors whose u_i_ < 0) to apprehend the influence of disparate u_i_/d_i_ distributions on synchronization. The outcomes are elucidated in Figure 10a–l. It is noteworthy that irrespective of whether the value of d is 0.2/0.4 or 0.6, the presence of “repulsors” with a ratio of 0.1 in the system (i.e., u_i_ < 0) significantly disrupts the convergence of the model, resulting in the phenomenon of “chaos”. According to subsequent investigations, this characteristic persists even when the proportion of repulsors is 0.01, which may suggest a small perturbation to assimilation that also possesses the capability to entirely subvert the existing (local/global) synchronization mechanism.

To sum up, in the homogeneous model, repulsors with u < 0 and a relatively high level of reachability will cause chaos, and the area of opinion chaos is positively correlated with the absolute value of u within a certain range. However, when u < 0 and deviates from −1, the system may experience polarization. In heterogeneous models, a very small proportion (such as *p* = 0.01) of repulsors (u_i_ < 0) can disrupt assimilation and generate chaos. These results are worth integrating with the previous results in the discussion to form a theoretical answer to question 3.

## 4. Discussions: Ideal Synchronization Theory

### 4.1. Overview

Drawing upon the conclusions from theorems and simulation results (used for solving problem B), we are capable of establishing an integrative explanation for questions 1/2/3, named after ‘ideal synchronization theory’, which is also a general solution to problem B. The theory can be divided into the main part and the supplementary conclusions: the former focuses on providing sufficient conditions for consensus and chaos based on micro-mechanisms like assimilation and reachability (in the ideal context), as a direct answer to our questions; the latter exhibits some factors facilitating or hindering the consensus formation, whose context is more similar to the real world (such as the heterogeneous u_i_). We’ll first illustrate the main part of our theory below, which is comprised of discussions about global consensus, local consensus, and chaos, respectively.

### 4.2. Main Part

As for global consensus (question 1), two conditions should be taken into consideration, which are ‘assimilation’ and ‘reachability’: (1) In regard to assimilation, from the theorem 1 in Section 2.3 and the simulation result shown by Figure 2, Figure 3, Figure 5 and Figure 8 in Section 3.3.1 and Section 3.3.3, we regard the feature (u > 0) as essential for synchronization since few repulsors can turn consensus into chaos (which is not a convergent state). However, the level of assimilation (the absolute value of u) may just influence the rate of convergence, which does not have strict restrictions. (2) With respect to reachability, it encompasses “Social reachability” and “confidence reachability”, respectively representing the possibility of “contact” (access to other people’s information) and “interaction” (communicating with other people for updating the opinion). Based on the results in Figure 2, a high level of reachability (value of d/d*) can facilitate an effective interaction among agents, ensuring the outcome of global consensus. Specifically, when there is assimilation between agents, agents can first contact enough other people’s opinions through high social reachability (d* represents the number of friends they can reach), and then because of high confidence reachability, after the contact, they can communicate with more people who have large opinions’ differences with them (d represents acceptable differences in opinions), resulting in mutual proximity of their opinions. This process will continue to repeat itself over a long period of time to achieve consistency of opinion among a large number of individuals and promote consensus. In brief, the answer to question 1 is “assimilation mechanism” and “high reachability” (both social reachability and confidence reachability). This answer will be noted as A1 below.

Concerning the local consensus (question 2), we should respectively reply to questions 2-1/2-2. For 2-1, the assimilation feature (u > 0) is still vital for local consensus arising from its capacity for convergence, which is the same reason for the discussion on global consensus. However, according to the results presented by Theorem 2 in Section 2.3 and Figure 6 in Section 3.3.2, one of low reachability (small values of d or d*) should keep in the opinion evolution to sustain the divergent kinds of local consensus. Therefore, the answer to 2-1 is “assimilation mechanism” and “low reachability” (social reachability or confidence reachability). This answer will be noted as A2 below.

And for 2-2, we discover a phenomenon called “truncation” in Figure 6 in Section 3.3.2, which represents the process from local consensus to global consensus being “truncated”. Generated by the reachability from high level to low level, the phenomena reflect the contact block (low social reachability) and the interaction block (low confidence reachability) among people in different areas of the social network, which is one of the core mechanisms hindering local consensus from global consensus. In other words, when the reachability of most individuals is insufficient, they only communicate with people within their social neighborhood (d*) or those with similar opinions (d), resulting in opinion clusters and triggering local consensus. Moreover, from Figure 7 in Section 3.3.2, we can elucidate the correlation between the homogenization effect (pressure coefficient k) and local consensus: if the former is small, it can be a “maintenance mechanism” for the latter. The small homogenization effect (small value of k) encompasses two distinct aspects: (1) during the initial stages of opinion formation (before convergence), it lacks the requisite resistance against the process of “truncation” caused by low reachability; (2) during the later stages of opinion formation (after convergence), it serves as a “sustaining condition” for the enduring existence of local consensus. We will discuss these two points separately in the following text:

(1) Regarding the first aspect, when the value of k is small, even if the noise (i, j, t) is substantial (indicating pronounced out-group pressure/low in-group cohesion), the pressure perceived by individual i, denoted as k*noi(i, j, t), remains insufficient to align his opinion with the global average opinion. Therefore, in the early stages of opinion evolution, under the limitations of low reachability (truncation), individuals are unable to communicate with a sufficient number of people, and due to the low-pressure coefficient k, they cannot fully adopt the majority opinion or the average opinion in the network. This results in a large number of individuals only assimilating with people within their social neighborhood or people who are similar to their own opinions (d/d * limitation) for a long time, causing multiple local consensuses within the system.
(2) As for the second aspect, due to the regulation of cohesion (approaching | xi xj | ≈ 0), group pressure perceived by people is too small to foster consensus formation, thus upholding the prevailing local consensus. This is consistent with the principle of the first aspect, which states that individuals do not consider the average opinion (representing the majority) when there are few people communicating, but the reason for it is that due to the enhanced in-group cohesion, individuals fall into group blindness and no longer accept people from the out-group.
(3) These two aspects can be termed the “cocoon room mechanism”, encapsulating the shielding effect of external information and the external group influence. In conclusion, the answer to questions 2-2 includes the “truncation” caused by low reachability and the “cocoon room mechanism” (high cocoon room effect means low homogenization represented by pressure coefficient k). This answer will be noted as A2* below.

In regard to chaos (question 3), questions 3-1 and 3-2 should be answered as a whole: From Figure 8 and the analysis of these results, a sufficient level of reachability (d) and the exclusion effect (u < 0) are core conditions leading to the opinion chaos, which constitutes the answer to question 3-1. Meanwhile, this condition is intertwined with a typical mechanism: When individuals exhibit a rejection attitude towards the opinions of others, the contact/interaction facilitated by social/confidence reachability can cause people’s opinions to become distant from each other, leading to opinion chaos, which can be named an “exclusion mechanism”. This is because when individuals are mutually exclusive (such as in arguments), the opinions of the communicator will gradually become distant and unable to converge. At the same time, due to the large number of individuals in the system, their opinions will repeatedly change due to the exclusion of others (such as turning right due to the exclusion of the left-wing people in the previous moment/turning left due to the exclusion of the right-wing people in the later moment), ultimately resulting in complex fluctuations. Generally speaking, the conditions of social exclusion (u < 0)/high reachability (large d and d*) and the “exclusion mechanism” can answer questions 3-1 and 3-2, respectively. This answer will be noted as A3/A3* below.

Based on the aforementioned conclusions, we can synthesize the answers to questions 1/2/3 (A1/A2/A2*/A3/A3*) and provide a coherent conceptual framework constituting the core contents of “ideal synchronization theory”. The framework encompasses three fundamental dimensions, namely “assimilation-exclusion” (designated as A-E), “high reachability-low reachability” (designated as H-L), and “the level of cocoon house effect” (designated as the level of C). Concerning A-E and H-L, the ensuing deductions can be drawn: when the system has the features of A and H, the global consensus can be achieved (A1); when it has the features of A and L, it engenders a local consensus (A2); when it contains the features of E and H, the emergence of chaos becomes feasible (A3 and A3*); when it consists of E and L, the system manifests more intricate and incongruous phenomena (which do not belong to the questions of our paper). As for C, if the level of C is low, the system can transform local consensus into global consensus by preventing truncation phenomena, but if the level of C is high, it is not capable of doing so (A2*). These conclusions can be effectively summarized in the Table 1 below: 

### 4.3. Supplementary Conclusions

In addition to the main framework mentioned above, the ideal synchronization theory also includes some supplementary conclusions that are closer to the real world (and therefore deviate from the ideal scenario). These conclusions are still related to global consensus/local consensus/chaos, so they can be discussed separately from these three aspects.

As far as the global consensus is concerned, we find that according to Figure 4 and its analysis, when the level of assimilation is high (the social learning rate u is high), the rate of convergence of opinions in the system is fast. This conclusion is essential: since the evolution of opinions in the real world is limited by attention to specific issues and often only includes limited updates, the rate of convergence will determine whether consensus can be reached at last. In the general model proposed in our paper, this conclusion depends on subsequent proof.

In terms of local consensus, we find that according to Figure 6 and its analysis, both “social reachability d*” and “confidence reachability d” have bifurcation points, and small perturbations around this point can change the final outcome of opinion evolution. Therefore, we need to deeply study the bifurcation theory of stochastic dynamical systems and apply the pertinent results to the study of this problem.

As far as chaos is concerned, we have made two interesting discoveries: (1) According to Figure 9, in the homogeneous model, when the absolute value of the social exclusion rate is too large (for example, u = −1.2), the opinions will eventually become polarized and lie at the upper and lower bounds of the value range, which may explain the political polarization, which shows the “pseudo convergence” aforementioned; (2) According to Figure 10, in the heterogeneous model, a small proportion of repulsors can destroy consensus and lead to chaos, which may explain the destructive effect of some social robots and the “troll” (with exclusiveness) on public discussion.

In summary, the above conclusions are novel discoveries brought about by the general model proposed in our paper. Although they have not yet been rigorously theorized, they can demonstrate the explanatory power of the model and are expected to be further explored.

## 5. Validation: Verifying the Theory with Real Data

### 5.1. Overview

In the above, we have established a general model to describe the evolution of ordered opinions (solving problem A). Through mathematical proof and simulation, we obtain the microscopic conditions leading to consensus/chaos (solving problem B), thus providing answers to questions 1/2/3. However, the above conclusions are theoretical and have not yet established a connection with the real world. Therefore, we should transition to solving problem C in this section, which requires us to estimate and test the model through real data (from social media) in order to verify the reliability of the model and ideal synchronization theory. It is worth noting that we will adopt the model conditions in simulation to carry out our validation, which satisfies “u_ij_ = u” (the homogeneous model) or “u_ij_ = u_i_” (the heterogeneous model) for any agent i/agent j, and the probability P (i interacts with j) = 0.5 when j belongs to N_it_∩N_i_, but the network is directly from our data and not a small-world network in the simulation.

### 5.2. Data Description

The dataset utilized in this article is sourced from the Harvard Dataverse, which served as the empirical foundation for the study titled “Opinion Dynamics of Online Social Network Users: A Micro Level Analysis” [47]. This dataset, collected from VKontakte, the preeminent social media platform in Russia, can be delineated into two fundamental components: (1) the network of friendships among users on the platform, and (2) the corresponding opinion values of users three times. Concerning the former, the dataset provider employed the networkx library in Python to extract the largest connected component within the network, which forms the central focus of our research. About the latter, the opinion values were generated through a collaborative process involving the annotation of texts and semi-supervised learning techniques. These values can be categorized into five distinct political stances, delineated by the intervals [0, 0.2)…[0.8, 1], thereby representing a continuum ranging from strong liberals to strong conservatives in ideologies. Consequently, this dataset serves as a valuable resource for subsequent statistical inferences and offers substantial support for our study.

### 5.3. Inference Methods

#### 5.3.1. Validation and Estimation

To validate our opinion model based on the data mentioned above, we should use a reliable and universally applicable statistical method. In this regard, we integrate the Bayesian approach with non-parametric estimation to derive a comprehensive framework. The overall procedure is as follows [37]: (1) employing approximate Bayesian computation (ABC), we generate a posterior distribution sample of parameters (u_i_/d_i_/k_i_) by leveraging both real data and model simulation outcomes; (2) utilizing kernel density estimation, we smooth the empirical distribution of parameters into a posterior density function; and (3) employing Markov Chain Monte Carlo (MCMC) [48] and other techniques, we compute various integrals of the density function to obtain posterior estimates and test results for the parameters, thereby accomplishing the inference task. The detailed methods are presented in Appendix C.

#### 5.3.2. Fitting and Testing

Based on the aforementioned estimation method, we can utilize the data to acquire the values of u_i_/d_i_/k_i_ and subsequently generate multiple simulation samples. In order to test whether the estimated model is sufficiently consistent with the real data (thereby reflecting the ability of the theoretical framework to match real-world phenomena), we initially compute indicators such as the accuracy of the simulation results. This serves as a preliminary examination to determine the alignment between the simulated data and the social media data.

Simultaneously, to further establish the adequacy of the “simulation model” in comparison to the “real model”, we can approach it by assessing the congruity between the simulation rule F and the real evolution rule T in terms of their distributions (mentioned in Section 1.3 as a difficult problem). For this purpose, we have presented an exploratory methodology, shown as follows: (1) Assuming that the genuine evolutionary rule T can be characterized as a stochastic linear operator [49], then F and T (Due to the fact that in the model, individual i randomly selects others to engage in conversations (which is consistent with the randomness of daily communication), we consider F and T as two stochastic rules) can be viewed as two finite-dimensional matrices under the event wt (namely, F(w) and T(w) are two matrices and also two n^2^-dimensional random vectors); (2) Employing numerical simulation, sampling T and F to obtain two sets of random samples denoted as F(w) and T(w). (3) By utilizing the extended Cramé-von Mises metric [50], we determine whether these two samples are in the same distribution. The detailed procedures for these specific methods are also provided in Appendix C.

### 5.4. Results and Analysis

#### 5.4.1. Parameter Estimation and Fitting

Based on the aforementioned methodology, we employed the network data and opinion data discussed earlier to estimate the parameters. In order to strike a balance between interpretability and predictability, we conducted estimations for both homogeneous and heterogeneous models. The former solely incorporates three parameters, namely u/d/k, which offer insights into the overall magnitude of the social learning rate, confidence threshold, and group pressure in the real world. The latter entails 3 * |V| parameters, enabling a closer alignment with the underlying reality and serving as a reliable predictor of opinion trends. The subsequent table provides the estimated values for the models, along with an assessment of the discrepancy between the predicted and actual data resulting from this parameter estimation.

**Figure 11:**
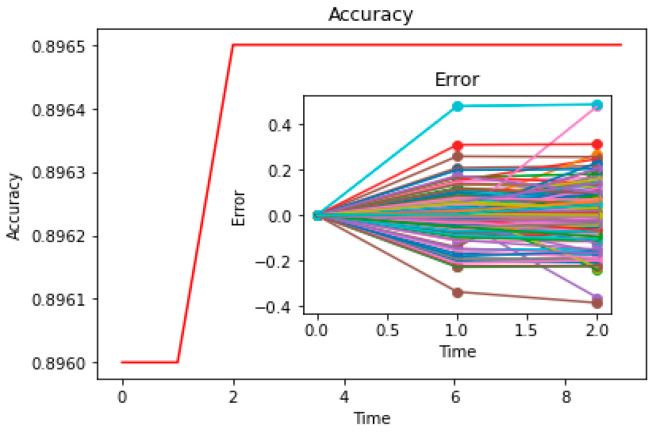
The following image reflects the accuracy of the model in predicting five political positions as well as specific numerical deviations.

From the analysis of Table 2 and Figure 11, we obtain a remarkable finding. Even in the case of a homogeneous model encompassing only three parameters, it exhibits superior performance in fitting the real data. The accuracy of political stance prediction almost reaches an impressive 0.9, with virtually no predicted values deviating from the actual values by more than 0.2. This observation, supported by Bayesian factors derived from Table 2 and parameters of the homogeneous model, highlights a key insight regarding the dataset under examination in this study: The social learning rate (u) appears to be approximately twice the group pressure coefficient (k), indicating the fundamental role played by the “bounded confidence mechanism” in opinion evolution. Furthermore, the confidence threshold for individual interactions hovers around 0.2/0.1, suggesting that, on average, individuals primarily engage with others who share a similar political stance. In conclusion, these results collectively endorse the integration of the bounded confidence mechanism, the group pressure mechanism, and cohesion within the model as a robust framework for generating existing data, thereby empirically affirming the effectiveness of previous theoretical analyses.

#### 5.4.2. Distribution Test

**Figure 12:**
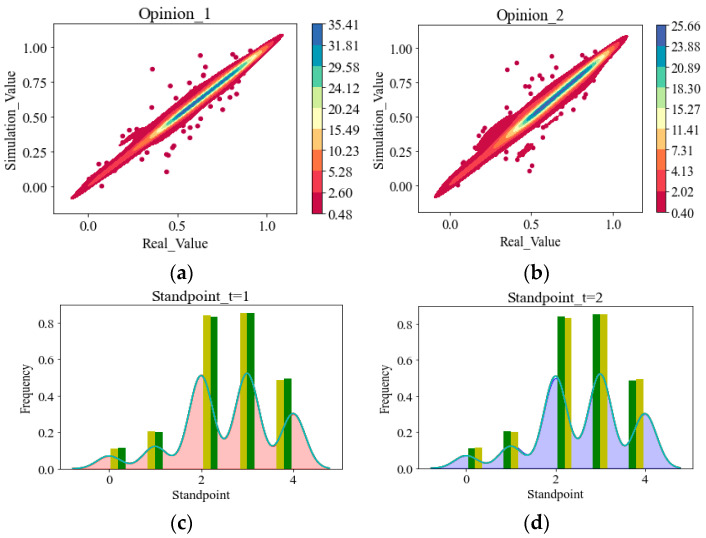
(1) (**a**,**b**) represent the scatter plots drawn by the data points of (real_openion, simulation_opinion). (2) (**c**,**d**) show the frequency distribution histograms of the real opinions (yellow) and the simulated opinions (green) at different times, respectively.

Although the congruity between the simulation model and actual data has been validated in the preceding section, given that both data are subject to stochastic errors and unobservable attributes, it becomes imperative to ascertain that the simulation sequence {x * (t)}, generated by function F, conforms to the same distribution as the real-world opinion sequence {x (t)}, in order to further substantiate the validity of our theory. Initially, utilizing the acquired parameters, we conduct 20 simulations, wherein the following analysis is performed between these 20 “simulation samples” and the real samples. Consequently, two types of visually descriptive representations are constructed: (1) a scatter/density chart in Figure 12 depicting the relationship between real opinions and simulation opinions; (2) density plots and histograms in Figure 12 portraying the disparity between “real opinions” and “simulation opinions”. In relation to (1), the greater their proximity to the y = x line, the more akin their empirical distributions become; in regard to (2), deductions can be directly inferred through a comparative assessment of the deviations in density curves. The aforementioned figures unequivocally demonstrate that both (1) and (2) exhibit concurrence between the simulation and real samples, thereby offering preliminary validation of our model/theory and parameter estimation.

Concurrently, in accordance with the test method outlined in Appendix C, we generate a sampling frame for F and T, employing random sampling techniques to extract 50 samples. This process yielded two high-dimensional samples, respectively, derived from the population of stochastic operators: F(w) and T(w) [49], which were applied for the test to determine whether F and T are equally distributed. The results of our test are meticulously documented in Table 3 (T_1_ and T_2_ are “the rule from x(0) to x(1) “and “the rule from x(1) to x(2)”, respectively). It is discernible from the results that both F and T emanate from the same distribution because of the large *p* value (which cannot refute the null hypothesis of the same distribution between F and T), thereby attesting to the veracity of the model rules expounded in our paper. Additionally, leveraging Proposition 4 in Appendix C, we can further deduce the congruity between the simulation model and the real rule: the test results in Table 3 can also ensure that our model can obtain data identically distributed with real data because the simulation rule F* has the same distribution as the real rule.

## 6. Conclusions and Prospects

### 6.1. Conclusions

In this paper, we present some theorems and simulation results related to global consensus/local consensus/chaos for answering questions 1/2/3, which are integrated into the “ideal synchronization theory”. It includes the following key conclusions: (1) The opinion formation consists of several fundamental mechanisms, namely assimilation/exclusion, social/confidence reachability, and the “cocoon room” effect, representing “a mutual approach of opinions after an interaction” (social learning rates and exclusion rates), “possibility of contact/interaction” (confidence thresholds and average network degree), and “effect of shielding group pressure” (inversely correlated with the pressure coefficient k); (2) Assimilation and high reachability lead to global consensus, assimilation and low reachability result in local consensus, and exclusion and high reachability lead to chaos; (3) A higher “cocoon room” effect can maintain the local consensus. Among them, the laws related to local consensus/chaos in (2) and (3) are the core conclusions discovered in a novel way in our paper, and more detailed contents are presented in the section of Results and Discussions, such as the impact of social reachability/the network average degree on consensus. Additionally, we have made some new discoveries that require further exploration, such as the positive correlation between social learning rate u and convergence rate, the bifurcation caused by confidence thresholds d and network average degree d*, the polarization of opinions caused by strong social exclusion (e.g., u = −1.2), and the disruptive effect of a small number of repulsors on consensus. These conclusions above are the main sociological and physical contributions of our paper.

Moreover, to address questions 1/2/3 and solve problems A/B/C, we have developed new formal methods, including the “General Model of Opinion Evolution” (a stochastic dynamical system), the proof methods of “energy decrease” and “cross-d search” (applicable to a large number of averaging dynamics), and high-dimensional statistical tests on the evolution rules of the models (to verify the consistency between the ABM models and the real world). The three theorems in this paper also serve as proofs of synchronization and chaos in models like D-W and H-K. These contents further develop and confirm the aforementioned ideal synchronization theory but belong to the mathematical and statistical contributions of our paper, mainly presented in Appendix A, Appendix B and Appendix C.

### 6.2. Prospect

Based on the aforementioned conclusions, a comprehensive framework can be devised to elucidate the evolution of large-scale, ordered opinions. Nevertheless, within the framework, there remain several internal concerns (which do not require us to modify our model) and external problems (which require us to modify the model), which necessitate future consideration. We will briefly illustrate them below.

For internal concerns, three distinct avenues merit exploration: (1) establishing theorems about synchronization (global/local consensus) under weaker conditions like connected graphs and asymmetric interactions, thereby constituting the refinement and generalization of theorems 1/2/3 in our paper; (2) conducting a reliable analysis of the bifurcation in our model, as evidenced by numerous simulation results, such as researching bifurcation points of the threshold ‘d’ when given values of ‘u’ and ‘d*’, thereby further enriching the ideal synchronization theory; (3) studying the opinion convergence rate within the model, serving to elucidating the speed of real-world opinion formation.

For external problems, while our model possesses considerable validity, there is room for enhancing its capacity for explanation. For this issue, engaging in a dialogue with pertinent social theories becomes imperative. Firstly, at the individual level, it is worthwhile to emphasize the differentiation between implicit cognition and explicit behavior. To address this, future investigations may incorporate variables that represent “private opinions/expressed opinions” discretely within the model, thereby elucidating the disparity between “attitude learning” (ideas change when influenced by others) and the “spiral of silence” (submission to external pressures). Secondly, at the system level, the framework fails to encompass more intricate external variables such as socioeconomic status, culture, and semantic networks. Although the effects of such variables can be amalgamated through the internal evolutionary attributes of opinions, bolstering a more imaginative explanation of opinion evolution, it remains important to explicate them effectively. Therefore, establishing the relationship between the opinion system and other systems emerges as a pivotal endeavor to be explored in subsequent research. Overall, addressing the external problems of our framework necessitates a comprehensive integration of existing knowledge and tools from cognitive science, social theory, and nonlinear mathematics, thereby facilitating the promotion of rigorous theoretical construction.

## Figures and Tables

**Table 1:** Ideal Synchronization Theory.

Ideal Synchronization Theory	High Reachability (H)	Cocoon House Effect (C)	Low Reachability (L)
**Assimilation (A)**	Global consensus	High: <—/—	Local consensus
		Low: <—/—	
**Exclusion (E)**	Chaos	-------------------------	Other phenomena

**Table 2:** Estimation Results.

u/Bayesian Factor (0.1–0.5)	d/Bayesian Factor (0.1–0.5)	k/Bayesian Factor (0–0.1)
0.2114; 6.72	0.178; 8.31	0.102; 5.44
**u_i_ mean**	**d_i_ mean**	**k_i_ mean**
0.094	0.102	0.037

**Table 3:** Test Results.

Values of Statistics for the Same Distribution Test	F(w) Distribution
T_1_(w) distribution	*p* value: 0.46
T_2_(w) distribution	*p* value: 0.37

**Figure A1:**
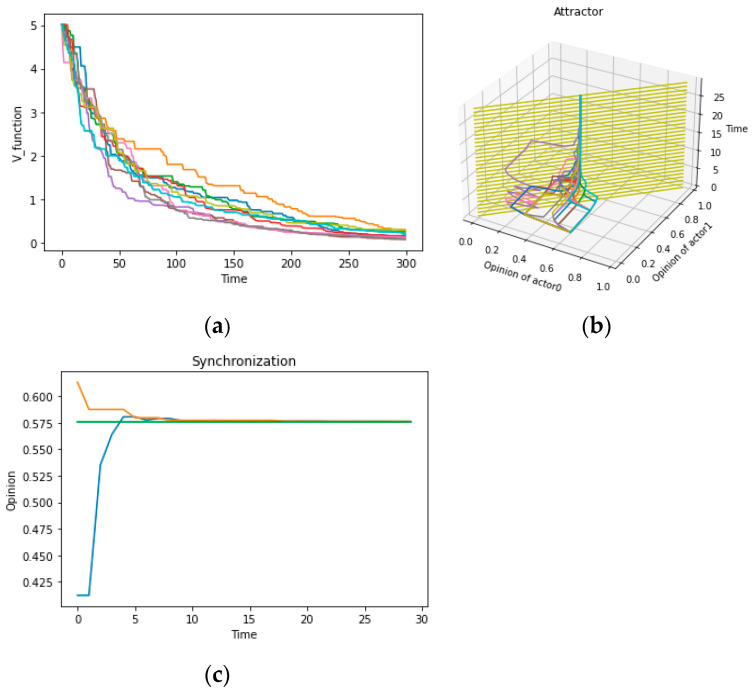
(**a**) Shows that the value of V_function is decreasing over time. (**b**) Reflects that the opinion2 of the actor0 and actor1 is gradually attracted to the plane of x1 = x2, i.e., entering a synchronous state. (**c**) The three colored lines, respectively, represent the evolution of the opinions of three individuals, and this type of chart is referred to as the O-T chart (Opinion-Time) in the following text.

**Figure A2:**
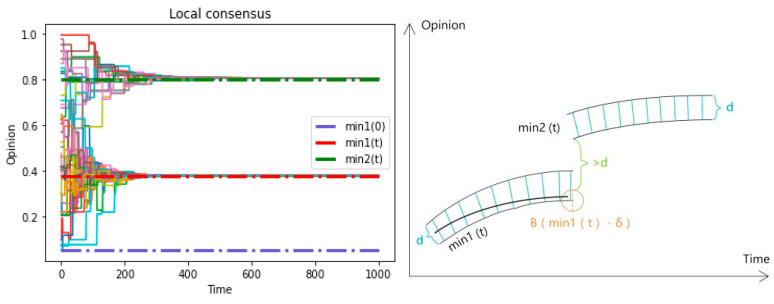
Both figures reflect the proof ideas stated in the remarks above.

**Figure A3:**
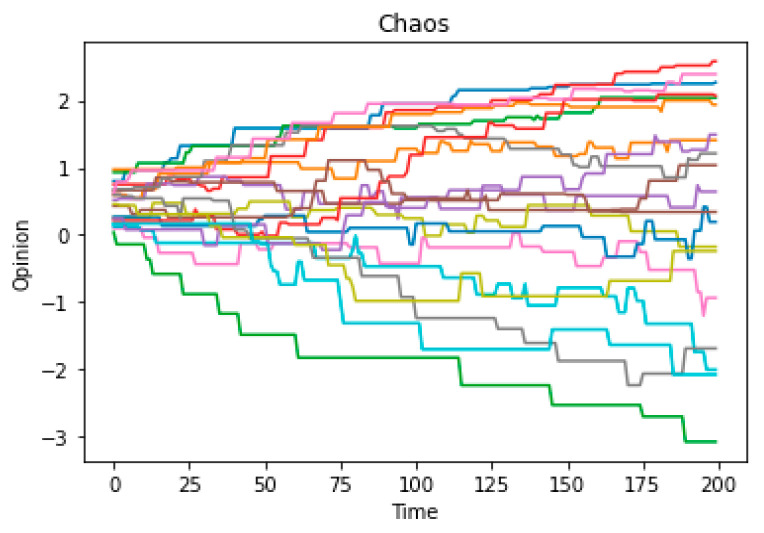
The following is an Opinion-Time chart reflecting chaos.

## Data Availability

Our data can be obtained from the link https://dataverse.harvard.edu/dataset.xhtml?persistentId=doi:10.7910/DVN/H3ZBHR, (accessed on 15 June 2023).

## Appendix A. Strict Definitions and Theorems

### Appendix A.1. Axiomatic Framework

The axiomatic framework of our model encompasses four fundamental constituents: opinion space X, social network G, an interaction event is set Ω, and the evolution rule F. Among these, X denotes the domain of admissible values for an agent’s opinion, xi. G quantifies the “influence sphere of agent i”, encompassing all agents j with whom i has the capacity to contact. Ω serves as an indicator of whether an interaction between i and j will occur subsequent to contact. F delineates the rules governing the evolution of group opinions following an interaction event w. This framework can be applied to stochastic dynamics on various social networks, providing a comprehensive methodology for modeling opinion evolution. Based on this framework, we can establish our opinion dynamics model as a quadruple (X, G, Ω, F).

### Appendix A.2. Notations

This article employs the following symbols, each serving a distinct purpose: capital letters V, E, Ω, and X, among others, are employed to denote sets; lowercase letters vi, ei, w, and xi are utilized to represent elements within a set; distinguished characters like F, G, f, and g are used to signify mappings, encompassing functions and stochastic operators. The latter notion is elaborated upon in reference [49]. Furthermore, P/m/u are employed as notational symbols denoting set functions, specifically probability measure/Lebesgue measure/abstract measure. Meanwhile, we adhere to the customary conventions, including: (1) The employment of the conjunction: “≜” or the symbol “≜” itself to denote definitions. (2) Standard operations, such as “~denotes the relation on a set, × signifies the product entity of a set/topological space/probability space within the realm of category theory, ∑ represents the summation operation, / signifies the division of real numbers or the quotient operation of a set, | • | denotes the absolute value or the cardinality of a set with respect to the given context, || • || signifies the L^2^ norm of a space”. (3) Exceptional operations: for instance, limsup/liminf denote the supremum and infimum limits of a set”, II_A_(x) is the characteristic of the set A, and h(f) represents the topological entropy of the mapping f. (4) The domain concepts can be defined as follows: Kn signifies a complete graph comprising n nodes, while Ω (x0) signifies the positive limit set of x0. In this context, we solely present the symbols and their corresponding meanings that may entail ambiguity (symbols with self-evident meanings, such as multiplication • or composite °, are omitted). These symbols adhere to the established conventions in mathematical literature. The novel symbols introduced in this paper will be elucidated and expounded upon in subsequent definitions.

### Appendix A.3. Definitions

**Definition** **A1.** *Let the actor set be* $V=\left\{v_{1},\ldots .,v_{n}\right\}$*, the binary relationship set be * E⊆(V×V)/~, and the actor-network be G=(V,E)*. (Inside, ~ is defined as* (x,y)~(y,x)*; let the element of E be written as e_ij_,* $e_{ij}=[v_{i},v_{j}]=\left[\left(v_{i},v_{j}\right)\right]$*, which is the equivalence class under the relationship ~, briefly written as {i, j}/[i, j]).*

**Remark** **A1.**
*G denotes the “social network” among individuals, such as a “friendship network” or a “mutual following network”; V represents all individuals, where “vi represents individual i”; E represents a certain form of mutual connection or friendship between individuals, such as “eij represents the mutual attention between i and j”.*

**Definition** **A2.** *Let the interaction event set be* $\Omega_{t}=\prod_{[i,j]\in V\times V/\sim }(R\backslash \{0\})$*, thus* $\left(\Omega_{t},B\left(\Omega_{t}\right),P\right)$ *constitutes a probability space. Inside,* $B\left(\Omega_{t}\right)$ *represents the Borel algebra of* $\Omega_{t}$*. The topology of* $\Omega_{t}$ *is the sub-topology of* $R^{\frac{n(n+1)}{2}}$*, and* P *is a probability measure on* $\Omega_{t}$*. Let* $\Omega =\prod_{t=0}^{\infty }\Omega_{t},(\Omega ,B(\Omega ),P)$ *be the product probability space induced by* ${\left\{\Omega_{t}\right\}}_{t\in I}$ *(w* ∈ Ω*), w_t_* ∈ $\Omega_{t}$*, and the component of w_t_ is abbreviated as* $w_{t}{}^{\left(i,j\right)}$.

**Remark** **A2.**
*In Ω_t_, w_t_ signifies the “interactions that occur at time t”, specifically w_t_ = {w_ij_: i and j are any two individuals in the social network}—where w_ij_ represents the interaction strength between individual i and individual j at time t. If w_ij_ is greater than 0, it indicates an interaction between i and j at time t, while if w_ij_ is less than 0, it implies no interaction between them at time t. Additionally, wt is a random event, meaning that “whether i/j interact or not” at time t is subject to randomness.*

**Definition** **A3.** *Let* ${\;\mathrm{Inter}}_{\mathrm{ij}}\;\left(w_{t}\right)=\left\{\begin{array}{ll} 1, & w_{t}{}^{\left(i,j\right)}>0\\ 0, & w_{t}{}^{\left(i,j\right)}\leq 0 \end{array}\right.$*, which is a random variable (obviously a measurable mapping).*

**Remark** **A3.**
*Inter_ij_(wt) denotes “the interaction between individuals i and j under the random event w_t_”. It takes a value of 1 if i and j interact, and 0 otherwise.*

**Definition A4** **(Framework and Model).** 
***Part A: General Framework***

***I. Stochastic Dynamics***
*(X, G, H, T) is a stochastic dynamic, if ①* X *is a topological space, ② G is a graph, ③* H *is a probability space, ④* T *is a* H− *measurable (or* H×X−measurable*) stochastic operator, and ⑤* ∀ω∈H *(or * ω∈H×X*) and* T(ω) *is continuous on* X.

**Remark** **A4.**
*This definition extends the definition of topological dynamical systems. According to the definition in this paper, X represents the range of opinions, and a state (x1…xn) is taken from X, representing the initial opinions of all individuals. This state continually evolves under the influence of the rule T(w), where w denotes randomly occurring interaction events, thereby capturing the evolution process of the collective opinions.*

**II. Evolution Model**

**(1) Rules**

Let $F:\Omega \to B\left({\left[0,1\right]}^{n},{\left[0,1\right]}^{n}\right)$ is a stochastic operator [49], $x\in {\left[0,1\right]}^{n},$ $u_{\mathrm{ij}}\in [-1,1]$, and ${\left[F(\omega )(x)\right]}_{\left(i\right)}:≜\left\{\begin{array}{l} 0,\tilde{F}(\omega )x<0\\ \tilde{F}(\omega )x,0⩽\tilde{F}(\omega )x⩽1\\ 1,\;\tilde{F}(\omega )x⩾1 \end{array}\right.$

① The original rule
  $$\begin{array}{l} \tilde{F}\left(\omega_{k}\right)x(i):≜{\mathrm{II}}_{\left[1,+\infty \right]}\left[\left(\sum_{j\in N(i,t,\omega )}{\mathrm{Inter}}_{\mathrm{ij}}\left(\omega_{k}\right)\right]{\left[f(\omega )x\right]}_{i}+\mathrm{II}(-\infty ,1)\left[\sum_{j}{\mathrm{Inter}}_{\mathrm{ij}}\left(\omega_{k}\right)\right]\right.\cdot x\left(j\right)\\ f(\omega ){\left(x\right)}_{i}:≜\left(1-\sum_{j}u_{ij}/N(i,t,\omega )\right)x_{i}+\frac{1}{N(i,t,\omega )}\sum_{\left(i,j)\in E(t\right)}u_{ij}x_{j}\cdot {\mathrm{Inter}}_{\mathrm{ij}}(\omega )\cdot {\mathrm{II}}_{E}\left(\left\{v_{i},v_{j}\right\}\right)\\ +\mathrm{noi}{\left(i,j,t+1\right)}_{j} \end{array}$$
② The alternative rule
  $$\begin{array}{l} \tilde{F}\left(\omega_{t}\right)x(i):≜\mathrm{II}[1,+\infty ]\left[\left(\sum_{j\in N(i,t,\omega )}{\mathrm{Inter}}_{\mathrm{ij}}\left(\omega_{t}\right)\right)\cdot \left|N\left(i,t,\omega ,x_{0}\right)\right|\right]{\left[f\left(\omega_{t}\right)x\right]}_{i}+\\ {\mathrm{II}}_{\left(-\infty ,1\right)}\left[\left(\sum_{j}{\mathrm{Inter}}_{\mathrm{ij}}(\omega_{t})\right)\cdot \left|N\left(i,t,\omega ,x_{0}\right)\right|\right]\cdot x(i) \end{array}$$
  $$\begin{array}{l} f(\omega_{t}){\left(x\right)}_{i}:≜\left(1-\sum_{j}u_{ij}/N(i,t,\omega )\right)x_{i}+\frac{1}{N(i,t,\omega )}\sum_{\left(i,j)\in E(t\right)}u_{ij}x_{j}\cdot {\mathrm{Inter}}_{\mathrm{ij}}(\omega_{t})\\ \cdot {\mathrm{II}}_{\left(0,di\right)}\left(\left|x_{i}\cdot x_{j}\right|\right)\cdot {\mathrm{II}}_{E}\left(\left\{v_{i},v_{j}\right\}\right)+noi(i,j,t+1) \end{array}$$
  Given graph G, let $S≜\left({\left[0,1\right]}^{n},\Omega ,F\right)$. It is easy to verify that S is a stochastic dynamic, namely the ‘opinion dynamic’.

**Remark** **A5.**

(1) *Let the opinion space X be a bounded set [0, 1]^n^. The image set of the rule F is also [0, 1]^n^, in order to meet the measurement requirements of ordered perspectives (most datasets map them to real values within 0–1). The mathematical definition of noi(i, j, t + 1) can be found in the following text.*
(2) *The framework includes three important parameters: d represents the confidence threshold, and i and j can interact only if the difference in their opinions is below the threshold; u_ij_ represents the social learning rate of i from j, that is, the influence of j’s opinion on i’s opinion; and k_i_ represents the compression coefficient of i, indicating the degree to which individual i perceives group pressure. There is also an important variable: N(i, t, x0, w) represents “all individuals j interacting with individual i at time t given the initial opinions x0 and the sequence of interaction events w”.*
(3) *The fundamental meaning of the above framework is that at any given time t, there exist two categories of individuals: non-participants (referred to as NP) and participants (referred to as P). The criterion for determining whether an individual j belongs to NP or P is based on whether there exists an i in the random event wk who interacts with j, that is, whether the value of Inter_ij_(wk) is zero. Based on this criterion, the opinions of individuals in NP remain unchanged, while the opinions of individuals in P are influenced by three levels: (1) their existing perspective xi, (2) the weighted average of the opinions xj of all individuals interacting with them, and (3) the average opinion of the system (noise/group pressure). Among them, the parameter u_ij_ reflects the social influence effect, and the parameter d_i_ represents the influence of bounded confidence.*
(4) *The difference between the alternative rule and the original rule lies in the fact that when the difference in opinions between individuals i and j is greater than d, under the original rule, the probability of an interaction between i and j is 0, while under the alternative rule, i and j will not interact.*

**(2) Symbols**

Given S, $x_{0}\in {\left[0,1\right]}^{n},\omega \in {\prod_{i=1}^{\infty }\Omega }_{t}$, let

$$x\left(t+1,x_{0},\omega \right):≜F\left(\omega_{t}\right)\left(x\left(t,x_{0},\omega \right)\right)$$

$$x_{i}\left(t,x_{0},\omega \right):≜x\left(t,x_{0},\omega \right)[i]$$

$\mathrm{noi}:\;{\left[0,1\right]}^{n}\times {\left[0,1\right]}^{n}\times N\to [0,1]$ is measurable

$\left(\mathrm{Given}\;x_{0}\in X,w\in H,\mathrm{stochastic}\;\mathrm{operator}\;T\right)$,

Inside, t∈N and given x(0) and ω, $x\left(t,x_{0},\omega \right),x_{i}\left(t,X_{0},\omega \right)$ can be written as x(t), $x_{i}(t)$.

**III. Probability measure**

Given $x_{0}\in {\left[0,1\right]}^{n}$, for (Ω,B(Ω),P), $P≜P_{x_{0}}$ should satisfy

① The original rule
  $$\begin{array}{l} P_{x_{0}}\left\{w:w_{t}^{ij}>0\right.\mathrm{and}\left.\left|x_{i}(t)-x_{j}(t)\right|⩾\max(d_{i}(t),d_{j}(t))\right\}=0\\ P_{x_{0}}\left\{w:\mathrm{if}\;\left|x_{i}(t)-x_{j}(t)\right|<\max(d_{i}(t),d_{j}(t)),\mathrm{then}\;w_{t}^{ij}>0\right\}>0 \end{array}$$
  $d_{i}(t):N\to [0,1]\in l^{2}$, it can be represented as $\left(d_{i}(0)\cdots d_{i}(n)\cdots \right)$
② The alternative rule
  $$\begin{array}{l} P_{x_{0}}\left\{w:w_{t}^{ij}>0\right.\mathrm{and}\left.\left|x_{i}(t)-x_{j}(t)\right|⩾\max(d_{i}(t),d_{j}(t))\right\}=0\\ P_{x_{0}}\left\{w:\mathrm{if}\;\left|x_{i}(t)-x_{j}(t)\right|<\max(d_{i}(t),d_{j}(t)),\mathrm{then}\;w_{t}^{ij}>0\right\}>0 \end{array}$$
  which is the same as the original rule.

**Part B: Usual Model**

$M_{1}$ (The symmetric/multiple-interaction/heterogeneous model)

① $P\left(\sum_{j}{\mathrm{Inter}}_{\mathrm{ij}}(w_{t})\leq 1\right)=1$ and ${\mathrm{Inter}}_{\mathrm{ij}}(w_{t})={\mathrm{Inter}}_{\mathrm{ji}}(w_{t})$;
② $\exists i\;,\;\mathrm{degree}\;\left(v_{i}\right)=n-1,d_{i}⩾\max x_{i}(0)-\min x_{i}(0)$;
③ $d_{i}(t)=d_{i}\in [0,1]$;
④ ∀i, $[0,1]∋u_{ij}=u_{ji}>0$;
⑤ noi(i,j,t)=Press(i,j,t)

$Let\;\mathrm{Pr}ess(i,j,0)=0,\mathrm{Pr}ess(i,j,t+1)=k|x_{i}(t)-x_{j}(t)|\left[\frac{1}{n}\sum x_{i}(t)-\frac{x_{i}(t)+x_{j}(t)}{2}\right]$.

$M_{2}$ (The symmetric/multiple-interaction/homogeneous model)

①’ same with $M_{1}$;
②’ $G=K_{n}$;
③’ $d_{i}(t)=d\in [0,1]$;
④’ ∀i, $u_{i}=u\in [0,1]$;
⑤’ $\forall i,k_{i}=0$.

$M_{3}$ (asymmetric/multiple-interaction)

①” G is a connected graph;
②” ∀ i, d_i_↑ d = 1;
③” $\forall i,k_{i}=0$

**Remark** **A6.**

(1) *M1 implies that there exists an agent v_i_ in the system who frequently interacts with any other agent, and these individuals are influenced by external group pressure and internal cohesion during interactions.*
(2) *M2 implies that the system is entirely determined by the “bounded confidence mechanism”, and agents are not influenced by the external group during interactions. This is a generalization of many existing bounded confidence models (including the D-W model).*
(3) *M3 implies that the system’s rules, once fixed, can combine social influence with the French-Degroot model.*

**Definition** **A5.**

$$\begin{array}{l} E(t)=E\left(t,w,x_{0}\right)=\left\{(v_{i},v_{j}):|x_{i}(t)-x_{j}(t)|<d\right\},\\ V(t)=V\left(t,w,x_{0}\right)=\left\{v_{i}\in V:\exists v_{j},\left(v_{i},v_{j}\right)\in E(t)\right\},\\ G(t)=(V(t),E(t)). \end{array}$$

**Remark** **A7.**
*V(t) represents the individuals at time t who are capable of potential interactions; E(t) represents the relationships formed by pairs (i, j) of individuals who are capable of potential interactions at time t; and G(t) represents the network composed of V(t) and E(t), which is a subnetwork of G (since the “set of individuals/relationships capable of an interaction at time t” is necessarily a subset of the “set of individuals V/relationship set E” in the original social network).*

**Definition** **A6.** *Given measure space* $\left(V,2^{V},u\right)$, *u is the count measure on V,* $x_{i}\left(t\right)\;\mathrm{is}\;\mathrm{measurable}\;\mathrm{on}\;V$*, the average opinion of the time t is written as* $\bar{x\left(t\right)}$ *or* $E_{i}x_{i}(t)≜\frac{\int_{V}x_{i}(t)du}{\left|V\right|}=\frac{\sum_{i}x_{i}(t)}{\left|V\right|}$.

**Remark** **A8.**
*E_i_x_i_(t) denotes the arithmetic mean of the individual opinions at time t, representing the average state of the opinion evolution system at time t.*

**Definition** **A7.** *Let* ε: $\Omega \to {\left[0,1\right]}^{n}$ *is a mapping:*

ε *is a generalized fixed point of F* $\Leftrightarrow F(\omega_{t})\varepsilon (\omega_{t})=\varepsilon (\omega_{t})$ *a.s.*
ε *is a stochastic fixed point of F* ⇔ε *is a generalized fixed point of F, and* ε *is* Ω- *measurable.*
ε *is a stochastic global attractor of* F ⇔*,* ε  is a stochastic fixed point of F*, and given* $x_{0}\in {\left[0,1\right]}^{n}$*,* $P\{w:t\to \infty ,F(w_{t})\circ \cdots \circ F(w_{1})(x_{0})\to \varepsilon \}=1$.
ε *is a stable stochastic fixed point of* F ⇔ε *is a stochastic fixed point of* F*, and* P *{* ω:∀ε>0*,* ∃δ(ε)>0*, if* $\left\|\varepsilon_{0}-\varepsilon \right\|$ <δ(ε)*,* $\forall w={\left\{\omega_{i}\right\}}_{i=1}^{\infty }$*,* $||F\left(\omega_{t}\right)\cdots F\left(\omega_{1}\right)\varepsilon -F\left(\omega_{t}\right)\cdots F\left(\omega_{1}\right)\varepsilon_{0}||<\varepsilon$*} = 1*.

**Remark** **A9.**

(1) *“The stochastic fixed point x0” implies that “after random occurrences of any opinion interaction w_t_, the viewpoint state x0 remains unchanged, i.e., F(w_t_)x0 = x0”.*
(2) *“The stochastic global attractor x0” implies that “x0 is a stochastic fixed point, and for any opinion state x*, after undergoing numerous evolutions according to almost any opinion rule F(w_t_), it will approach/be attracted to the vicinity of state x0”.*
(3) *“The stochastic stable fixed point x0” implies that “x0 is a stochastic fixed point, and for opinion states x* around x0 at time t, even after infinite evolutions according to the opinion rule F(w_t_), they will still be in the vicinity of x0”.*

**Definition** **A8.** *Let* $d_{v}(t)≜d_{v}\left(t,x_{0},\omega \right)={max}_{vi,vj\in V}\left|x_{i}(t)-x_{j}(t)\right|,$ $d_{v}=\underset{t}{\lim}\sup d_{v}(t)$

① $P\{W:|dv|<\varepsilon \}=1\Leftrightarrow \mathrm{Given}w\mathrm{and}x_{0},\;x(t)\mathrm{has}\varepsilon -consensus.$
② $P\left\{W:\forall \varepsilon >0,\left|d_{v}\right|<\varepsilon \right\}=1\Leftrightarrow {\mathrm{Given}\;w\;\mathrm{and}\;x}_{0},\;x(t)\;\mathrm{has}\;\mathrm{quasi}\text{-}\mathrm{consensus}.$
③ $\begin{array}{l} \exists K\subseteq {\left[0,1\right]}^{n},A\subseteq \Omega ,m(K)=1\mathrm{and}P(A)=1,\forall x_{0}\in K,w\in \Omega ,P\left\{W:\forall \varepsilon >0,\left|d_{v}\right|<\varepsilon \right\}=1\\ \Leftrightarrow ({\left[0,1\right]}^{n},\Omega ,F)\mathrm{has}\;\mathrm{quasi}\text{-}\mathrm{consensus}.\\ \exists K\subseteq {\left[0,1\right]}^{n},A\subseteq \Omega ,m(K)=1\mathrm{and}P(A)=1,\forall x_{0}\in K,w\in \Omega , \end{array}$
④ $\begin{array}{l} x(t)\;\mathrm{has}\;\mathrm{quasi}\text{-}\mathrm{consensus}\;\mathrm{and}\;F\;\mathrm{has}\;\mathrm{stable}\;\mathrm{stochastic}\;\mathrm{fixed}\;\mathrm{points}\\ \Leftrightarrow ({\left[0,1\right]}^{n},\Omega ,F)\mathrm{has}\;\mathrm{stable}\;\mathrm{quasi}\text{-}\mathrm{consensus}. \end{array}$

**Remark** **A10.**
*Quasi-consensus represents the probability of the system achieving global consensus as 1, and stable quasi-consensus indicates that it is less affected by initial disturbances.*

**Definition** **A9.** *Given* $\left({\left[0,1\right]}^{n},\Omega ,\;F\right)$*, define the following concepts:*

① $R\left(\omega ,x_{0}\right):≜\left\{(i,j)\in V\times V:\right.$ $\left|x_{i}(t)-x_{j}(t)\right|$ *is convergent and* $\left.\underset{t}{\lim}\left|x_{i}(t)-x_{j}(t)\right|=0\right\}$,* it can be verified as an equivalent relationship;*
② $\mathrm{Cluster}\left(\omega ,x_{0}\right):≜(V\times V)/R\left(\omega ,x_{0}\right)$;
③ $\mathrm{Nconsensus}\left(w,x_{0}\right):≜\left|\mathrm{Cluster}\left(w,x_{0}\right)\right|$;
④ $\exists K\subseteq {\left[0,1\right]}^{n},A\subseteq \Omega$ *and* P(A)=1*,* ∀ω∈A*,* ∀x∈K*,* Nconsensus (ω,x)= Const ∈N*,*
  $$\mathrm{Nconsensus}(A,K):≜\;\mathrm{consensus}(w,x),N(\mathrm{con}\mathrm{sensus}):≜N\left(A,\;{\left[0,1\right]}^{n}\right)$$

**Remark** **A11.**
*The cluster (w, x0) represents the consensus clustering of the system’s opinions that eventually form, given all the interactive events w that will occur and the initial opinion state x0. Nconsensus represents the number of consensuses reached after infinite evolutions of the system.*

**Definition** **A10.** ∃ *uncountable set* $K\subseteq {\left[0,1\right]}^{n}$**,** 

$$\begin{array}{l} \forall x,y\in K,\underset{n}{\lim}\sup d(F^{n}x,F^{n}y)>0,\underset{n}{\lim}\inf d(F^{n}x,F^{n}y)=0\\ \Leftrightarrow ({\left[0,1\right]}^{n},\Omega ,F)\mathrm{is}L-Y-\mathrm{chaos} \end{array}$$

**Remark** **A12.**
*The term “LY chaos” in the above definitions refers to “the state of disorderly fluctuations in opinions where a significant number of individuals are trapped in them”.*

### Appendix A.4. Main Theorems

Drawing upon the established model and axiomatic definition expounded earlier, we hereby posit the subsequent tripartite theorems to address questions 1/2/3, correspondingly. The remarks illustrate the methods and strategies of our proof for these three theorems.

**Theorem** **A1.**
*Under M1, ([0, 1]^n^, Ω, F) has stable quasi-consensus.*

**Remark** **A13.**

(1) *The theorem provides a significant conclusion to address Question 1. Referring to the definition of M1, it can be inferred that the magnitudes of the social learning rate (u) and group pressure (k) are almost unrelated to global synchronization. However, the sequence of network degrees and the threshold of confidence have a considerable impact on it. Furthermore, Theorem 1 explains a phenomenon known as the “active agent effect”, wherein “in a system of opinions, if there exist active agents capable of interacting with any other individual, and there is an assimilation effect among individuals, then the system can evolve towards global consensus”. This inference from the theorem can also serve as a synchronicity conclusion for models such as Deffuant et al.*
(2) *The proof strategy for the theorem can be illustrated as shown in the following figure, known as the “energy decrease method” (by stochastic functional analysis and differential dynamical systems theory). It implies that the synchronicity of the model (as seen in the upper-left diagram) can be understood as a specific type of dynamical system attractor (as seen in the upper-right diagram). In other words, asynchronous system states will evolve towards synchronous states under rule F. As depicted in the upper-right diagram, the opinion values of x1 and x2 gradually converge around x1 = x2. Based on this, the specific approach involves constructing an energy function V that represents the degree of deviation of the system state relative to the synchronization point. It is then demonstrated that the infimum of V remains nearly constant and is unaffected by w (as seen in the lower-left diagram), leading to the inclusion of the system’s positive limit set within the points x0 that keep V invariant (the points where V equals 0). It is further explained that x0 possesses global attractiveness and stability, thus resulting in synchronicity.*

**Theorem** **A2.** $\mathrm{Under}\;M2,\;\mathrm{if}\;u⩽1/2,n=[1/d]+1,\forall x_{0}\in {\left[0,1\right]}^{n},P\left(w:N\mathrm{consensus}\;\left(w,x_{0}\right)⩽n\right)=1$.

**Remark** **A15.**

(1) *The theorem provides a direction to address Question 2. It provides an upper bound on the number of consensuses when a confidence threshold is determined and illustrates that the threshold’s magnitude is inversely related to the quantity of consensus. The sociological interpretation states that “when the individual tolerance level is high, the system exhibits fewer types of consensus” (i.e., “local consistency in opinion formation”).*
(2) *The proof strategy for the theorem also needs to be referred to in the following figure, named the “cross-d search” method. The approach can be explained by considering the following figure: A. Proving the convergence of the opinion state x(t) requires first determining the minimum value min1(t) and then proving that “for all xj(t) that differ from min1(t) by less than d, they can converge to a small neighborhood B = B(min1(t), δ) of min1(t)”. It is further demonstrated that “there exists a moment T at which a minimum value min2(t) outside of B, with a distance greater than d from B, exists”. Finally, it is shown that min2(t) satisfies the same relation with min3(t)…mini(t) satisfies with mini + 1(t), thus deducing that the opinions x(t) can converge to (x1…xn) for any w; B. The upper bound on the number of consensuses is derived by utilizing the convergence of the opinion states and the probability conditions of interactions among individuals.*

**Theorem** **A3.** *Under M3, if* $\tilde{F}$ *is an expanding map, thus* P{w:x(t) has L−Y chaos}=1*, and it will not reach quasi-consensus.*

**Remark** **A16.**

(1) *Theorem 3 addresses Question 3 under the condition of an autonomous system. Its sociological interpretation states that “when there is a certain level of social exclusion among individuals and interactions between actors continue, the opinion system will exhibit chaotic phenomena”.*
(2) *The intuitive representation of chaotic phenomena can be observed in the below diagram.*

## Appendix B. The Proof of Theorems

### Appendix B.1. The Proof of Theorem 1

**Theorem A4** **(stochastic Lyapunov’s theorem).** *Given a stochastic dynamics* $\left(X,\Omega ,F\right)$, Ω *is a neighborhood of zero point O; if for all* x∈Ω*, we have V-functions that satisfy the following requirements of (1) and (2) in Theorem 0-2, and* ε=0 *is a stochastic fixed point of F, then* O *is a stable stochastic fixed point of F*.

**Proof.**  From (1), $\forall w={\left\{w_{t}\right\}}_{t=0}^{\infty }\in A$ and P(A)=1

I. For every ε>0 and $\Omega_{\varepsilon }=\{x:||x||<\varepsilon \}\subset \Omega$, let $c=\underset{x\in \partial \Omega_{\varepsilon }}{\min}V(x)>0$. According to the intermediate value theorem, we know that ∃δ>0, when $x\in \Omega_{\delta }=\{x:||x||<\delta \}$, 0⩽V(x)<C holds.
II. $\Delta V\left(x,w_{t}\right)\leq 0$ implies that $\forall x_{0}\in \Omega_{\delta },0⩽V\left(x\left(t,x_{0},\omega \right)\right)⩽V\left(x_{0}\right)<c=\underset{x\in \partial \Omega_{\varepsilon }}{\min}V(x)$, thus $x\left(t,x_{0},w\right)\in \Omega_{\varepsilon }$.

Namely, when $x\in \Omega_{\delta }$, ∀ω∈Ω, $\left\|x\left(t,x_{0},w\right)\right\|=\left\|F\left(\omega_{t-1}\right)\circ \cdots \circ F\left(\omega_{0}\right)x_{0}\right\|<\varepsilon$. In summary, $P\left\{w:\mathrm{zero}\;\mathrm{point}\;\mathrm{is}\;\mathrm{stable}\right\}=1$, thus the zero point O is the stable stochastic fixed point. □

**Theorem A5** **(stochastic Lasalle’s theorem).** *Given the stochastic dynamics* (X,Ω,F), $x_{0}\in X$, $\Omega \left(x_{0}\right)=\left\{y\in X:\forall w\in A,t\to \infty ,F_{\left[W_{0}\cdots W_{t-1}\right]}\left(x_{0}\right)\to y\right\}$, F *is continuous, and there is a function* V:X→R *satisfying:*

(1) ∃A⊆Ω*,*$P(A)=1,\forall w=\left(w_{1}\cdots w_{n}\cdots \right)\in A,$ $\Delta V\left(X,w_{t}\right)≜V\left(F\left(w_{t}\right)\circ x\right)-V(x)\leq 0$.
(2) V *is a positive definite function and is bounded*.
(3) $\exists c\in R,P\left(w:\underset{t}{\inf}V(x(t,x_{0},w))=c\right)=1$.

*Then,* ∃c, $P(w:\underset{t}{\lim}x\left(t,x_{0},w\right)=x^{*}$ *and* $x^{*}\in B,$ B *is a invariant subset of* $V^{-1}(c)\cap \{x:\forall w\in A,\Delta V(w,x)=0\})=1$.

**Proof.**  ∀w∈Ω, $\forall x_{0}=x(0)\in {\left[0,1\right]}^{n}$, we have the iteration sequence $x(t+1)=F\left(w_{t}\right)x(t)$,

① Since $x(t)=x\left(t,x_{0},w\right)\in {\left[0,1\right]}^{n}$ is a compact sequence, and ${\left\{x_{t}\right\}}_{t=0}^{\infty }$ is bounded, then ${\left\{x_{t}\right\}}_{t=0}^{\infty }$ must have a convergent subsequence, written as $\left\{x_{t_{k}}\right\}\to y\in \Omega \left(x_{0}\right)$, thus $\Omega (x_{0})\neq \phi$.
② $\forall y\in \Omega \left(x_{0}\right),\exists w\in A$, because $V\left(x\left(t,x_{0},w\right)\right)$ has a lower bound and it is non-increasing, then ∃c=c(w), $\underset{t\to \infty }{\lim}V\left(x\left(t,x_{0},w)\right)=c\right.$ (c is related to w). Furthermore, from the continuity of V, we know that $\exists w\in \Omega ,\underset{t_{k}\to \infty }{\lim}V\left(x\left(t_{k}\right),x_{0},w\right)=V(y)=c$ (this is because $\left\{V\left(x\left(t_{k}\right)\right)\right\}$ is a subsequence of $\left\{V\left(x\left(t\right)\right)\right\}$, and the limit of a subsequence is equal to the limit of the sequence). ∀w∈A, since c(w)=const, we obtain the conclusion that
  $$V\left(\Omega \left(x_{0}\right)\right)=\left\{V\left(y\right):y\in \Omega \left(x_{0}\right)\right\}=\left\{V\left(\lim x_{t_{k}}\right)\right\}=c,\;\mathrm{then}\;P\left(\Omega \left(x_{0}\right)\subset V^{-1}(c)\right)=1$$
③ $\forall y\in \Omega \left(x_{0}\right),w\in A,t\in N,$
  $$\Delta V(y)=\Delta V\left(w_{t},y\right)=V\left(F\left(w_{t}\right)y\right)-V(y)=V\left(F\left(w_{t}\right){}^{\circ}\underset{t_{k}}{\lim}x\left(t_{k},x_{0},w\right)\right)-V(y)$$

If $F\left(w\right)\left(x\right)$ is continuous for x, then

$$\Delta V(y)=\underset{t_{k}}{\lim}V\circ F\left(W_{t_{k}}\right)\circ x\left(t_{k}\right)-\underset{t_{k}}{\lim}V\left(x\left(t_{k}\right)\right)≜\underset{t_{k}}{\lim}V_{2}\left(t_{k}\right)-\underset{t_{k}}{\lim}V_{1}\left(t_{k}\right)=V_{2}-V_{1}$$

The former/the latter can be, respectively, denoted by $\left\{V_{t_{1}+1}\cdots V_{t_{k}+1}\cdots \right\},\left\{V_{t_{1}}\cdots V_{t_{k}}\right\}$, and let $V_{1}=\inf\left\{V(x(t_{k})):t_{k}\in N_{k}\subseteq N\right\}=\underset{t}{\mathrm{Inf}}V(x(t,x_{0},w))=c$, we know the below inequality:

$$\forall t_{k}\in N_{k},V_{2}\left(t_{k}\right)=V\left[\cdot F\left(\omega_{t_{k+1}+1}\right){}^{\circ}x\left(t_{k},x_{0},w\right)\right]⩽V\left[x\left(t_{k},x_{0},w\right)\right]⩽V_{1}\left(t_{k}\right),$$

so $\underset{t}{\inf}V(x(t))⩽V_{2}⩽V_{1}⩽\underset{t}{\inf}V(x(t)),V_{2}=V_{1}$, then ΔV(y)=0. Therefore $\Omega \left(x_{0}\right)\subseteq \{x:\forall (w)\in A,\forall w\in (\omega ),\Delta V(w,x)=0\}$. From ②③, $P\left(\Omega \left(x_{0}\right)\subseteq V^{-1}(c)\cap \{x:\Delta V(x)=0\}\right)=P(A)=1$ holds. □

**Proposition A1** **(measurability).** $\forall x\left(0\right)\in {\left[0,1\right]}^{n},$ *for* $x(t)=\tilde{F_{\mathrm{new}\;}}(w,t)x(0)$*, the mapping* ${\tilde{F_{\mathrm{new}\;}}}_{,x_{0}}\left(w,t\right):≜\tilde{F_{\mathrm{new}\;}}\left(w,t\right){}^{\circ}X\left(0\right)≜F\left(w_{t-1}\right){}^{\circ}\cdots {}^{\circ}F\left(w_{0}\right)(x(0))$ *is a measurable mapping relative to* $\Omega =\prod_{t=0}^{\infty }\Omega_{t}$*. Namely, if A is measurable, then* {w:x(t)∈A} *is measurable*.

**Proof.**  Let $P_{i}:\Omega =\prod_{t}\Omega_{t}\to \Omega_{i}$ is a projective mapping, $\forall x(0)\in {\left[0,1\right]}^{n},\forall w\in \prod_{i=1}^{\infty }\Omega ,$ we know by conditions that $x(t,w)={\tilde{F_{\mathrm{new}\;}}}_{x_{0}}(w,t)=\left(F\circ P_{t-1}\right)(w)\circ \cdots \circ \left(F\circ P_{1}\right)(w)\left(F\circ P_{0}\right)(w)\left(x_{0}\right)$. Therefore, ${\left(F\circ P_{0}\right)}_{x_{0}}:\Omega \to {\left[0,1\right]}^{n}$ is apparently a mapping, ${\left(F\circ P_{1}\right)}_{x_{1}}:\Omega \to {\left[0,1\right]}^{n}$ is the same, so we use the induction method below:

(1) When t=1 we have $\left(F\circ P_{1}\right)(w)\left(F\circ P_{0}\right)(w)\left(x_{0}\right),$ measurability clearly holds true.
(2) If the conclusion holds true when t=n−1, we know that
  ① Given $x_{0}\in {\left[0,1\right]}^{n}$, define $x^{**}(T)≜T(x)$, thus:
    When $t=n,x(t,w)={\tilde{F}}_{new_{,}x_{0}}(w,t)=F\left(P_{n-1}\circ w\right)\circ x_{n-1}(w)=F\left(P_{n-1}\circ w\right)\circ {\tilde{F}}_{\mathrm{new}\;,x_{0}}\left(w,t-1\right)$, ∘ in the formula represents function composite and matrix multiplication. To illustrate the measurability of ${\tilde{F}}_{new,x_{0}}$ when t=n, we need to verify the measurability of $P_{n-1}$, F relative to w and $w_{n-1}$. Because $P_{n-1}$ is continuous (projective mapping), it is also measurable, so we will verify the measurability of F.
  ② For F, given w, if can be represented by a matrix below:
    $$\begin{array}{l} {\tilde{F}}_{{\mathrm{new},x}_{0}\;}(\omega ,t)(i)\\ =(x_{1}\ldots x_{i}\ldots x_{n})\left[\begin{array}{l} \left(1/N(i,t)\right)\cdot u_{i1}\cdot \mathrm{int}er_{i1}(w_{t})\\ \ldots \ldots \ldots \ldots \ldots \\ \left(1-u_{ii}\right)\\ \ldots \ldots \ldots \ldots \ldots \\ \left(1/N(i,t)\right)\cdot u_{in}\cdot \mathrm{int}er_{in}(w_{t}) \end{array}\right]+\left(\sum_{j}k\cdot (x_{i}-x_{j})\cdot \left(\sum_{V_{m}\in V}x_{m}-\frac{x_{i}+x_{j}}{2}\right)\cdot Inter_{ij}\left(w_{t}\right)\right) \end{array}$$

Among this, $N_{i}\left(t\right)=\sum_{j}{\mathrm{inter}}_{ij}(w_{t})$ is measurable by the definition. Other algebraic operations are also measurable; thus, F is measurable (inside, $B\left(R^{n},R^{n}\right)$ has a strong topology induced by the operator 2-norm, and $R^{n}$ has the topology induced by a Euclidian metric). To sum up, $\forall x_{0}\in {\left[0,1\right]}^{n}\subset R^{n},\forall t,\;\tilde{F_{x_{0}}}$ is measurable, then $x\left(t,x_{0},w\right)=\tilde{F_{x_{0}}}\left(w,\cdots w_{t}\right)$ is measurable relative to $w=\left(w,\cdots w_{t}\right)$. Furthermore, we know that $\forall x_{0}\in {\left[0,1\right]}^{n}\subset R^{n}$,

$$\begin{array}{l} P\left(\left\{w\in \Omega :\left\|x\left(t,x_{2},w\right)-(a)\right\|<\varepsilon \right\}\right)=P\left\{w=\sum_{i=1}^{n}{\left(x_{i}\left(t,x_{0},w\right)-a\right)}^{2}<\varepsilon^{2}\right\}\\ \left.=P\left(w:\Sigma {\left(P_{i}\circ x\left(t,x_{0},w\right)-a\right)}^{2}<\varepsilon^{2}\right)\right) \end{array}$$

Namely, the probability of ε− consensus makes sense. □

**Lemma** **A1.** *For* $M_{1}/M_{2},\forall w\in \Omega ,\forall t\in N,E_{i}x_{i}(t)=E_{i}x_{i}(0)$.

**Proof** .

① when t=1, $E_{i}x_{i}(1)=\frac{1}{n}\sum x_{i}(1)=\frac{1}{n}\left(\sum_{k\notin V(1)}x_{k}(0)+\sum_{\left(i,j)\in E(1\right)}\left[u_{\mathrm{ij}}x_{i}(0)+(1-u_{i}{}_{j})x_{j}(0)+\right.\right.$ $\left.\left.\left(1-u_{\mathrm{ij}})x_{j}(0)+u_{\mathrm{ij}}x_{i}(0\right)\right]\right)=\frac{1}{n}\left(\sum_{k\in V(1)}x_{i}(0)\right.\left.\sum_{(i,j)\in E\left(1\right)}\left[x_{i}(0)+x_{j}(0)\right]\right)=\frac{1}{n}\sum x_{i}(0)=\sum E_{i}x_{i}(0)$
② if the conclusion holds when t=n, then when t=n+1, thus we have $E_{i}x_{i}(n+1)=\frac{1}{n}\left(\sum_{k\in V\left(1\right)}x_{k}(n)+\sum_{(i.j)\in E\left(1\right)}\left[u_{\mathrm{ij}}x_{i}(n)+\left(1-u_{\mathrm{ij}}\right)x_{j}(n)+\right.\right.\left.\left.\left(1-u_{\mathrm{ij}})x_{j}(n)+u_{ij}x_{i}(n\right)\right]\right)$ $=E_{i}x_{i}(n)=E_{i}x_{i}(0)$, so it also holds.

From ①②, $\forall t\in N,E_{i}X_{i}(t)=E_{i}X_{i}(0)$. □

**Lemma A2** **(stochastic system).** ∀w∈Ω, $P\{F\left(w_{t}\right)$ *can constitute a dynamical system on* ${\left[0,1\right]}^{n}\}≜P\left(w:F(w_{t})\left({\left[0,1\right]}^{n}\right)={\left[0,1\right]}^{n}\right)=1$.

**Proof.**  

$P(F(w_{t})\;is\;a\;surjection)=1$

$⇐F^{*}\left(w_{t}\right)$ is a surjection and $P\left(F\left(w_{t}\right)=F^{*}\left(w_{t}\right)\right)=1$ $⇐F^{*}\left(w_{t}\right)$ is nonsingular $⇐\left|F^{*}\left(wt\right)\right|\neq 0$ □

**Lemma** **A3.** *For* M1*, if* $u⩾\frac{1+\left|k\right|}{2}$*, then* ∀w,F(w) *has a generalized fixed point on* ${\left[0,1\right]}^{n}$.

**Proof.**  ∀w∈Ω, From the linearity of F, we know that $||F(w)||=\underset{x\neq y}{\sup}\frac{||F\left(w\right)x-F\left(w\right)y||}{||x-y||}=\underset{||x||=1}{\sup}||F(w)(x)||$ $\mathrm{Written}\;noi\left(i,j,t\right)\mathrm{as}\;\mathrm{noi}$, based on the definitions,

$$\begin{array}{l} {\left\|F(w)x\right\|}^{2}={\left\|{\left(x_{k}\right)}_{k\notin v(t)}\right\|}^{2}+{\left\|{\left((1-u_{ij})xi+\sum_{j}u_{ij}x_{j}\cdot \mathrm{int}er_{ij}(w_{t})+noi\right)}_{i\in V(t)}\right\|}^{2}\\ ⩽\sum_{k\notin [t)}{\left|x_{k}\right|}^{2}+\sum_{(i,j)\in E\left(t\right)}\left({\left[(1-u_{ij})x_{i}+u_{ij}x_{j}\right]}^{2}+{\left[(1-u_{ij})x_{j}+u_{i}x_{i}\right]}^{2}+2{\left|noi\right|}^{2}+\right.\\ \left.2\left(\left[(1-u_{ij})x_{i}+u_{ij}x_{j}\right]-\left[(1-u_{ij})x_{j}+u_{ij}x_{i}\right]\right)\;\mathrm{noi}\right)⩽||x{||}^{2}=\sum_{i}{\left(x_{i}\right)}^{2}, \end{array}$$

then we need

$$\begin{array}{l} \left[{\left(1-u_{ij}\right)}^{2}\left(x_{i}^{2}+x_{j}^{2}\right)+4u_{ij}(1-u_{ij})x_{i}x_{j}+u_{ij}{}^{2}\left(x_{i}^{2}+x_{j}^{2}\right)\right]+2{\left|noi\right|}^{2}+2\left[(1-2u_{ij})\left(x_{i}-x_{j}\right)\right]\;\mathrm{noi}\;\\ =\left[\left(2u_{ij}{}^{2}-2x+1\right)\left(x_{i}^{2}+x_{j}^{2}\right)+4u_{ij}(1-u_{ij})x_{i}x_{j}\right]+2{\left|noi\right|}^{2}+(2-4u_{ij})\left(x_{i}-x_{j}\right)noi\\ \leq x_{i}^{2}+x_{j}^{2}(\forall i,j\in L). \end{array}$$

Namely,

$$\begin{array}{l} u>\frac{1}{2},\;\left(2u_{ij}{}^{2}-2u_{ij}\right)\left(x_{i}^{2}+x_{j}^{2}\right)+4u_{ij}(1-u_{ij})x_{i}x_{j}+2{\left|noi\right|}^{2}+(2-4u_{ij})\left(x_{i}-x_{j}\right)noi\\ =\left(2u_{ij}{}^{2}-2u_{ij}\right)\left(x_{i}^{2}-2x_{i}x_{j}+x_{j}^{2}\right)+2{\left|noi\right|}^{2}+(2-4u_{ij})\left(x_{i}-x_{j}\right)\;\mathrm{noi}\;\\ \left.=\left(2u_{ij}{}^{2}-2u_{ij}\right){\left(x_{i}-x_{j}\right)}^{2}+(2-4u_{ij})noi\right]\left(x_{i}-x_{j}\right)+2{\left|noi\right|}^{2}\leq 0, \end{array}$$

Let $d_{ij}=x_{i}-x_{j}\in [-1,1]$, then $\left[\left(2u_{ij}{}^{2}-2u_{ij}\right)d_{ij}+(2-4u_{ij})noi\right]d_{ij}+2{\left(noi\right)}^{2}\leq 0$ holds for all $d_{ij}\in [-1,1]$, thus $g\left(d_{ij}\right)=\left(u_{ij}{}^{2}-u_{ij}\right)d_{ij}^{2}+noi(1-2u_{ij})d_{ij}+|noi{|}^{2}⩽0$ holds for all $d_{ij}\in [-1,1]$. ⇐ since $\left(u_{ij}{}^{2}-u_{ij}\right)d_{ij}^{2}⩽0$, we have $\mathrm{noi}\;(i,j)[\mathrm{noi}\;(i,j)+(1-2u_{ij})dj]⩽0$. From the same positive and negative of $d_{ij}$ and noi (i,j)

① $d_{i}{}_{j}>0$ and $noi(i,j)>0,noi(i,j)⩽(2u_{ij}-1)d_{ij}$
② $d_{ij}<0$ and $\mathrm{noi}(i,j)<0,\mathrm{noi}(i,j)⩾(2x-1)d_{i}{}_{j}$

Thus, $2u_{ij}-1⩾\frac{noi(i,j)}{d_{ij}}=|\mathrm{noi}(i,j)/x_{i}-x_{j}|$ holds, we just need to compute its upper bound:

$$\left|noi\left(i,j\right)\right|/\left|x_{i}-x_{j}\right|=|k||x_{i}-x_{j}||\mathrm{maxmin}ij|/|x_{i}-x_{j}|=|k|\cdot |\frac{x_{i}+x_{j}}{2}-\frac{1}{n}\sum_{i}x_{i}|⩽|k|$$

So when $u⩾\frac{1+|k|}{2}$,  F(w) has a fixed point $\varepsilon_{w}$. Define ε:Ω→X as $\varepsilon (w)=\varepsilon_{w}$. Therefore, F has a generalized fixed point ε. □

**Lemma** **A4.** *Under* $M_{1}/M_{2}$*, for* $({\left[0,1\right]}^{n},\Omega ,F),$ $\varepsilon \left(x_{0}\right)={\left(E_{i}x_{i}(0)\right)}_{i=1}^{n}$ *is a stochastic fixed point of F*.

**Proof** .

(1) Generalized fixed point

From Lemma A2, [0, 1]^n^ is invariant relative to F(w_t_), so the phase space of our system is invariant. Then, based on Lemma A3, $F\left(w_{t}\right)$ has a generalized fixed point, which can be obtained by using the following methods: Given $w_{t}\in \Omega_{t}$, we can find all (i,j) that make $Inter_{ij}\;(w_{t})=1$. Arbitrarily chosen a pair of (i,j), then we know

$$\begin{array}{l} \left(1-u_{ij}\right)x_{i}+u_{ij}x_{j}+noi(i,j)=x_{i};\;\mathrm{namely},\;u_{ij}\left(x_{j}-x_{i}\right)=-noi(i,j)\\ \left(1-u_{ij}\right)x_{j}+u_{ij}x_{i}-noi(i,j)=x_{j};\;\mathrm{namely},\;u_{ij}\left(x_{i}-x_{j}\right)=noi(i,j) \end{array}$$

Namely, $x_{i}-x_{j}=\frac{noi(i,j)}{u_{ij}}$. Let $\varepsilon (w)=\left(\cdots x_{i}\cdots x_{j}\cdots \right),x_{i}=x_{j}+\frac{noi\left(i,j\right)}{u_{ij}}$. Then, if $x_{i}=x_{j}$, $\frac{\;\mathrm{noi}(i,j)}{u_{ij}}=0$. Denote $\varepsilon (x(t,x0,w))=(E_{i}X_{i}(t)\cdots E_{i}X_{i}(t)\cdots E_{i}X_{i}(t))$. From Lemma A1, it can be written as $\varepsilon (x_{0})=(E_{i}X_{i}(0)\cdots E_{i}X_{i}(0)\cdots E_{i}X_{i}(0))$ and is a fixed point of F(w) apparently. Since w∈Ω is arbitrarily chosen, it is a generalized fixed point of F.

(2) Measurability

$\varepsilon \left(x_{0}\right)={\left(Ex(0)\right)}_{i-1}^{n}$ is a constant mapping, so it is continuous, and then it is measurable. From (1) (2), $\varepsilon \left(x_{0}\right)$ is a Ω—measurable generalized fixed point of F, so $\varepsilon \left(x_{0}\right)$ is a stochastic fixed point of F. □

**Lemma** **A5.** *Under M1, if* $y(t)=x(t)-\varepsilon \left(x_{0}\right)$*, then* $y(t+1)=F(w_{t})y(t)$*. Namely, the evolutionary rule of* y(t) *is same with* $x\left(t\right)$.

**Proof.**  Given $x_{0}\in {\left[0,1\right]}^{n},w\in \Omega$ arbitrarily, let $\varepsilon \left(x_{0}\right)={\left(E_{i}x_{i}(0)\right)}_{i\in I}$. From $y(t)=x(t)-\varepsilon \left(x_{0}\right)$, we have $x(t)=y(t)+\varepsilon \left(x_{0}\right)$, thus the below conclusion holds:

① if $\forall j,{\mathrm{Inter}}_{\mathrm{ij}}\left(w_{t}\right)=0$, then $y_{i}(t+1)=y_{i}(t)$
② if $\exists j,{\;\mathrm{Inter}}_{\mathrm{ij}}\;\left(w_{t}\right)=1$, then
  $$x_{i}(t+1)={\left[y(t+1)+\varepsilon \left(x_{0}\right)\right]}_{i}=(1-u_{ij})\left(y_{i}(t)+\varepsilon^{i}\left(x_{0}\right)\right)+u_{ij}(y_{j}(t)+\varepsilon^{j}\left(x_{0}\right))+noi(i,j,t)$$
  Namely,
  $$\;y_{i}(t+1)=(1-u_{ij})y_{i}(t)+u_{ij}\left(\varepsilon^{j}\left(x_{0}\right)-\varepsilon^{i}\left(x_{0}\right)\right)+noi(i,j,t)=(1-u_{ij})y_{i}(t)+u_{ij}y_{j}(t)+noi(i,j,t)$$
  □

**Lemma** **A6.** *Under* $M_{1}$, $\forall x(0)\in {\left[0,1\right]}^{n}$, $\varepsilon \left(x_{0}\right)$ *is a stable stochastic fixed point of* $\left({\left[0,1\right]}^{n},\Omega ,F\right)$.

**Proof**.  For stochastic fixed point 0, given w∈Ω arbitrarily and considering $V(y)=\sum_{i}\left|y_{i}\right|,\forall t\in N$, we know that

(1) If ∀i,j, $Inter_{ij}\left(w_{t}\right)=0$, then ΔV(y(t))=0
(2) If $\exists i,j,Inter_{ij}(w_{t})\neq 0$, from Lemma A5, we know
  $$\begin{array}{l} \Delta V\left(y(t),w_{t}\right)=V(y(t+1))-V(y(t))\\ =\sum_{\left(i,j)\in E(t\right)}\left[\left|\left(1-u_{ij})y_{i}(t)+u_{ij}y_{j}(t)+u_{ij}\left(\varepsilon^{j}\left(x_{0}\right)-\varepsilon^{i}\left(x_{0}\right)\right)+noi(i,j\right)\right|-\left|y_{j}(t)\right|\right]\\ +\sum_{\left(i,j)\in E(t\right)}\left[\left|\left(1-u_{ij})y_{j}(t)+u_{ij}y_{i}(t)+u_{ij}\left(\varepsilon^{i}\left(x_{0}\right)-\varepsilon^{j}\left(x_{0}\right)\right)-noi(i,j\right)\right|-\left|y_{j}(t)\right|\right]\\ ⩽\sum_{\left(i,j)\in E(t\right)}\left[\left(1-u_{ij})\left|y_{i}(t)\right|+\left|u_{ij}y_{j}(t)+u_{ij}\left(\varepsilon^{j}\left(x_{0}\right)-\varepsilon^{i}\left(x_{0}\right)\right)+noi\left(i,j\right)\right||-|y_{i}(t)\right|\right]\\ +\sum_{\left(i,j)\in E(t\right)}\left[(1-u_{ij})\left|y_{j}(t)\right|+\left|u_{ij}y_{i}(t)+u_{ij}\left(\varepsilon^{i}\left(x_{0}\right)-\varepsilon^{j}\left(x_{0}\right)\right)-noi(i,j)\right|-\left|y_{i}(t)\right|\right]\\ =\sum -u_{ij}\left|y_{i}(t)\right|+u_{ij}\left|y_{j}(t)+\left(\varepsilon^{j}\left(x_{0}\right)-\varepsilon^{i}\left(x_{0}\right)\right)+\frac{noi\left(i,j\right)}{u_{ij}}\right|\\ +\sum -u_{ij}\left|y_{j}(t)\right|+u_{ij}\left|y_{i}(t)+\left(\varepsilon^{i}\left(x_{0}\right)-\varepsilon^{j}\left(x_{0}\right)\right)-\frac{noi(i,j)}{u_{ij}}\right|\\ =u_{ij}\sum \left(\left|y_{j}(t)\right|-\left|y_{i}(t)\right|\right)+u_{ij}\sum \left(\left|y_{i}(t)\right|-\left|y_{j}(t)\right|\right)\\ =0 \end{array}$$

This shows ΔV is negative constant. Arising from the stochastic Lyapunov’s theorem, $y\left(t\right)$ is stable at O, thus $x\left(t\right)$ is stable at $\varepsilon \left(x_{0}\right)$. □

**Lemma** **A7.** *For* $M_{1}$, $\forall x(0)\in {\left[0,1\right]}^{n}$*, If* |k|<u<1−|k|, $\varepsilon \left(x_{0}\right)$ *is a stochastic global attractor of* $\;\left({\left[0,1\right]}^{n},\Omega ,F\right)$.

**Proof.**  
**Part A: Simplification**
Arbitrarily given w∈Ω, ∀t∈N, let $\mathrm{maxmin}ij=E_{i}x_{i}(t)-\frac{x_{i}(t)+x_{j}(t)}{2}$, and given $x\left(0\right)\in {\left[0,1\right]}^{n}$

(1) if $E(t)=\phi ,\Delta V\left(y,w_{t}\right)=0$
(2) if E(t)≠ϕ, $\Delta V\left(y,w_{t}\right)$

$$\begin{array}{l} =\left[\sum_{i\in u(t)}\left|\left(1-u_{ij})y_{i}(t)+u_{ij}y_{j}(t)+u_{ij}\left(\varepsilon^{j}\left(x_{0}\right)-\varepsilon^{i}\left(x_{0}\right)\right)+noi(i,j\right)\right|+\right.\\ \left.\sum_{j\in v(t)}\left|\left(1-u_{ij})y_{j}(t)+u_{ij}y_{i}(t)+u_{ij}\left(\varepsilon^{i}\left(x_{0}\right)-\varepsilon^{j}\left(x_{0}\right)\right)-noi(i,j\right)\right|\right]-\sum_{V(t)}\left|y_{k}(t)\right|\\ =\sum \left|\left(1-u_{ij})y_{i}(t)+u_{ij}y_{j}(t)+noi(i,j\right)\right|-\left|y_{i}(t)\right|+\sum \left|\left(1-u_{ij})y_{j}(t)+u_{ij}y_{i}(t)-noi(i,j\right)\right|-\left|y_{j}(t)\right|\\ =\sum \left|(1-u_{ij})y_{i}(t)+u_{ij}y_{j}(t)+k\left(y_{i}(t)-y_{j}(t)\right)\max\min\mathrm{ij}\right|-\left|y_{i}(t)\right|\\ +\sum \left|(1-u_{ij})y_{j}(t)+u_{ij}y_{i}(t)+k\left(y_{j}(t)-y_{i}(t)\right)\max\mathrm{minij}\right|-\left|y_{j}(t)\right|\\ =\sum \left|\left(1-u_{ij}+k\mathrm{maxminij})y_{i}(t)+(u_{ij}-k\max\min ij)y_{j}(t\right)\right|-\left|y_{i}(t)\right|\\ +\sum \left|\left(1-u_{ij}+k\mathrm{maxmin}ij)y_{j}(t)+(u_{ij}-k\mathrm{maxmin}ij)y_{i}(t\right)\right|-\left|y_{j}(t)\right|\;\text{(or the same with above)}\\ =\sum \left|y_{i}(t)+(u_{ij}-k\max\min ij)\left(y_{j}(t)-y_{i}(t)\right)\right|-\left|y_{i}(t)\right|\\ +\sum \left|y_{j}(t)+(u_{ij}-k\mathrm{maxminij})\left(y_{i}(t)-y_{j}(t)\right)\right|-\left|y_{j}(t)\right| \end{array}$$

Denote $l_{ij}=u_{ij}-k\max\min ij$ and $l_{ij}^{*}=u_{ij}+k\max\min ij$, From $\left|k|<u_{ij}<1-|k\right|$,

$$\begin{array}{l} ①1-u_{ij}>|k|>-k\max\min ij②u_{ij}>|k|>k\mathrm{kaxminij}\\ ③1-u_{ij}>|k|>k\max\min ij④u_{ij}>|k|>-k\max\min ij \end{array}$$

Then, we have $l_{ij}>0$ and $l_{ij}{}^{*}>0$, thus

$$\Delta (y(t),w)=\sum_{\left(ij)\in E(t\right)}\left\{\left[\left|\left(1-l_{ij}\right)y_{i}(t)+l_{ij}y_{j}(t)\right|+|\left(1-l_{ij}\right)y_{j}\left(t\right)+l_{ij}y_{i}\left(t\right)|\right]-\left[\left|y_{i}(t)\right|+\left|y_{j}(t)\right|\right]\right.$$

**Part B: Scaling**
Given w,t satisfy (2), if $0<l_{ij}<1$, we know: when ∀i,j, $y_{i}\left(t\right)\cdot y_{j}\left(t\right)⩾0$, ΔV(y,w)=0 when ∃i,j, $y_{i}\left(t\right)\cdot y_{j}(t)<0$, ΔV(y,w)<0.
**Part C: Lower bound**
Given w∈Ω arbitrarily, given j satisfying $d_{j}=1$ and $\mathrm{degree}\;\left(v_{j}\right)=\left|V\right|-1$

(1) V(t)⩾0, obviously, 0 is the lower bound of V(t), thus P{infV(t)≥0}=1
(2) If $P\{\underset{t}{\inf}V\left(t\right)=0\}<1$, namely $\exists K,P\left(0<K=\underset{t}{\inf}V(t)\right)>0$, then ∃A⊆Ω, P(A)>0, ∀w∈A, we have V(t)⩾K and ∀ε>0, $\exists t^{*},V\left(t^{*}\right)<K+\varepsilon$. From the non-descending feature of  V(t), we know $\forall t>t^{*}$, V(t)<K+ε. Given the N enough large, given $\varepsilon =\frac{1}{n}K$ enough small, when $t=t^{*}$, $V\left(t^{*},W\right)\neq 0$, there are two conditions:

① Let $Q=\left\{w:y_{j}\left(t^{*}\right)=0\right\}$, $M≜\left\{w:\exists i\in I,\mathrm{Interij}\;\left(t^{*}\right)=1,i\;is\;not\;in\;synchronization\;state\right\}$, $N≜\left\{w:\exists i\in I,\mathrm{Interij}\;\left(t^{*}\right)=1,i\;is\;in\;synchronization\;state\;\right\},P{\left(M\cup N\right)}^{c}=0$, we can, respectively, discuss by A and B below.
  A. To consider $M,P\left(Q\cap \left\{w:\forall k>0,\underset{t}{\mathrm{Inf}}V(t)<k\right\}\cap M\right)=P(Q\cap M)$, which is because: for $w_{t}$ satisfying ${\mathrm{Inter}}_{\mathrm{ij}}\;\left(w_{t}\right)=1$, if i is not in synchronization state, $y_{j}\left(t^{*}+1\right)$ will be not in synchronization state, and $\forall t>t^{*}$, ∀k, $\;\mathrm{Interij}\left(w_{t}\right)=1$ and $x_{k}(t)\cdot x_{j}(t)⩾0$, so $x_{j}(t+1)\neq E_{i}x_{i}(0)$, namely, $y_{j}(t+1)\neq 0$.
  Therefore,
  $$\begin{array}{l} P(Q\cap M)=P\left(Q\cap M\cap \{w:\exists t>t^{*},{\mathrm{Inter}}_{\mathrm{mj}}\left(w_{t}\right)=1\;\mathrm{and}\left.x_{m}(t)\cdot x_{j}(t)<0\right\}\right)\\ ⩽P(Q\cap M\cap \{V(t+k)<V(t+k)-\delta \})⩽P(Q\cap M\cap \{\forall k>0,\underset{t}{\mathrm{In}f}V(t)<k\})\\ \leq P(Q\cap M) \end{array}$$
  Thus, we can take appropriate N and ε, which can satisfy the below conclusion:
  $$P\left(Q\cap M\cap \left\{w:\underset{t}{\mathrm{Inf}}V(t)=K>0\right\}\right)=P(Q\cap M)-P(Q\cap M)=0$$
  B. To consider N, further define the set that is not in synchronization state $NS(t)=\left\{k:y_{k}(t)\neq 0\right\}\left(t>t^{*}\right)$.
  B_1_. Obviously, $\left.P(Q\cap N\cap \{K>0\}\cap \{\exists t>t^{*},Ns(t)=\phi )\}\right)=0$.
  B_2_. $P(Q\cap N\cap \{k>0\})=0+P\left(Q\cap N\cap \{k>0\}\cap \left\{\forall t>t^{*},NS(t)\neq \phi \right\}\right)$
  $\leq P\left(Q\cap \{k>0\}\cap N\cap \left\{\exists t^{\prime }>t^{*},k\in NS\left(t^{\prime }\right)\right.\right.,Interkj\left(t^{\prime }\right)=1,y_{k}\left(t^{\prime }\right)y_{j}\left(t^{\prime }\right)<0\}\}$
  $\leq P\left(Q\cap \{k>0\}\cap \left\{\underset{t}{\mathrm{Int}}V(t)<K\right\}\right)=0$
  Therefore, from A and B, we know that
  {P(Q∩{K>0})=P(Q∩N∩{K>0})+P(Q∩M∩{K>0})+0=0
② For $w\in Q^{C}$, namely, for j, $y_{j}\left(t^{*}\right)\neq 0$, which is similar to A in ①, then $P\left(Q^{C}\cap \{K>0\}\right)=0$.
  To synthesize ①②, P({K>0})=0, which is a contradiction, thus $P\left(\underset{t}{\inf}V(t)=0\right)=1$.

**Part D: QED**
Therefore, to synthesize Parts A/B/C, based on the stochastic Lasalle’s theorem, O is the stochastic global attractor of $y\left(t\right)$. Then from the arbitrary features of x(0) and w, $\varepsilon \left(x_{0}\right)$ is the stochastic global attractor of $\left({\left[0,1\right]}^{n},\Omega ,F\right)$. □

**Lemma** **A8.** *If* $\left({\left[0,1\right]}^{n},\Omega ,F\right)$ *has a stochastic global attractor* $\varepsilon \left(x_{0}\right)$, $x\left(t\right)$ *will have quasi-consensus*.

**Proof.**  Because $\varepsilon \left(x_{0}\right)$ is a stochastic global attractor, we can obtain A satisfying P(A) = 1, and given w∈A arbitrarily, since $\varepsilon \left(x_{0}\right)=\left({\bar{x}}_{0}\cdots \cdots ,{\bar{x}}_{0}\right)$, we have ∀ε>0,∃N,t>N $\left\|x(t)-\varepsilon \left(x_{0}\right)\right\|<\varepsilon$. From the convergent feature of norm $\left\|\cdot \right\|$, $\forall i,x_{i}(t)\to E^{\left(i\right)}\left(x_{0}\right)={\bar{x}}_{0}$ holds. Therefore, ∀l,k∈I, from $x_{l}(t)\to {\bar{x}}_{0}$ and $x_{k}(t)\to {\bar{x}}_{0}$, we obtain ∀ε>0,∀l,k, for $\frac{\varepsilon }{2},\exists T_{l},T_{k},\forall t>T_{lk}=\max\left(T_{l},T_{k}\right)$, $\left|x_{l}(t)-{\bar{x}}_{0}\right|<\frac{\varepsilon }{2}$ and $\left|{\bar{x}}_{0}-x_{l}(t)\right|<\frac{\varepsilon }{2}$, so we know that $|x_{l}(t)-x_{k}(t)|<\frac{\varepsilon }{2}+\frac{\varepsilon }{2}=\varepsilon$, then $\forall \varepsilon >0,\exists N,t>N,d_{v}(t)=\underset{k,l}{\sup}\left|x_{k}(t)-x_{l}(t)\right|<\varepsilon$, and then $dv=\underset{t}{\lim}\sup d_{v}(t)=0$, $x\left(t\right)$ will have quasi-consensus arising from the arbitrary choice of w. □

**Theorem** **A6.** *For* $M_{1}$, $\left({\left[0,1\right]}^{n},\Omega ,F\right)$ *has a stable quasi-consensus*.

**Proof.** 

① From Lemmas A6 and A7, the translation system $y\left(t\right)$ has a stable stochastic fixed point and stochastic global attractor o. Therefore, $\exists P(A)=1,\forall w\in A,t\to \infty ,x(t)-\bar{x(0)}\to 0$, $x(t)\to \bar{x(0)}$, so $\bar{x(0)}$ is a stable stochastic fixed point and stochastic global attractor of $x\left(t\right)$.
② From Lemma A8, we know that stochastic global attraction can lead to a quasi-consensus. Further, based on the stability of $\bar{x(0)}$, $\left({\left[0,1\right]}^{n},\Omega ,F\right)$ has a stable quasi-consensus. □

### Appendix B.2. The Proof of Theorem 2

**Proposition** **A2.** $\forall x_{0}\in {\left[0,1\right]}^{n}$*, under the alternative rule in the definition,* $A_{2}=\left\{w:{\mathrm{Inter}}_{\mathrm{ij}}(w_{t})\cdot {\mathrm{II}}_{\left(0,d\right)}\left(\left|x_{i}(t)-x_{j}(t)\right|\right)=1(P1)\right\}$*, and under the original rule in the definition,* $A_{1}=\{w{\;:\;\mathrm{Inter}}_{\mathrm{ij}}\;\left(w_{t}\right)=1(P2)\}$*, then* $A_{2}=A_{1}$.

**Proof.**  Namely, we need to prove “$P_{1}\Leftrightarrow P_{2}$” “⇒” If $P_{1}$ holds, we have ${\;\mathrm{Inter}}_{\mathrm{ij}}\;\left(w_{t}\right)=1$, $Y_{2}$ holds. “⇐” If $P_{2}$ holds and $P_{1}$ does not hold, namely ${\;\mathrm{inter}}_{\mathrm{ij}\;}\left(w_{t}\right)=1$ and $\left|x_{i}(t)-x_{j}(t)\right|>d$, from the ② of rule 2, we know: when $\forall i,{\mathrm{sign}}_{i}\left(w^{\prime }\right)={\mathrm{sign}}_{i}(w)$, $P\left(\left\{w^{\prime }\right\}\cap \left\{\left|x_{i}(t)-x_{j}(t)\right|<d\right\}\right)$ $=P\left(\{w\}\cap \left\{\left|x_{i}(t)-x_{j}(t)\right|<d\right\}\right)$, then

$$0\neq P\left(\left\{w:w_{ij}>0\right\}\cap \left\{\left|x_{i}(t)-x_{j}(t)\right|>d\right\}\right)=\sum P\left(\{w\}\cap \left\{\left|x_{i}(t)-x_{j}(t)\right|<d\right\}\right)$$

Thus, $\exists w^{*},P\left(\left\{w^{*}\right\}\cap \left\{\left|x_{i}(t)-x_{j}(t)\right|<d\right\}\right)\neq 0$, so $P\left(\{w\}\cap \left\{\left|x_{i}(t)-x_{j}(t)\right|<d\right\}\right)\neq 0$, which is a contradiction, so $P_{1}$ holds. □

**Remark** **A17.**
*The equivalence of the original rule and the alternative rule is known from the proposition. Therefore, Theorem 2 can be proved below under the alternative rule.*

**Proposition** **A3.** $P\left(A_{1}\right)=1,P\left(A_{2}\right)=1$*, then* $P\left(A_{1}\cap A_{2}\right)=1$.

**Proof.** It obviously holds on the basis of basic probability theory. □

**Lemma** **A9.** *For* $M_{2}$*, when* $u⩽\frac{1}{2}$, $P\{w:if\underset{i\in V\left(t\right)}{\min}x_{i}(t)\mathrm{interact}\;\mathrm{at}\;\mathrm{the}\;\mathrm{time}t,\mathrm{then}$ $\underset{i\in V\left(t+1\right)}{\min}x_{i}(t+1)-\underset{i\in V\left(t\right)}{\min}x_{i}(t)⩾u(x_{\sigma_{t}}{}_{\left(2\right)}^{}\left(t\right)-x_{\sigma_{t}}{}_{\left(1\right)}^{}\left(t\right))\}=1$.

**Proof.** 

I. Given $x_{0}\in {\left[0,1\right]}^{n}$ arbitrarily, Let $\sigma_{t}:\{1\cdots n\}\to \{1\cdots n\}$ be a permutation of V(t), which sorts i in V(t) from small to large by the value of $x_{i}\left(t\right)$, and can be denoted as $\sigma_{t}(x(t))={\left(x_{\sigma (i)}^{}\left(t\right)\right)}_{t=1}^{n}$. Given t∈Nandi,j∈V(t), we obtain: ∃A⊆Ω, PA=1, ∀w∈A, for i that interacts with the other agent at the time t; the equation reflecting its dynamics can be briefly written as $x_{i}(t+1)=(1-u)x_{i}(t)+u\cdot \frac{1}{N_{i}(t)}\sum_{k\in N_{i}(t)}x_{k}(t)$. Thus, we know $x_{\sigma_{t}\left(1\right)}^{}(t+1)-x_{\sigma_{t}(1)}^{}(t)⩾(1-u)x_{\sigma_{t}(1)}^{}(t)+ux_{\sigma_{t}(2)}^{}(t)-x_{\sigma_{t}(1)}^{}(t)=u\left(x_{\sigma_{t}(2)}^{}(t)-x_{\sigma_{t}(1)}^{}(t)\right)$ and $x_{\sigma_{t+1}(1)}^{}(t+1)⩾\min\left[x_{\sigma_{t}(1)}^{}(t+1),\frac{1}{2}\left(x_{\sigma_{t}(1)}^{}(t)+x_{\sigma_{t}(2)}^{}(t)\right)\right]$, so the inequality holds below:
  $\underset{i\in V(t)}{\min}x_{i}(t+1)-\underset{i\in V(t)}{\min}x_{i}(t)=x_{\sigma_{t+1}^{}\left(1\right)}\left(t+1\right)-x_{\sigma_{t}\left(1\right)}\left(t\right)⩾\min\left[u\left(x_{\sigma_{t}{}^{\left(2\right)}}^{}(t)-x_{\sigma_{t}{}^{\left(1\right)}}^{}(t)\right)\right.,\left.\frac{1}{2}\left(x_{\sigma_{t}{}^{\left(2\right)}}^{}(t)-x_{\sigma_{t}{}^{\left(1\right)}}^{}(t)\right)\right]$ $=u\left(x_{\sigma_{t}{}^{\left(2\right)}}^{}(t)-x_{\sigma_{t}{}^{\left(1\right)}}^{}(t)\right)$, written as Inq1 in the following part.
II. Given the set A same as above, ∀w∈A, we consider $V\left(t+1\right)$ and know:
  (1) When $\left.V^{c}(t)=\{k\in V:B\left(x_{k}(t)\right),d)=\phi \right\}$ and let $B(t)=\{k:x_{k}(t)⩽\underset{i\in V(t)}{\min}x_{i}(t)\;\mathrm{or}\;x_{k}(t)⩾$
    $\underset{i\in V(t)}{\max}x_{i}(t)\left(P_{2}(t)\right)\}$, we obtain $\forall x_{k}(t)\in V^{c}(t)\cap B(t)$, $x_{k}(t+1)=x_{k}(t)$ and $\min x_{i}(t)/\max x_{i}(t)$ is non-decreasing/non-increasing, then $x_{k}(t+1)\in V^{c}(t+1)\cap B^{c}(t+1)$ and we know that $V^{c}(t)\cap B(t)\subseteq V^{c}(t+1)$
  (2) $\forall k\in V^{c}(t)\cap B^{C}(t)$, namely $B\left(x_{k}(t),d\right)=\phi$ and $\underset{i\in V(t)}{\min}x_{i}(t)<x_{k}(t)<\underset{i\in V(t)}{\max}x_{i}(t),\;$ suppose only one k that satisfies this condition (which can be generalized to n), from the non-existence of $x_{l}(t)\in B\left(x_{k}(t),d\right)$, we know: there is a partitioning of $V\left(t\right)$, namely $V_{1}(t)\cap V_{2}(t)=\phi$ and $V_{1}(t)\cup V_{2}(t)=V(t)$, which can satisfy $x_{k}(t)-\underset{i\in V_{1}(t)}{\max}x_{i}(t)>d$ and $\underset{j\in V_{2}(t)}{\min}x_{j}(t)-x_{k}(t)>d$. Thus, $\forall m\in V_{1}(t),n\in V_{2}(t)$, $\;\mathrm{II}\;(-\infty ,d)\left(\left|x_{m}(t)-x_{n}(t)\right|\right)=0$ and $x_{k}(t+1)=x_{k}(t)$, we have
    ① (fixed agents) $\forall p\notin V_{1}(t)\cup V_{2}(t),p\neq k,x_{p}(t+1)=x_{p}(t)\notin B\left(x_{k}(t+1),d\right)$;
    ② (Activable agents) from $d\left(V_{1}(t),V_{2}(t)\right)>d$, we know $\underset{m\in V_{1}(t)}{\max}x_{m}(t+1)⩽\underset{m\in V_{1}(t)}{\max}x_{m}(t)$ and $\underset{n\in V_{1}(t)}{\min}x_{n}(t+1)⩾\underset{n\in V_{1}(t)}{\min}x_{n}(t)$ holds, and $\left.\forall p\in V_{1}(t)\cup V_{2}(t),x_{p}(t+1)\notin B\left(x_{k}(t+1)\right),d\right)$, so k∉V(t+1). Therefore, $k\in V^{c}(t+1)$, namely $V^{c}(t)\cap B^{c}(t)\subseteq V^{c}(t+1)$.

From (1) and (2) of II, given A, we obtain the below conclusion: $\forall w\in A,V^{c}(t)=\left(V^{c}(t)\cap B(t)\right)$ $\cup \left(V^{c}(t)\cap B^{c}(t)\right)\subseteq V^{c}(t+1)$, namely V(t+1)⊆V(t), thus 1⩾P{w:V(t+1)≤V(t)}⩾P(A)=1, so the probability is equal to 1. Then, from I and II, we know $\forall x_{0}\in {\left[0,1\right]}^{n},\forall w\in A$, since $\underset{i\in V(t+1)}{\min}x_{i}(t+1)\geq \underset{i\in V(t)}{\min}x_{i}(t+1)$, for the condition which min x_i_(t) participates the interaction, we have

$$P\left\{w:if\underset{i\in V\left(t\right)}{\min}x_{i}(t)interact\;at\;the\;time\;t,\underset{i\in V\left(t+1\right)}{\min}x_{i}(t+1)-\underset{i\in V\left(t\right)}{\min}x_{i}(t)⩾u(x_{\sigma_{t}}{}_{\left(2\right)}^{}\left(t\right)-x_{\sigma_{t}}{}_{\left(1\right)}^{}\left(t\right))\right\}=1$$

. □

**Lemma** **A10.** *When* u≤1/2, $P(\exists x_{0}\in {\left[0,1\right]}^{n},t\to \infty ,x(t)\to x_{0})=1$.

**Proof**.  Given $x\left(0\right)\in {\left[0,1\right]}^{n}$, w∈Ω arbitrarily, let $E^{*}(t)=\left\{x_{i}(t):\exists x_{j}(t),0\left.<\left|x_{i}(t)-x_{j}(t)\right|<d\right\}\right.$. Obviously, $E^{*}(t)=\phi$ or $\left|E^{*}(t)\right|⩾2$.

1. x_i_(t) Under $E^{*}(t)$ (or above E*(t)) will be convergent.
  $\forall x_{l}(t)<\min E^{*}(t),x_{k}(t)>\max E^{*}(t)$, we have $x_{l}(t)-\min E^{*}(t)<-d,$ $x_{k}(t)-\max E^{*}(t)>d$.
  From Lemma A9, we deduce that $\min E^{*}(t+1)⩾\min E^{*}(t)$ and $\max E^{*}(t+1)⩽\max E^{*}(t)$.
  Thus, $x_{l}(t+1)-\min E^{*}(t+1)<-d$ and $x_{k}(t+1)-\max E^{*}(t+1)>d$, so $x_{l}(t+1)\notin E^{*}(t+1)$ and $x_{k}(t+1)\notin E^{*}(t+1)$. Then, it is known by induction that $x_{l}(t+k),x_{k}(t+k)\notin E^{*}(t+k)$. Therefore, $\exists T,\forall t^{\prime }>T$ and t>T, $\forall x_{k}(T)\leq \min E^{*}(T)$ or $x_{k}(T)⩾\max E^{*}(T)$, $x_{k}\left(t^{\prime }\right)=x_{k}(t)$. Namely, $x_{k}(t)\to x_{k}$, and we can obtain the equation:
  $$P\left(\left\{\exists t,x_{k}(t)\notin \left[\min E^{*}(t),\max E^{*}(t)\right]\right\}\cap \left.\left\{x_{n}(t)\to x_{k}\right\}\right)=P\left(\exists t,x_{k}(t)\notin \left[\min E^{*}\left(A\right),\max E^{*}(t)\right]\right)\right.$$
2. x_i_(t) in E*(t) will be convergent $\left(namely,x_{k}(t)\in \left[\min E^{*}(t),\max E^{*}(t)\right]\right)$, given ω∈Ω arbitrarily.
  (1) If $\exists t,E^{*}(t)=\phi$, then ∀i/j, $\left|x_{i}(t)-x_{j}(t)\right|⩾d$, so $x_{i}\left(t+1\right)=x_{i}\left(t\right)$ keep the feature, $E^{*}\left(t+1\right)=\phi$. By induction, we know $\forall t^{\prime }>t,E^{*}(t^{\prime })=\phi$, thus $x\left(t\right)\to x_{0}$ holds, and
    $$\begin{array}{l} P\left(\left\{\forall t,x_{k}(t)\in \left[\min E_{}^{*}\left(t\right),\max E^{*}\left(t\right)\right]\right\}\right.\cap \left.\left\{\exists t:E^{*}(t)=\emptyset \right\}\cap \{x(t)\to x(0)\}\right)\\ =P(\left\{\forall t,x_{k}(t)\in \left[\min E^{*}(t),\max E^{*}(t)\right]\right\}\left.\cap \left\{\exists t:E^{*}(t)=\phi \right\}\right) \end{array}$$
  (2) if $\forall t,E^{*}(t)\neq \phi$, given t, let $\min1(t)=\underset{i\in V(t)}{\min}x_{i}(t)$ (also equal to $\min E^{*}(t)$). From Lemma A9 and ω∈A (A is the set given in Lemma A9), we know {min1(t):t∈N} is bounded and non-decreasing, thus t→∞, $\min1(t)\to x_{1}$.

① To consider the limit of min1(t), written as $x_{1}$,
  By the convergence of min1(t), ∀δ>0, ∃T,∀t>T, $\;x_{1}-\min1(t)<\delta$. From $\min(t)\in E^{*}(t)$, we know B(min1(t),d)≠ϕ.
  I. Suppose $\exists A_{1}\subseteq A$ and $P\left(A_{1}\right)>0$, $P\left(w\in A_{1}:\exists \delta >0,\forall T>0,\exists t>T\right.$ (written as $T_{1}\cdots T_{n}\cdots$), $x_{j}(t)\in B(\min1(t),d)$ and $\left.x_{j}(t)-\min(t)>\delta \right)>0$.
  For the condition that “$x_{j}(t\in B(\min1(t)d)$ and $x_{j}(t)-\min1(t)>\delta$”, we know: given w, given $T=T_{0}$, firstly, there is a $t=T_{1}>T_{0}$ satisfying the condition; then let $T=T_{1}$, there is a $t=T_{2}>T_{1}$ satisfying the condition… Based on this procedure, for w in A, there are countable $T_{i}\left(i\in N\right)$ satisfying the condition.
  II. Based on the conclusion of I, let $up(t)=\left\{x_{i}(t)\in E^{*}(t):\min(t)\leq x_{i}(t)\leq x_{1}\right\}$, which is obviously not equal to ϕ. Given the apagogical hypothesis is $P(M)≜P\left\{\exists \delta >0,\forall T_{i},\exists t=T_{i+1}>T_{i},\exists x_{j}(t)\in B(\min1(t),d),\mathrm{then}\left|x_{j}\left(t\right)-\min1(t)|\right.>\delta \right\}$, we know the below conclusion:
  $$P\left(\sum_{up(t)}x_{i}(t)⩾|up(t)|\left(x_{1}+d\right)\right)⩾P(w:\exists I\subseteq N,\;\left|I\right|=N,\exists \delta >0,\underset{t_{i}\in I}{\cap }\left\{\mathrm{Inter}\left(\min1\left(t_{i}\right),x_{\left(j\right)},\left(t_{i}\right)\right)\left(w_{t_{i}}\right)=1\right\}$$
  and $\left.\exists (j),x_{\left(j\right)}\left(t_{i}\right)-\min1\left(t_{i}\right)>\delta \right)⩾p(M)>0$.
  For the proof of the first equal sign, we take $t>T_{0}$ and fix the $up\left(t\right)$, given $\min1(t)=x_{k}{}_{{}_{t}}(t)\in up(t)$, if $x_{i}(t)\in up(t),\mathrm{Inter}\left(x_{k_{t}}(t),x_{i}(t)\right)=1$, $\sum_{up(t)}x_{i}(t)$ will not change; if $x_{j}\left(t\right)\notin up\left(t\right)$, $\;\mathrm{Inter}\;\left(x_{k_{t}}(t),x_{j}(t)\right)=1$, $\sum x_{i}(t)$ will increase more than l (from Lemma A9). For the proof of the second inequality, from I, the cardinality of ${\left\{T_{i}\right\}}_{i=1}^{\infty }$ is same with N and $P\left({\mathrm{inter}}_{ij}\left(T_{i}\right)\cap \left\{x_{1}\left(T_{i}\right)-x_{j}\left(T_{i}\right)<d\right\}\right)>0$, thus t→∞, $|I|=N,\cap \left\{\mathrm{inter}\left(\;\min1(\mathrm{ti}),\;x_{\left(j\right)}\left(t_{i}\right)\right)\left(w_{t_{i}}\right)=1\right\}$ and $\left|x_{j}(t)-\min\left(t_{i}\right)\right|>\delta$, the inequality holds. From this, $P\left[\exists k^{\prime },E(up(t+k))⩾E(up(t))+k^{\prime }\cdot u\delta ⩾n\left(x_{1}+d\right)\right]>0,$ and E represents the sum of elements in up(t), which is a contradiction. Therefore, $\forall A_{1}\subseteq A$ and $P\left(A_{1}\right)>0,P\left(w\in A_{1}:\forall \delta >0,\exists T>0,\forall t>T,x_{j}(t)\in B(\min1(t),d\right)\Rightarrow x_{j}(t)\in$$B(\min1(t),\delta ))=P\left(A_{1}\right)$. Furthermore, Let $A_{1}=A$, $P\{w:\forall \delta >0,\exists T>0,\forall t>T,x_{j}(t)\in$$B(\min1(t),d)\Rightarrow x_{j}\left(t\right)\in B\left(\min1(t),\delta \right)\}=1$
  III, From II, the probability of “∀δ>0, $\exists T_{1}=T(\delta ),\forall t>T_{1},\forall x_{j}(t)\in B\left(\min1\left(T_{1}\right),d\right)$, $\left|x_{j}(t)-\min1(T_{1})\right|<\delta$” is equal to 1. Furthermore, given w, we define $\min2(t)=\underset{\left\{x_{i}(t)\right\}\backslash B(\min1(t),d)}{\min}x_{i}(t)$, and use the apagogical arguments. For w in measure P in II, $\exists m>0,P\left(K_{2}\right)≜P(w:\forall t,\exists x_{i}(t)\in B(\min1(t),d)$, $\left.\min2(t)\in B\left(x_{i}(t),d\right)\right)>m$, we take $\delta =\frac{d}{2}$, and find $T\left(\frac{d}{2}\right)$ based on II satisfying $P\{w:t>T\left(\frac{d}{2}\right),x_{j}(t)\in B\left(\min1(t),d\right),$ $\mathrm{then}\;x_{j}(t)\in B(\min1(t),d/2)\}$, then we take $t>T\left(\delta \right)$ and consider the apagogical arguments hypothesis, knowing some points below:
  A. $P\left(w\in K_{2}:t\to \infty ,\exists {\left\{t_{i}\right\}}_{i=1}^{\infty },\underset{x\left(t\right)\in B\left(\min1(t),d\right)}{\cap }\underset{t_{1}\cdots t_{n}}{\cap }\left\{\mathrm{Inter}\left(x_{k}(t),\min2(t)\right)\left(w_{t_{i}}\right)=1\right\}\right)=P\left(K_{2}\right)$
  B. Given w, if $\mathrm{Inter}\left(x_{k}(t),\min2(t)\right)=1$, $\sum_{i\in B\left(\min1(t),d\right)}x_{i}(t+1)⩾\sum x_{i}(t)+u\cdot \frac{d}{2}$. Thus, $P\left(\left\{w:t\to \infty ,\sum x_{i}(t)\to \infty \;\right\}\right)⩾m>0$, which is a contradiction, so P(∃t,min2(t) does not exist, or $\left.\exists T,\min2(t)⩾\underset{B(\min1(t),d)}{\max}x_{i}(t)+d\right)=1$, and then we obtain $P\left(\exists T,t>T,\forall x_{k}(t)\in \right.\left.B(\min1(t),d),\mathrm{Inter}\left(\min2(t),x_{k}(t)\right)=0\right\}=1$.
② To consider mini(t),1<i≤w.
  When i>1, we define $\min i\left(t\right)$ like min2(t) in ①, and view $\min i\left(t\right)$ as min1(t),mini+1(t) as $\min2\left(t\right)$. After discussing like above process, we know: $\forall i\in \{2\cdots w\},\exists T_{i},\forall t>T_{i}$, $B\left(\min i\left(t\right),d\right)\cap B\left(\mathrm{mini}\;+1(t),d\right)=\phi$, then $P(\{\exists T,t>T,\mathrm{Interij}(w_{t})=1\})=0(x_{i}(t)\in B(\mathrm{mini}(t),d),x_{j}(t)\in B\left(\min i+1(t),d\right))$. Furthermore, we have $P(\exists \max\left(T_{1}\ldots T_{n}\right),\;\forall t>\max\left(T_{1}\cdots T_{n}\right),\mathrm{Inter}ij(w_{t})=1)=0$, which satisfies
  $x_{i}\left(t\right)\in B\left(\min\left(l+1,d\right)\right),x_{j}\left(t\right)\in B\left(\min k\left(t\right),d\right)$ and k≠l. Because $\left|V\right|<\infty$, ∃ i (just finite numbers of i, which written as m) $\forall x_{p}(t)\in [\mathrm{mini}(t),\min i(t)+d],x_{p}(t)\to x_{i}$. After ordering, we prove that $x(t)\to \left(x_{1}\cdots x_{1}\ldots x_{m}\cdots x_{m}\right)$.
  In conclusion, when x(0) belongs to [0, 1]^n^ and w belongs to A, $t\to \infty ,\exists x_{0},x(t)\to x_{0},\;$ we have $P(\exists x_{0}\in {\left[0,1\right]}^{n},t\to \infty ,x(t)\to x_{0})=P(A)=1$. □

**Theorem** **A7.** *For M2, when* $u⩽\frac{1}{2},n=[1/d]+1$, arbitrarily given a starting point  $x_{*}\in {\left[0,1\right]}^{n}$, $P\left(w:N\mathrm{consensus}\;\left(w,x_{0}\right)⩽n\right)=1$.

**Proof.**  Given $x_{0}$ arbitrarily, when $d>\frac{1}{n}$, $P\left(N{\;}_{\mathrm{consensus}}\;\left(w,x_{0}\right)>n\right)>0$, so ∃ A and P(A) > 0, ∀w∈A, there are $x_{i}(t)\cdots x_{n+1}(t)$ satisfying $x_{1}(t)\to x_{1},\cdots ,x_{n+1}(t)\to x_{n+1}$ and $x_{i}\neq x_{j}$. From Lemma A10, we know $P\left(x(t)\to x_{0}\right)=1$, and based on $d>\frac{1}{n}$ obtain $P(A\cap \left\{w:x(t)\to x_{0}\right\})>0$. Arbitrarily given w, if $\left|x_{i}-x_{j}\right|<d$, let $\varepsilon_{0}=d-\left|x_{i}-x_{j}\right|$, $\left|x_{i}(t)-x_{j}(t)\right|\leq \left|x_{i}(t)-x_{i}\right|+\left|x_{i}-x_{j}\right|$ $+\left|x_{i}-x_{j}(t)\right|=\left|x_{j}(t)-x_{i}\right|+\left|x_{j}(t)-x_{j}\right|+\left(d-\varepsilon_{0}\right)$. From $x_{i}(t)\to x_{i},x_{j}(t)\to x_{j}$, $\exists T_{1},T_{2}$ $\forall t>T=\max\left(T_{1},T_{2}\right)$, $\left|x_{i}(t)-x_{i}\right|<\frac{\varepsilon_{0}}{2}$ and $\left|x_{j}(t)-x_{j}\right|<\frac{\varepsilon_{0}}{2}$, so $\left|x_{i}\left(t\right)-x_{j}\left(t\right)\right|<d$. Therefore, $\forall t>T,x_{i}(t),x_{j}(t)\in E^{*}(t)$, namely “∃!T,t>T, $E^{*}\left(t\right)=\phi$ or $\left|x_{i}\left(t\right)-x_{j}\left(t\right)\right|>d$” (written as M), and based on the Lemma A10, $P\left(\exists T^{\prime },\forall t>T^{\prime },\;E^{*}(t)=\phi or\left|x_{i}(t)-x_{j}(t)\right|>d\right)=1$, so P(M)=0 is contradicted with $P(M)⩾P\left(A\cap \left\{w:x(t)\to x_{0}\right\}\right.$, so $P\left(N{\;}_{\mathrm{consensus}}\;\left(w,x_{0}\right)>n\right)=0$, and $\forall x_{0}$, $P\left(N{\;}_{\mathrm{consensus}}\;\left(w,x_{0}\right)⩽n\right)=1$. □

### Appendix B.3. The Proof of Theorem 3

**Lemma** **A11.** *For* $M_{3}$, $\exists \tilde{F}\in M_{n}(R),P\left\{w:\exists T,t>T,F\left(w_{t}\right)=\tilde{F}\right\}=1$.

**Proof.** From the remarks in definitions, the conclusion is obvious. □

**Lemma** **A12.** *If*$\tilde{F}\mathrm{is}aexpanding\;mapping(\exists \alpha >1,d(\tilde{F}x,\tilde{F}y)>\alpha d(x,y)),$ *then* $\tilde{F}$ *is an expansive mapping*.

**Proof.**  If $\tilde{F}$ is expanding, then, given $x_{1},x_{2}\in {\left[0,1\right]}^{n}$ arbitrarily, let e=1 ∃α>1, $d\left(\tilde{F}x_{1},\tilde{F}x_{2}\right)⩾\alpha \cdot d\left(x_{1},x_{2}\right)$. From induction, we know that when $n>N=\log_{\alpha }\frac{1}{d\left(x,x_{2}\right)}+1$, $d\left({\tilde{F}}^{n}x_{1},\;{\tilde{F}}^{n}x_{2}\right)⩾\alpha^{n}d\left(x_{1},x_{2}\right)>1=e,$ so $\tilde{F}$ is a expansive mapping. □

**Lemma** **A13.** *If ([0, 1]^n^, Ω,* $\tilde{F}$*) has* L−Y *chaos**, it will not have quasi-consensus.*

**Proof.** 

① For ([0, 1]^n^, Ω, $\tilde{F}$) has L−Y chaos, $\exists A\subseteq {\left[0,1\right]}^{n},\forall x,y\in A$, we obtain that $\underset{n}{\lim}\sup d\left({\tilde{F}}_{x}^{n},{\tilde{F}}_{y}^{n}\right)\neq \underset{n}{\lim\inf}d\left({\tilde{F}}^{n}x,{\tilde{F}}^{n}y\right)$, $\exists \left\{n_{ki}\right\},\left\{n_{kj}\right\}$, $\underset{n_{ki}}{\lim}d\left({\tilde{F}}_{}^{n}x,{\tilde{F}}_{}^{n}y\right)\neq$ $\underset{n_{kj}}{\lim}d\left({\tilde{F}}_{}^{n}x,{\tilde{F}}_{}^{n}y\right)$, so $\lim d\left({\tilde{F}}_{}^{n}x,{\tilde{F}}_{}^{n}y\right)$ does not exist.
② Suppose ([0, 1]^n^, Ω, $\tilde{F}$) has quasi-consensus, and it also has L-Y chaos, when n→∞, Let ${\tilde{F}}_{}^{n}x\to (a\cdots a),{\tilde{F}}_{}^{n}y\to (b\cdots b)$, From the continuity of the norm, we know that $\underset{n}{\lim}d\left({\tilde{F}}_{}^{n}x,{\tilde{F}}_{}^{n}y\right)=\underset{n}{\lim}\left\|{\tilde{F}}^{n}(x-y)\right\|=\left\|\underset{n}{\lim}{\tilde{F}}^{n}(x-y)\right\|$ $=\left\|\underset{n}{\lim}{\tilde{F}}_{}^{n}x-\underset{n}{\lim}{\tilde{F}}_{}^{n}y\right\|=||(a-b,$⋯,a−b)|| exists, which is contradicted with ①, so the hypothesis does not hold.
  Therefore, if ([0, 1]^n^, Ω, $\tilde{F}$) has L−Y chaos, it will not have quasi-consensus. □

**Lemma** **A14.** ∃k>0, $\tilde{F^{k}}$ *has 2-order shift invariant set. Namely,* $\left({\left[0,1\right]}^{n},{\tilde{F}}^{k}\right)$ *is topological conjugate with one-sided symbolic system* $\left(S^{Z+},b\right)$.

**Proof.** 

I. (1) ∃ compact set $A_{1},A_{2}$, $\tilde{F^{k}}A_{1}⊃\left(A_{1}\cup A_{2}\right)$ and $\tilde{F^{k}}A_{2}⊃\left(A_{1}\cup A_{2}\right)$
  Let $A_{1}=\bar{B\left(a_{1},\varepsilon \right)}\subset {\left[0,1\right]}^{n},A_{2}=\bar{B\left(a_{2},\varepsilon \right)}\subset {\left[0,1\right]}^{n}$, $a_{1}=\left(x_{1}\cdots x_{1}\right)$, $a_{2}=\left(x_{2}\cdots x_{2}\right)$, thus $a_{1},a_{2}$ are the fixed points of $\tilde{F}$. Then we can take a obviously enough small ε, making $A_{1}\cap A_{2}=\phi$ and $A_{1}$, $A_{2}$ compact (bounded closed set).
  ① Let $d_{\max\;}^{\left(1\right)}=\underset{b\in A_{2}}{\sup}d(a,b)<1$, because $\tilde{F}$ is a homeomorphism, $\tilde{F}\left(\partial A_{1}\right)=\partial \tilde{F}\left(A_{1}\right)$. After induction, we know that ∀m⩾1, $\tilde{F^{m}}\left(\partial A_{1}\right)=\partial \tilde{F^{m}}\left(A_{1}\right)$. Therefore, given arbitrarily $y\in \partial \tilde{F}\left(A_{1}\right),d(y,a)=d(\tilde{F}x,a)⩾ld(x,a),$ x∈∂A (namely x is on the sphere). Similarly, after induction, we can obtain $d\left(\tilde{F_{}^{m}}x,a\right)⩾l^{m}\cdot d(x,a)$.
    Let $m_{1}=\left[\log_{l}\frac{d_{\max}^{\left(1\right)}\;}{d(x,a)}\right]+1$, $d\left({\tilde{F}}_{}^{m_{1}}x,a\right)⩾l^{\left[\log_{l}\frac{d_{\max}^{\left(1\right)}}{d(x,a)}\right]+1}\;,d(x,a)⩾\frac{d_{\max\;}^{\left(1\right)}}{d(x,a)}\cdot d(x,a)=d_{\max\;}^{\left(1\right)}$, then ∀ $y\in \tilde{F^{t}}\left(\partial A_{1}\right),t⩾m_{1},d\left({\tilde{F}}_{}^{t}x,a\right)>d\left({\tilde{F}}_{}^{m_{1}}x,a\right)⩾d_{\max\;}^{\left(1\right)}$, namely, the distance between the boundary of $\tilde{F^{t_{1}}}A_{1}$ and $a_{1}$ is more than $d_{\max}^{\left(1\right)}$. Meanwhile, because the homeomorphism $\tilde{F}$ keeps the simply connected feature (homeomorphism ⇒ homotopy ⇒ same Fundamental group ⇒ simply connected), $\tilde{F^{t}}A_{1}$ is still a closed manifold, and all points inside $\partial \tilde{F^{t}}A_{1}$ still belong to $\tilde{F^{t}}A_{1}$, thus $A_{2}\subseteq B$ $\left(a_{1},d_{\max\;}^{\left(1\right)}\right)\subseteq \tilde{F^{t}}A_{1}$ and $A_{1}\subseteq B\left(a_{1},d_{\max\;}^{\left(1\right)}\right)\subseteq \tilde{F^{t}}A_{1}$, namely, $A_{1}\cup A_{2}\subseteq \tilde{F^{t}}A_{1}$. Therefore, the first conclusion is proved.
  ② Same with ①, we can find $m_{2}=\left[\log_{l}\frac{d_{\max}^{\left(1\right)}}{d(x,b)}\right]+1$, wherein $d_{\max}^{\left(2\right)}=\underset{a\in A_{1}}{\sup}d\left(a,a_{2}\right)$, so $\forall t⩾m_{2}$, $A_{1}\cup A_{2}\subseteq \tilde{F^{t}}A_{2}$
  To synthesize ①②, let $k=\max\left(m_{1},m_{2}\right)$, then $A_{1}\cup A_{2}\subset \tilde{F^{k}}A_{1},$ $A_{1}\cup A_{2}\subset \;\tilde{F^{k}}A_{2}$.
II. $A_{1}$, $A_{2}$ can ‘shrink to less than or equal to a point’.

Because $1=||I||=\left\|\tilde{F^{-1}}\cdot F\right\|=\left\|\tilde{F^{-1}}\right\|\cdot \left\|\tilde{F}\right\|$, $\left\|\tilde{F^{-1}}\right\|=\frac{1}{\left\|\tilde{F}\right\|}<1$, we know $\forall x,y\in d\left(\tilde{F_{}^{-1}x},\;\tilde{F_{}^{-1}y}\right)<d(x,y)$, then $\forall \tilde{F^{-k}}x\in \tilde{F^{-k}}A_{1},\tilde{F^{-k}}y\in \tilde{F^{-k}}A_{2},d\left(\tilde{F^{-k}}x,(\alpha )\right)<d\left(x,{\left(a\right)}_{i\in I}\right)$ and $d\left(\tilde{F^{-k}}y,(b)\right)<d(y,(b))$, so $\tilde{F^{-k}}A_{1}\cap \tilde{F^{-k}}A_{2}=\phi$, $N\left(\tilde{F^{-k}}A_{1}\cap \tilde{F^{-k}}A_{2}\right)⩽1$ is satisfied. From the theorem in [51], the conclusion holds. □

**Theorem** **A8.** *For* $M_{3}$*, if* $\tilde{F}$ is a expanding mapping*, P{([0, 1]^n^, Ω,* $\tilde{F}$*) has* L−Y *chaos} = 1, and ([0, 1]^n^, Ω,* $\tilde{F}$*) does not have quasi-consensus.*

**Proof.**  From Lemma A12, for $M_{3}$, $P\left\{w:\exists \tilde{F}\exists T,\forall t>T,F\left(w_{t}\right)=\tilde{F}\right\}=1$. Then, given w in this set, $\tilde{F}$ is fixed. Let $\tilde{x(0)}=x(T+1)$, we know the limit feature of $\tilde{x}(t)$ is same with x(t). The following deduction is derived and based on the given $w,\tilde{F},x(0)$; Let the Lebesgue measure of ${\left[0,1\right]}^{n}$ written as $m:B\left({\left[0,1\right]}^{n}\right)\to [0,1]$, for the measurable mapping $\tilde{F}$, we construct a invariant measure $u(A)=\underset{n}{\lim}\frac{1}{n}\sum_{i=1}^{n}m\left({\tilde{F}}^{-1}A\right)$. Topological entropy in the following part are based on the $\tilde{F}$-invariant measure u.

① $\left({\left[0,1\right]}^{n},d\right)$ is compact and $\tilde{F}$ is continuous.
  For ${\left[0,1\right]}^{n}$, because ${\left[0,1\right]}^{n}\subset B(0,\sqrt{n}).\;$ and ${\left[0,1\right]}^{n}$ is a closed set (under the metric topology), ${\left[0,1\right]}^{n}$ can be a metric subspace of $R^{n}$ based on the sub-topology/sub-metric. Thus, $\left({\left[0,1\right]}^{n},d\right)$ is compact;
  For $\tilde{F}$, we know ${\left(\tilde{F}\right)}_{i}=L\circ {\left(F\right)}_{i}.F_{i}$ is the component of linear mapping, and is obviously continuous. $L=\left\{\begin{array}{cc} x, & x\in (0,1)\\ 0, & x\leq 0\\ 1, & x\geq 1 \end{array}\right.$ is also continuous, so $\forall i,{\left(\tilde{F}\right)}_{i}$ is continuous. Therefore $\tilde{F}$ is continuous.
② $\tilde{F}\mathrm{is}\;\mathrm{expanding}$, then $\tilde{F}$ is expansive and $h(\tilde{F})>0$.
  From Lemma A12, if $\tilde{F}$ is expanding, then $\tilde{F}$ is expansive. Then, from Lemma A14, we know $h(\tilde{F})=\frac{1}{k}h\left(\tilde{F^{k}}\right)=\frac{1}{k}h(\sigma )=\frac{1}{k}\mathrm{lg}2>0$
③ $\left({\left[0,1\right]}^{n}\tilde{,F}\right)$ has L−Y chaos and does not have quasi-consensus.
  From $h\left(\tilde{F}\right)>0$ and the theorem in [52], $\left({\left[0,1\right]}^{n}\tilde{,F}\right)$ has L−Y chaos. Then, from Lemma A13, it does not have quasi-consensus.
  In conclusion, from Lemma A11, we know P{([0, 1]^n^, Ω, $\tilde{F}$) has L−Y chaos} = 1, and ([0, 1]^n^, Ω, $\tilde{F}$) does not have quasi-consensus. □

## Appendix C. The Methods for Estimation and Test

### Appendix C.1. The Method for Estimation

The estimation method for our model consists of three steps: (1) employing approximate Bayesian computation (ABC), we obtain a posterior distribution sample of parameters (u_i_/d_i_/k_i_) by leveraging both real data and model simulation outcomes; (2) utilizing kernel density estimation, we smooth the empirical distribution of parameters into a posterior density function; (3) employing Markov Chain Monte Carlo (MCMC), we compute various integrals of the density function to obtain posterior estimates. The detailed procedure is discussed below.

Regarding (1), we can adopt the conventional approach of approximate Bayesian computing, which necessitates the initial random selection of multiple values for u_i_/d_i_/k_i_, followed by the computation of simulation results under these parameter values. If the discrepancy between the results and the actual data falls below a critical value, denoted as e, the parameter value is deemed acceptable; otherwise, it is rejected. Multiple approaches exist for determining the critical value of e. Our proposed solution involves the implementation of a “sensitivity reduction” strategy: (1) Initially, we select a decreasing sequence of e values and substitute them into the model to obtain the posterior distribution of the parameters. (2) Once the impact of decreasing the value of e on the posterior distribution becomes negligible, we halt the calculation process and employ the final value as the selected e value.

For (2), given the constrained parameter information (u_i_∈[−1, 1] and d_i_ ∈[0, 1]), we can opt for the standard Gaussian distribution as the kernel function and employ classical techniques to formulate the kernel density. Concurrently, leveraging the computed deviation term of the kernel function, we will utilize the equation $h_{opt}=\kappa_{2}^{-2/5}\kappa^{1/5}{\left\{{\int \left[f^{\left(2\right)}\left(x\right)\right]}^{2}dx\right\}}^{-1/5}n^{-1/5}$, where $\kappa =\int k(v)v^{2}dv$ and $\kappa =\int k^{2}(v)dv$ [53], to ascertain the most suitable bandwidth. This approach enables us to obtain a posterior density, p_i_ (u), which effectively addresses the challenges of both undersmoothing and oversmoothing.

In reference to (3), based on the principles of Markov Chain Monte Carlo (MCMC), we utilize the esteemed Metropolis–Hastings algorithm to facilitate integral computation. Our primary focus revolves around acquiring two essential metrics: the posterior expectation of parameters and Bayesian factors. Specifically, the estimation of the posterior expectation of variable “u” entails evaluating $\int_{-1}^{1}u\cdot p_{i}\left(u\right)du$. Furthermore, the Bayesian factor encapsulates the probability of “u” assuming values within a predetermined range.

For the purpose of posterior expectation estimation, let us illustrate our approach within the framework of MCMC. Initially, we adopt the uniform distribution U(a, b) as our proposed distribution. After selecting the initial value “xt”, we generate prospective values “x’” drawn from U(a, b). Subsequently, we compute the acceptance probability and, based on this likelihood, assign “x’” as “xt + 1”. Analogously, we can obtain a sequence of numerical values {x0……xn} that form Markov chains, characterized by their stationary distribution aligning with the posterior distribution. By virtue of the central limit theorem, the arithmetic mean of this sequence can be regarded as the posterior expectation estimate. Moreover, this method extends its utility to calculate integral results for the Bayesian factor as well.

### Appendix C.2. The Algorithm for Testing

Step1 Obtain the sampling frame of F by simulation, namely $\left\{F\left(w_{i}\right):\omega_{i}\subseteq W\subseteq \Omega \right\},$ N=10000
Step2 Obtain the sampling frame of $T_{1}$ and $T_{2}$, namely $\left.\{T_{1}\left(\omega_{i}\right):||T(\omega_{i})x_{0}-x_{1}||<\varepsilon \right\}$, $\left\{T_{2}\left(\omega_{i}\right)\right.\left.:\left\|T\left(\omega_{i}\right)x_{1}-x_{2}\right\|<\varepsilon \right\},N=10000$
Step3 Obtain samples from the sampling frame of $F,T_{1},T_{2}$, n = 50.
Step4 Test $F\begin{array}{r} D\\ \equiv \end{array}T_{1}$ and $F\begin{array}{r} D\\ \equiv \end{array}T_{2}$, if they all holds, $T\equiv T_{1}\equiv T_{2}$. Thus, F≡T.

### Appendix C.3. Equivalence of Rules(F/T) and Distributions(x^(t)/x(t))

**Proposition** **A4.** *If* $F\begin{array}{r} D\\ \equiv \end{array}T$*, then* (x(0),x(1),x(2)) *and* $\hat{x}\left(0\right),\hat{x}\left(1\right),\hat{x}\left(2\right)$ *have the same distribution.*

**Proof.**  $P\{w:(x(0),\hat{x}(1),\hat{x}(2))⩽(a,b,c)\}$, (“≤” represents the vector order)

$=P\left\{w:\left(x(0),F(\omega )x(0),F^{2}(w)x(0)\right)\leq \left(a,b,c\right)\right\}$

$=P\left\{w:x(0)⩽a,F(\omega )x(0)⩽b,F^{2}(w)x(0)⩽c\right\}$

$=P\left\{\left(w:x(0)⩽a,T(\omega )x(0)⩽b,T^{2}(w)x(0)⩽c\right)\right\}\cap \left\{T(\omega )=F(\omega )\right\}$

$=P\left\{\omega :x(0)⩽a,T(\omega )x(0)⩽b,T^{2}(w)x(0)⩽c\right\}$

$=P\{w:(x(0),x\left(1\right),x(2))⩽(a,b,c)\}$

The last holds but with only one equality because ∀B∈B(Ω), we have that $P\left(A\right)=1,P(B)⩾P(B\cap A)=1-P(\left.B^{c}\cup A^{c}\right)⩾1-\left(P\left(B^{c}\right)+P\left(A^{c}\right)\right)=1-P\left(B^{c}\right)=P(B)$, so P(B)=P(B∩A). Let $B=\left\{\omega :x(0)⩽a,T(\omega )x(0)⩽b,T^{2}(w)x(0)⩽c\right\}$, A={T(ω)=F(ω)}, then the conclusion holds. □
